# Emission Using *Adaptable* Range Separated Hybrids: Thermally Activated Delayed Fluorescence Emitters as Test Case

**DOI:** 10.1002/jcc.70275

**Published:** 2025-11-21

**Authors:** Tianhong Yan, Carlo Adamo, Ilaria Ciofini

**Affiliations:** ^1^ Chimie ParisTech, CNRS, Institute of Chemistry for Life and Health Sciences PSL University Paris France

## Abstract

In this contribution, we describe how our methodology of tuning adaptable range separated hybrids (RSHs), recently developed to accurately predict absorption energy associated with charge transfer (CT) excitations, can be extended to take into account structural relaxation and used to predict emission in molecular systems. To test our model, we have focused on a series of thermally activated delayed fluorescence (TADF) emitters that have been the subject of intense theoretical and experimental investigation. The results obtained for these compounds show the very good accuracy of the adaptive tuning procedure of RSHs also for the prediction of emission energies and their balanced description of triplet and singlet excited states, confirming their potential for the exploration of excited states energy surfaces, including for the design of novel TADF compounds.

## Graphical abstract

A novel approach to tune range separated hybrids based on our recently developed method allowing to predict accurately the energy associated with charge transfer excitations, has been extended to take into account structural relaxation and used to predict emission in a series of thermally activated delayed fluorescence emitters.

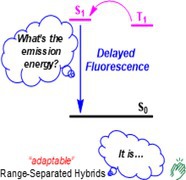

## 1 Introduction

Density functional theory (DFT) [1] and Time Dependent DFT (TD‐DFT) [2, 3] are largely applied to study and predict the properties and reactivity of molecular systems at the ground states (GS) and the excited states (ES) [4]. The success of these methods relies on both the accuracy that can be reached at a moderate computational cost, allowing the study of large compounds in realistic environments, and on the large availability and user‐friendly implementation of these approaches, allowing their use by a community larger than that represented by theoretical chemists [5].

Nonetheless, their main drawback is related to the use of density functional approximations (DFA), which may affect the results obtained quantitatively and qualitatively, leading to inaccurate descriptions and, in the more severe cases, to chemically wrong predictions. A large variety of benchmarks have analyzed the performance of different DFAs for ground [6, 7, 8] and ES [9, 10, 11, 12] properties. In the case of ES, the description of charge transfer (CT) states has been pointed out as particularly challenging for many of the functionals that are generally accurate for other properties, such as, for instance global hybrids [13, 14, 15, 16, 17].

Such a weakness is a serious limitation for the use of DFT and TD‐DFT as predictive tools in the design of novel molecule‐based devices since CT states play a crucial role for many applications, such as photovoltaics [18, 19, 20], electronics—OLED materials [21, 22, 23, 24, 25]—photocatalysis [26], or drug design [27, 28, 29] to mention some of them.

The main sources of errors have been traced back to the use of local exchange and correlation functionals suffering from self‐interaction error (SIE), lacking the correct (−1/*r*) asymptotic behavior for the Kohn–Sham potential and derivative discontinuities [17, 30, 31, 32, 33].

The family of range‐separated functionals (RSH), including full Hartree–Fock (HF) exchange at long range (i.e., the so‐called long range corrected functionals) is recovering the correct asymptotic behavior and offers a solution to enhance the performances of global hybrids [34, 35, 36].

In this case the exchange contribution is computed by splitting the Coulomb operator into a short and a long‐range component as

(1) $$\frac{1}{r_{12}}=\frac{1-\mathrm{erf}\left(\gamma \;r_{12}\right)}{r_{12}}+\frac{\mathrm{erf}\left(\gamma \;r_{12}\right)}{r_{12}}$$

allowing to compute the first term, dominating at short range (SR), by a DFT (GGA or hybrid) functional and the second, leading at long distances (LRs), by an exact (HF) exchange term. The total exchange and correlation energy in this case reads

(2) $$E_{\mathrm{XC}}=\alpha E_{X,\mathrm{HF}}^{\mathrm{SR}}\left(\gamma \right)+\left(1-\alpha \right)E_{X,\mathrm{DFT}}^{\mathrm{SR}}\left(\gamma \right)+E_{X,\mathrm{HF}}^{\mathrm{LR}}\left(\gamma \right)+E_{C,\mathrm{DFT}}$$

where α is the exact HF fraction included at SR and γ is an adjustable parameter that sets the switching distance between the SR and LR regimes. Several studies focused on the definition of, which is in principle system‐independent [37, 38] and literature works showed that the intrinsic difficulty in obtaining a unique γ for GS and ES properties [39, 40, 41].

Focusing exclusively on the recovery of ES properties and more specifically on the evaluation of intermolecular CT energy, Stein et al. proposed an approach to tune the range separation split parameter to recover the exact ionization potential and the electron affinity from Koopman's theorem, leading to the definition of the optimally tuned RSH (OT‐RSH) [42]. This tuning procedure, later on generalized to include the effect of a dielectric simulating the environment [43], is system‐dependent but non‐empirical and has shown to provide accurate results for OT‐RSH, which have since then emerged as an elegant and efficient way of reaching an accurate evaluation of CT excitation energies and are increasingly applied both in solution and the solid state [44]. In this general context, we have recently proposed a different strategy to define the γ parameter by adjusting it to impose a full exact exchange starting for distances comparable to the hole–electron distance observed in the CT states of the systems of interest [45]. This tuned range separation split parameter was used in conjunction with the PBE0 global hybrid [46] at SR to define the LC‐PBE0* functional. Compared to the corresponding global hybrid (PBE0) and non‐tuned RSH approach (i.e., the standard LC‐PBE and the previously proposed LC‐PBE0 functional [47]), the LC‐PBE0* functional overperforms them in estimating CT ES vertical absorption energies.

**SCHEME 1:**
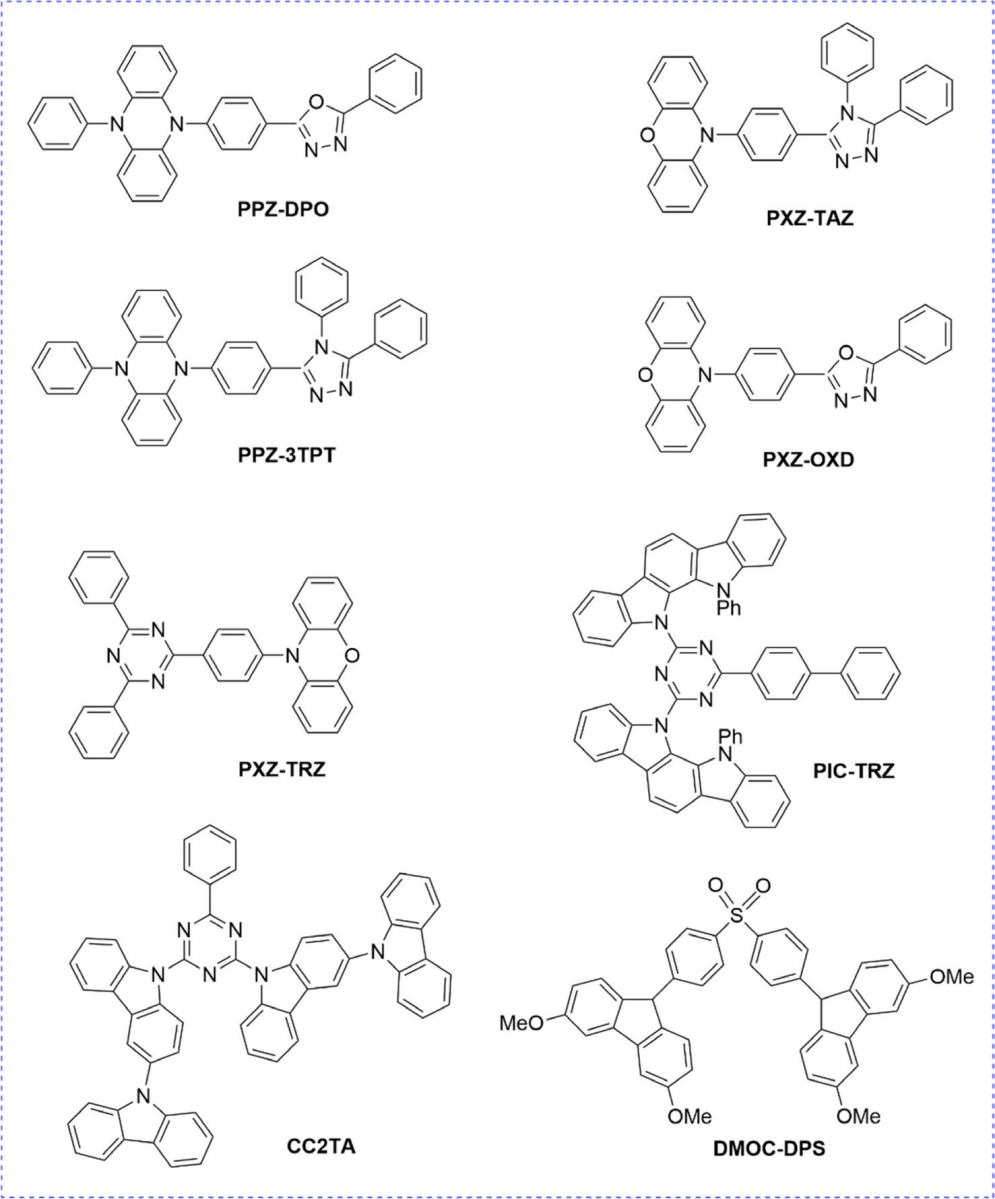
Schematic representation of the systems investigated.

In this work, we have extended our approach to include the treatment of relaxed ES, allowing for the simulation of emission energies and structures. To test the performance of our model, we focus on a set of eight thermally activated delayed fluorescence (TADF) emitters, depicted in Scheme 1, among those developed by Lee and collaborators [22, 23, 24, 25] and lately subject to different theoretical studies [48, 49].

TADF is a push–pull (donor–acceptor) system designed to obtain a minimal gap between the lowest singlet ES and the lowest triplet ES, allowing for efficient reverse intersystem crossing (RISC, *T*_1_ → *S*_1_) responsible for the observed TADF.

This condition is reached by designing systems characterized by a CT singlet ES (*S*_1_) characterized by a hole and electron localized on orbitals with minimal exchange interaction leading to minimizing the corresponding singlet–triplet energy gap (Δ*E*_*T*1→*S*1_). Experimentally, this can be obtained in *π* conjugated organic compounds characterized by pseudo‐orthogonal donor and acceptor moieties. The orthogonality reduces the overlap of the hole and the electron charge distributions at the ES and thus minimizes the singlet‐triplet gap.

Previous works have shown that TADF emitters represent a challenging test case for standard DFA [22, 23, 24, 25, 48, 49, 50, 51]. Indeed, both global and standard range‐separated hybrids showed major problems in quantitatively predicting their singlet ES energies and a rich literature can be found aiming at developing computationally efficient and accurate methods to describe not only the ES CT singlet energy but also the corresponding singlet‐triplet energy gap [48, 49, 50, 51].

The paper is structured as follows: after a description of the adaptive tuning approach for emission and the computational setup (Section 2), the results obtained are presented and discussed (Section 3) and some general conclusions and perspectives are given (Section 4).

## 2 The Method and the Computational Setup

**SCHEME 2:**
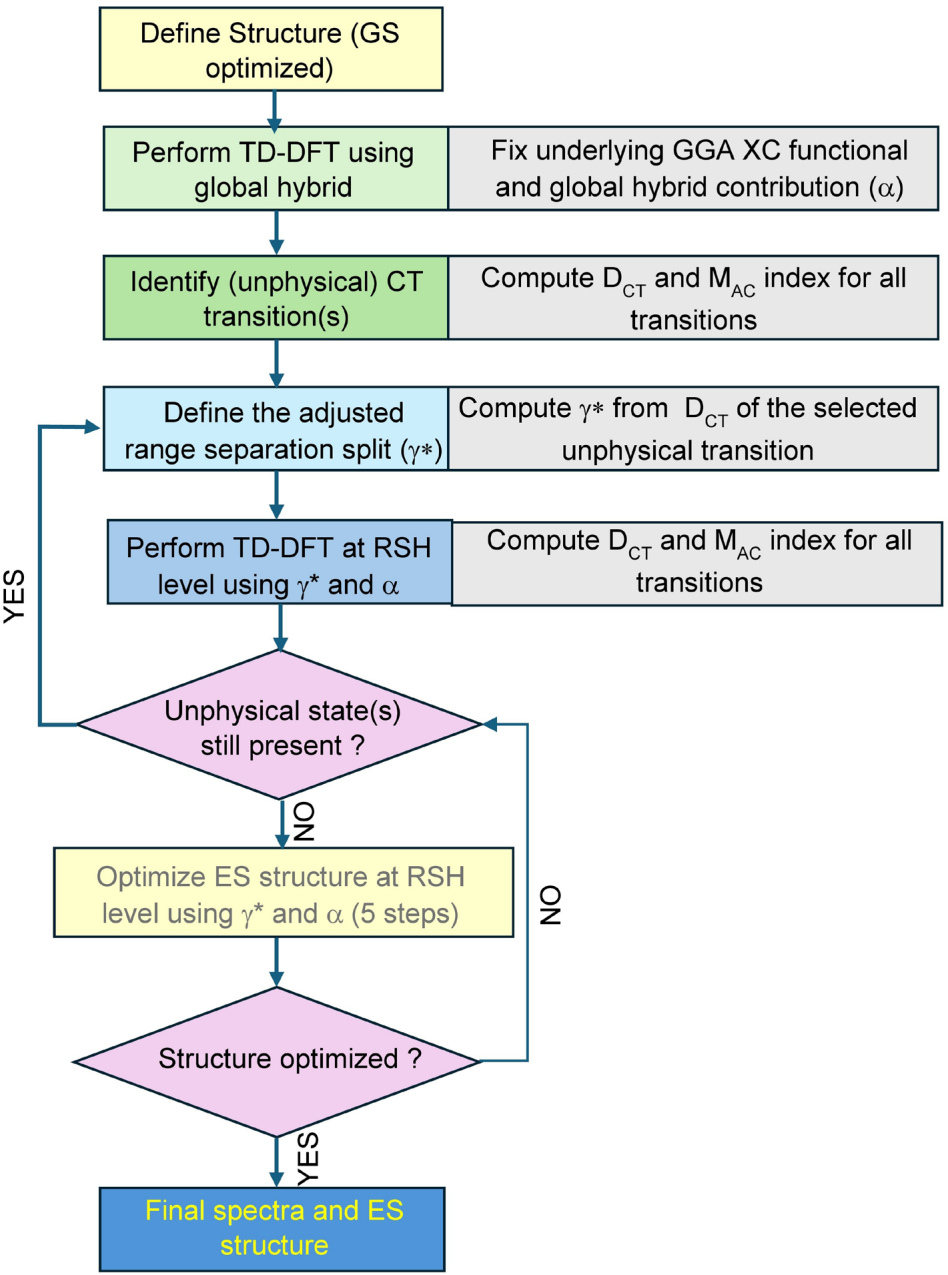
Schematic workflow describing the use of adaptable RSH for vertical absorption and emission. Whenever the *M*_AC_ index is computed, an estimate of the KS orbital energies at HF level is also necessary. In the case of triplets, the TDA is always used.

To explore excited singlet and triplet potential energy surfaces, we rely on our recently developed procedure to adjust RSH, described in Reference [45], to which we refer the interested reader for full details and discussion. To summarize, our adjusted RSH reads:

(3) $$E_{\mathrm{XC}}=\alpha E_{X,\mathrm{HF}}^{\mathrm{SR}}\left(\gamma^{*}\right)+\left(1-\alpha \right)E_{X,\mathrm{DFT}}^{\mathrm{SR}}\left(\gamma^{*}\right)+E_{X,\mathrm{HF}}^{\mathrm{LR}}\left(\gamma^{*}\right)+E_{C,\mathrm{DFT}}$$

where to obtain the tuned range split parameter ($\gamma^{*}$), the procedure reported in the internal loop in Scheme 2 is applied. First, a vertical calculation with the global hybrid used at SR is performed and the hole–electron separation for all ES of interest is evaluated by means of the *D*_CT_ density‐based descriptor. Among different metrics developed to give an estimate of intra‐ and intermolecular CT distance [10], the $D_{\mathrm{CT}}$ index [52] was applied. This latter, as defined in the original formulation (Equation 4), measures the CT distance as the spatial distance between the two barycenters ($R^{+}$ and $R^{-}$) of the two (positive and negative) charge density distributions corresponding to the increment and depletion of electron density upon excitation, respectively.

(4) $$D_{\mathrm{CT}}=R^{+}-R^{-}$$

Although ***D***_**CT**_ itself is a vector, and has been used to quantify CT in molecular systems [53, 54], here we will use it to quantify the hole–electron separation and thus only the associated norm (hereafter labeled as $D_{\mathrm{CT}}$) will be considered. The *D*_CT_ index is next used to evaluate the *M*_AC_ (Mulliken averaged configuration) index [55, 56] for each of the computed transitions. This index is used to assess the reliability of the computed ES. The *M*_AC_ is derived from the Mulliken expression of intermolecular CT energy [57], reported in Equation (5). Indeed, following the Mulliken expression, the energy associated with a net one‐electron intermolecular CT ($\omega_{\mathrm{CT}}$) between a donor (*D*) and an acceptor (*A*) moiety can be expressed as

(5) $$\omega_{\mathrm{CT}}=IP_{D}-EA_{A}-\frac{1}{R}$$

$IP_{D}$, $EA_{A}$ being the ionization potential of the donor, the electron affinity of the acceptor and *R* is the distance between the donor and the acceptor, respectively. Using a Koopmans‐type approach, we have proposed the *M*_AC_ index [55, 56], approximating $\omega_{\mathrm{CT}}$, by replacing *R* by the computed CT distance (given here by the *D*_CT_) and $IP_{D}$ and $EA_{A}$ from the weighted average of the energies of the initial occupied ($\epsilon_{i}$) and final virtual ($\epsilon_{a}$) Kohn–Sham orbitals associated to the transition as

(6) $$M_{\mathrm{AC}}=\frac{\sum_{\mathrm{ia}}\;\left[{C_{\mathrm{ia}}}^{2}\left(\epsilon_{a}^{\mathrm{DFT}-\mathrm{HF}}-\epsilon_{i}^{\mathrm{DFT}-\mathrm{HF}}\right)\right]}{\sum_{\mathrm{ia}}\;{C_{\mathrm{ia}}}^{2}}-\frac{1}{D_{\mathrm{CT}}}$$

where the weights $c_{\mathrm{ia}}$ are the CI coefficients obtained as solution of TD‐DFT equations. In Equation (6), the energies of the orbitals obtained using DFT theory ($\epsilon^{\mathrm{DFT}-\mathrm{HF}}$) are corrected with a single SCF cycle using Hartree–Fock theory. By comparing the so computed *M*_AC_ values with the transition energies computed at TD‐DFT level of theory allows to define which states are erroneously described (Equation 7) and to identify the range that should be used to introduce full HF exchange

$$E^{\mathrm{TD}-\mathrm{DFT}}>M_{\mathrm{AC}}\to \text{Real state},$$

(7) $$E^{\mathrm{TD}-\mathrm{DFT}}<<M_{\mathrm{AC}}\to \text{Unphysical state}$$

As detailed in the original paper [45], a range separation parameter corresponding to half of the maximal hole–electron separation computed for ghost states is used (Equation 8):

(8) $$\gamma^{*}=\frac{1}{\frac{D_{\mathrm{CT}}^{\text{ghost}-\max}}{2}}=\frac{2}{D_{\mathrm{CT}}^{\text{ghost}-\max}}$$

Since the energy of the ES could change during the ES optimization, after each five geometry optimization steps, a control on the $\gamma^{*}$ value is performed and its value is re‐adjusted on the basis of the maximal hole–electron separation computed for ghost states, if necessary.

For clarity, the overall workflow corresponding to the tuning methodology for emission is schematically reported in Scheme 2.

The procedure depicted is applied to optimize the lowest singlet ES (*S*_1_) and the last $\gamma^{*}$ value is used to perform TD‐DFT calculations (optimization) of the lowest triplet ES (*T*_0_). Of note, in all cases considered the γ* value optimized in absorption was also enabling to reproduce correctly all ES at the geometry optimized for the first ES. In order to avoid triplet instability issues [58] and as already reported in literature [59, 60], the Tamm–Dancoff approximation (TDA) was also applied to obtain reliable triplet energies and singlet‐triplet energy gaps also in the case of TADF systems [61, 62].

In this work, the following DFAs have been compared: (i) the PBE0 global hybrid casting a fixed percentage of HF exchange (25%) in the PBE [63] exchange correlation functional; (ii) the standard LC‐PBE functional corresponding at SR to the PBE GGA exchange functional (*α* = 0 in Equation 2) and at LR to 100% HF exchange always in conjunction with the PBE correlation functional using its γ default value (γ = 0.470); and (iii) the adjusted RSH—hereafter labeled as LC‐PBE0* functional constructed casting the PBE0 functional at SR (*α* = 0.25) and 100% HF exchange at LR always in conjunction with the PBE correlation functional and using a system dependent $\gamma^{*}$ computed from the *D*_CT_ evaluated at PBE0 level as described above and in Reference [45]. The People triple zeta basis set (6‐311G(d)) has been used. Additional calculations using the 6‐31+G(d) basis set have been performed in the case of the PPZ‐3TPT molecule to test the effect of the basis set and allow a more straightforward comparison with the data in literature. For the same molecule, solvent effects (toluene) were also included by means of the polarizable continuum model using the integral equation formalism with SMD model [64] to allow for a better understanding of its effects on the predicted energies.

All DFT, TD‐DFT and *D*_CT_ calculations were performed with the Gaussian code [65] while the *M*_AC_ index was evaluated with an in‐house code. Apart from the above‐cited case, solvent effects were not included and unrelaxed densities of the ES have been used to evaluate the *D*_CT_. When not differently specified, calculations were performed on minimal energy structures obtained at the same level of theory (i.e., using the same DFA).

## 3 Results and Discussion

In literature, different approaches have been developed to provide an accurate singlet–triplet gap of TADF molecules. Lee and collaborators, for instance, rely on the estimation of the 0–0 energy difference between the excited singlet and triplet states, deriving them from vertical transition energies estimated with an adjusted global hybrid functional. This approach has the advantage of being particularly computationally efficient and quite accurate [22, 23, 24, 25]. Hait and collaborators tested different approaches comparing the use of vertical and relaxed singlet and triplet energies computed both at the TD‐DFT and ROKS levels, showing that relaxed ROKS approaches are particularly efficient in combination with popular global hybrids with low HF exchange [49].

Other authors, such as Penfold [66] and Sun et al. [61], showed how tunable range separated hybrids reveal particularly efficient in predicting singlet‐triplet energy gaps in TADF molecules. In this work, we will follow a strategy similar to that already described in the literature [61, 67] estimating both vertical singlet–triplet energy gap (i.e., using the GS optimized structure) and adiabatic Singlet‐Triplet energy gap defined as the difference in total energy between the lowest relaxed excited singlet and the lowest triplet state. These two energy will be referred as ${∆E}_{S1-T1}^{V}$ and ${∆E}_{S1-T1}^{\text{adia}}$, respectively.

### 3.1 Vertical Singlet and Triplet ES

First of all, Table 1 reports the vertical ES energies computed at the different levels of theory. For these TADF systems (Scheme 1), an estimate of the vertical absorption energy derived from experiments [22, 23, 24, 25] and theoretical approaches [48, 49, 50, 51] is actually available. The full list of computed ES is given in Table S1. Although our direct comparison of vertical transitions and experimental absorption maxima does not correctly take into account the solvent effect or the difference between vertical and adiabatic energy, it allows us to get a first idea of the accuracy of the different DFAs.

**TABLE 1:** Vertical transition energies (*E*_V_, eV), charge transfer distances (*D*_CT_, Å) and *M*_AC_ index (in eV) computed at TD‐DFT for the first singlet (*S*_1_) and triplet (*T*_0_) excited states using different levels of theory and the 6‐311G(d) basis set. The range separation parameter (γ*, Bohr^−1^) used for each system at LC‐PBE0* is reported. For LC‐PBE calculations, the standard *γ* value (*γ* = 0.470 Bohr^−1^) is used. Full data for the first 10 ES computed is given in Reference [45] for PXZ‐OXD, PXZ‐TAZ, PPZ‐3TPT, and PPZ‐DPO and reported in Data S1 for all other systems. The mean absolute deviation with respect to the experimental values (MAD, in eV) as well as the signed deviation range (Dev. (min/max) in eV) are also provided for each functional in the case of the singlet state.

	Exp.	PBE0		LC‐PBE		LC‐PBE0*		
		*E*_V_ (S_1_)	*D*_CT_ (*S*_1_)/*M*_AC_ (*S*_1_)	*E*_V_ (*S*_1_)	*D*_CT_ (*S*_1_)/*M*_AC_ (*S*_1_)	*E* (*S*_1_)	*D*_CT_ (*S*_1_)/*M*_AC_ (*S*_1_)	γ*
PPZ‐3TPT^a^	3.34 [25]	2.51	5.795/5.83	3.94	0.160/LE	3.00	4.849/5.57	0.105
PPZ‐DPO^a^	2.78 [25]	2.11	6.204/5.50	3.95	0.192/LE	2.59	5.270/5.32	0.095
PXZ‐OXD^a^	3.18 [22]	2.52	5.840/6.00	4.47	0.209/LE	2.94	4.987/5.70	0.094
PXZ‐TAZ^a^	3.33 [22]	2.91	5.425/6.30	4.45	0.267/LE	3.35	4.578/5.96	0.107
PXZ‐TRZ	2.73 [24]	2.29	6.377/5.95	4.31	4.292/5.53	2.93	5.369/5.68	0.115
PIC‐TRZ	3.35 [24]	3.02	6.616/6.13	4.45	0.453/LE	3.90	2.177/2.50	0.159
CC2TA	3.64 [24]	3.16	7.760/6.82	4.74	0.514/LE	3.75	4.399/5.69	0.101
DMOC‐DPS	3.35 [23]	3.21	3.848/5.21	4.38	0.259/LE	3.81	2.597/3.72	0.141
MAD		0.496		1.124		0.264		
Dev. (min/max)		−0.83/−0.14		0.6/1.58		−0.34/0.55		

	PBE0		LC‐PBE		LC‐PBE0*		
	*E*_V_ (*T*_1_)	*D*_CT_ (*T*_1_)/*M*_AC_ (*T*_1_)	*E*_V_ (*T*_1_)	*D*_CT_ (*T*_1_)/*M*_AC_ (*T*_1_)	*E*_V_ (T_1_)	*D*_CT_ (*T*_1_)/*M*_AC_ (*T*_1_)	γ*
PPZ‐3TPT	2.44	0.364/LE	2.44	0.097/LE	2.61	0.180/LE	0.105
PPZ‐DPO	2.10	6.170/5.48	2.44	0.095/LE	2.57	5.025/5.45	0.095
PXZ‐OXD	2.51	5.804/5.99	2.69	0.166/LE	2.68	3.625/5.22	0.094
PXZ‐TAZ	2.77	0.087/LE	2.69	0.168/LE	2.89	0.180/LE	0.107
PXZ‐TRZ	2.28	6.348/5.94	2.62	0.134/LE	2.73	4.860/5.72	0.115
PIC‐TRZ	2.78	0.757/LE	2.58	0.091/LE	2.89	0.218/LE	0.159
CC2TA	2.98	5.231/6.92	2.74	0.210/LE	3.09	1.276/LE	0.101
DMOC‐DPS	2.85	3.046/4.54	2.72	0.268/LE	2.98	0.371/LE	0.141

Abbreviation: LE, locally excited state.

^a^ Computed values from Reference [45].

**FIGURE 1:**
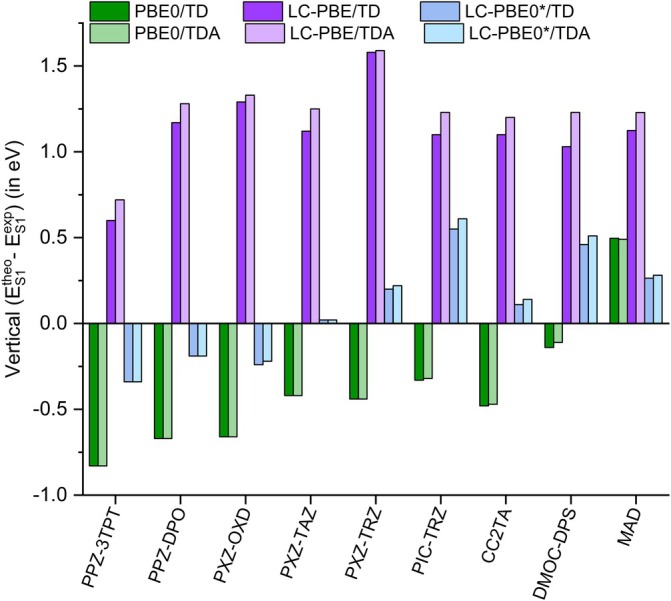
Deviation (in eV) with respect to experimental vertical absorption energy of the vertical transition energies computed at TD and TDA level for the first singlet excited state (*S*_1_) using different levels of theory and the 6‐311G(d) basis set. Refer to Scheme 1 for molecular systems labeling. MAD represents the mean absolute deviation with respect to the experimental values computed on the full set of molecules.

As already commented in our previous work [45], in the case of PXZ‐OXD, PXZ‐TAZ, PPZ‐3TPT, and PPZ‐DPO, these ESs, independently of the level of theory applied, essentially correspond to a HOMO‐LUMO excitation. Furthermore, while PBE0 predicts a sizable CT character (with *D*_CT_ *s* ranging between 5.4 to 6.2 Å), LC‐PBE predicts a very local transition in all cases (with *D*_CT_ *s* all below 0.3 Å). From an energetic point of view, the vertical *E* (*S*_1_) energies predicted at PBE0 level are all significantly underestimated, the differences between the *M*_AC_ estimates and the TD‐DFT energies being all well above 3.0 eV. This demonstrates that these states are actually strongly energetically underestimated at PBE0 level, in agreement with the large differences observed between the experimental and theoretical PBE0 energies. LC‐PBE, on the other hand, strongly overestimates the vertical energies. Using the *D*_CT_ adjusted range separation split parameter in the case of the LC‐PBE0* functional allows to obtain transition energies in good agreement with the experimental value. These general trends can be translated to the results obtained for PXZ‐TRZ, PIC‐TRZ, CC2TA, and DMOC‐DPS. While PBE0 tends to overestimate the CT character and underestimate the transition energy, LC‐PBE tends to overestimate the local character of the transition and gives overestimated energies. LC‐PBE0* offers a more balanced (and overall more accurate) description of the transition character and energies. As a consequence, the mean absolute error in the case of LC‐PBE0* is 0.26 eV, well below both the PBE0 (0.50 eV) and the LC‐PBE (1.12 eV) values. Of note, actually the PIC‐TRZ molecule appears to be extremely problematic for the LC‐PBE0* functional with a difference of 0.55 eV between the predicted and he experimental absorption maximum. Actually, as it can be seen from the NTO reported in Figures S1–S4, in this case, the hole is localized on the peripheral phenyl‐dihydroindolo[2,3‐a]carbazole substituents while the electron is localized on the substituted triazole core. Since the peripheral substituents are symmetric with respect to this latter, the computed barycenter of hole charge distribution as used in the *D*_CT_ definition (Equation 4) is closer to the barycenter of the charge electron distribution than each of the barycenter of the hole computed on each substituent. In other words, the *D*_CT_ underestimates the CT distance, resulting in a large $\gamma^{*}$ value used in the LC‐PBE0* formulation (0.159 Bohr^−1^) and consequently a very high predicted excitation energy. Of note this problem is the same as what was already pointed out in the case of the 10‐phenyl‐10*H*‐spiro[acridine‐9,9′‐fluorene]‐2′,7′‐dicarbonitrile (i.e., ACRFLCN) molecule in our original paper and stress again the importance of having a realistic evaluation of the CT distance in the tuning of the functionals. Indeed, with this exception, LC‐PBE0* represents an improvement with respect to both LC‐PBE and PBE0* results. This is also evident from the computed errors and mean absolute deviation (MAD) in absorption energies as reported in Figure 1.

Applying the TDA approximation (Table 2) for the calculation of both singlet and triplet ES does not significantly affect the general trend discussed for using the TD approach in terms of the nature of the excitations and behavior of the different functionals. The effect of the TDA on the singlet states is actually negligible, also in terms of their energy.

**TABLE 2:** Vertical transition energies (*E*_V_, eV), charge transfer distances (*D*_CT_, Å) and *M*_AC_ index (in eV) computed at TDA level for the first singlet (*S*_1_) and triplet (*T*_0_) excited states using different levels of theory and the 6‐311G(d) basis set. The range separation parameter (γ*, Bohr^−1^) used for each system at LC‐PBE0* is reported. For LC‐PBE calculations, the standard *γ* value (*γ* = 0.470 Bohr^−1^) is used.

	PBE0		LC‐PBE		LC‐PBE0*		
	*E*_V_ (S_1_)	*D*_CT_ (*S*_1_)/*M*_AC_ (*S*_1_)	*E*_V_ (*S*_1_)	*D*_CT_ (*S*_1_)/*M*_AC_ (*S*_1_)	*E*_V_ (*S*_1_)	*D*_CT_ (*S*_1_)/M_AC_ (S_1_)	γ*
PPZ‐3TPT	2.51	5.799/5.83	4.06	0.166/LE	3.00	4.853/5.57	0.105
PPZ‐DPO	2.11	6.207/5.50	4.06	0.362/LE	2.59	5.272/5.32	0.095
PXZ‐OXD	2.52	5.843/6.00	4.51	3.877/5.49	2.96	5.005/5.71	0.094
PXZ‐TAZ	2.91	5.428/6.30	4.58	0.253/LE	3.35	4.588/5.97	0.107
PXZ‐TRZ	2.29	6.379/5.95	4.32	4.302/5.53	2.95	5.386/5.69	0.115
PIC‐TRZ	3.03	6.657/6.14	4.58	0.870/LE	3.96	2.556/3.46	0.159
CC2TA	3.17	7.754/6.82	4.84	1.036/LE	3.78	4.608/5.81	0.101
DMOC‐DPS	3.24	3.814/5.18	4.58	0.338/LE	3.86	2.736/3.98	0.141

	PBE0		LC‐PBE		LC‐PBE0*		
	*E*_V_ (*T*_1_)	*D*_CT_ (*T*_1_)/*M*_AC_ (*T*_1_)	*E*_V_ (*T*_1_)	*D*_CT_ (*T*_1_)/*M*_AC_ (*T*_1_)	*E*_V_ (*T*_1_)	*D*_CT_ (*T*_1_)/*M*_AC_ (*T*_1_)	γ*
PPZ‐3TPT	2.50	5.759/5.82	3.08	0.124/LE	2.81	0.237/LE	0.105
PPZ‐DPO	2.10	6.176/5.49	3.09	0.121/LE	2.58	5.243/5.31	0.095
PXZ‐OXD	2.51	5.810/5.99	3.40	0.219/LE	2.79	4.584/5.58	0.094
PXZ‐TAZ	2.90	5.389/6.29	3.39	0.228/LE	3.13	0.152/LE	0.107
PXZ‐TRZ	2.28	6.353/5.94	3.32	0.178/LE	2.79	5.125/5.63	0.115
PIC‐TRZ	2.93	5.644/5.86	3.39	0.109/LE	3.21	0.139/LE	0.159
CC2TA	3.09	7.080/6.74	3.61	0.368/LE	3.39	3.388/5.55	0.101
DMOC‐DPS	2.95	3.537/5.05	3.41	0.190/LE	3.12	0.354/LE	0.141

Abbreviation: LE, locally excited state.

On the other hand, as expected, a systematic increase in the energy of the triplet states is obtained using the TDA approach, in particular for the RSHs (both LC‐PBE and LC‐PBE0*).

This effect is fully in line with previous works correlating the percentage of HF exchange in the functional with triplet instability, that is triplet energy underestimation, when using the TD formalism. The overall trends for the singlet state are not affected: the LC‐PBE0* functional still provides the most accurate estimates of vertical transition energies, with a mean absolute error of 0.28 eV.

Since the tuning parameter is also practically unaffected (difference of less than 0.02 Bohr^−1^) by the use of TD or TDA approximation, the TD values have been used. On the other hand, the triplet energies in the case of RSH are significantly modified at the TDA level and the vertical singlet‐triplet gap is affected by the use of the TD or TDA approaches, as it will be discussed in Section 3.2. Of note, the MAD in vertical absorption energies of LC‐PBE0* (in the absence of solvent effect) is not only smaller than that of PBE0 and those previously reported for other global hybrids, but significantly smaller than that of RSH and comparable with that obtained (including solvent) using OT procedures in RSH [61]. The use of LC‐PBE0* also enables an increase in the correlation between computed and experimental vertical absorption, especially when compared to the LC‐PBE behavior.

Finally, in order to get an idea of the effect of basis set and solvent on the computed vertical energies, we performed calculations for the PPZ‐3TPT molecule, also including solvent using the SMD model [64] or using the 6‐31+G(d) basis, which has been largely used in the literature to describe these systems. The results obtained are reported in Table S2. In this case, effects on both GS geometrical and electronic structures were considered since the GS geometry was consistently reoptimized at each level of theory. The effect of the basis set is found to be negligible with maximal differences of 0.05 eV in both absolute singlet and triplet energies and the singlet‐triplet gap. The effect of the inclusion of the solvent is slightly larger, with a maximal difference in the singlet energy of 0.1 eV and an effect on the gap of 0.16 eV in the case of the LC‐PBE0* functional. Nonetheless, also in this case, the general conclusions concerning the agreement with the experimental data do not change, and the inclusion of the solvent simply ameliorates the agreement with the experimental data, the LC‐PBE0* functional being still the most accurate.

### 3.2 Singlet and Triplet Relaxation and Singlet‐Triplet Energy Gap

In Table 3, the results concerning relaxed triplet and singlet states obtained using the TDA approach are collected. Indeed, using the TD approach to optimize the structure results in unphysically low triplet states (as already reported in the literature), as reported in Table S3.

**TABLE 3:** Excited state energies (*E*, eV), oscillator strengths (*f*, au), charge transfer distances (*D*_CT_, Å), and *M*_AC_ index (in eV) computed for the first singlet (*S*_1_) and triplet (*T*_1_) excited states computed at TDA level on TDA optimized structures using different levels of theory and the 6‐311G(d) basis set. The mean absolute deviation with respect to the experimental values (MAD, in eV) as well as the signed deviation range (Dev. (min/max) in eV) are also provided for each functional in the case of the singlet state.

	PBE0		LC‐PBE		LC‐PBE0*		Exp.
	*E* (*S*_1_)	*D*_CT_ (*S*_1_)/*M*_AC_ (*S*_1_)	*E* (*S*_1_)	*D*_CT_ (*S*_1_)/*M*_AC_ (*S*_1_)	*E* (*S*_1_)	*D*_CT_ (*S*_1_)/*M*_AC_ (*S*_1_)	*E* (*S*_1_)
PPZ‐3TPT	1.85	6.262/5.02	3.25	0.046/LE	2.31	5.084/4.76	2.35 [25]
PPZ‐DPO	1.58	6.210/4.78	3.26	0.047/LE	1.98	5.334/4.56	2.15 [25]
PXZ‐OXD	2.02	5.898/5.34	3.78	0.069/LE	2.40	5.112/5.12	2.50 [22]
PXZ‐TAZ	2.29	5.877/5.57	3.77	0.118/LE	2.65	4.723/5.17	2.72 [22]
PXZ‐TRZ	1.80	6.154/5.21	3.64	4.044/4.75	2.35	5.313/4.99	2.27 [24]
PIC‐TRZ	2.01	7.131/4.99	4.36	0.598/LE	3.19	5.678/4.95	2.52 [24]
CC2TA	2.10	7.268/5.39	4.56	2.371/3.32	2.63	6.100/5.11	2.63 [24]
DMOC‐DPS	2.31	5.553/5.59	4.30	0.482/LE	2.89	4.531/5.09	2.79 [23]
MAD	0.496		1.374		0.154		
Dev. (min/max)	−0.57; −0.43		0.9/1.93		−0.17/0.67		

	PBE0		LC‐PBE		LC‐PBE0*		Exp.
	*E* (*T*_1_)	*D*_CT_ (*T*_1_)/*M*_AC_ (*T*_1_)	*E* (*T*_1_)	*D*_CT_ (*T*_1_)/*M*_AC_ (*T*_1_)	*E* (*T*_1_)	*D*_CT_ (*T*_1_)/*M*_AC_ (*T*_1_)	
PPZ‐3TPT	1.84	6.223/5.01	2.36	0.037/LE	2.18	0.064/LE	LE
PPZ‐DPO	1.57	6.183/4.77	2.36	0.010/LE	1.98	5.130/4.56	
PXZ‐OXD	2.01	5.871/5.33	2.70	0.108/LE	2.20	3.437/4.00	
PXZ‐TAZ	2.23	4.520/4.96	2.52	0.367/LE	2.53	0.061/LE	
PXZ‐TRZ	1.79	6.132/5.20	2.69	0.112/LE	2.28	4.644/4.99	CT
PIC‐TRZ	2.00	7.010/5.02	2.46	0.578/LE	2.70	0.834/LE	LE
CC2TA	2.04	6.940/5.34	2.89	0.310/LE	2.78	1.600/LE	LE
DMOC‐DPS	2.33	5.493/5.62	2.81	0.328/LE	2.73	0.422/LE	

Abbreviation: LE, locally excited state.

On note the γ* value did not significantly change upon relaxation (max change below 0.05 Bohr^−1^), allowing the use of the vertical range separation split parameter reported in Table 2 also for the description of both the triplet and singlet relaxed ES.

**FIGURE 2:**
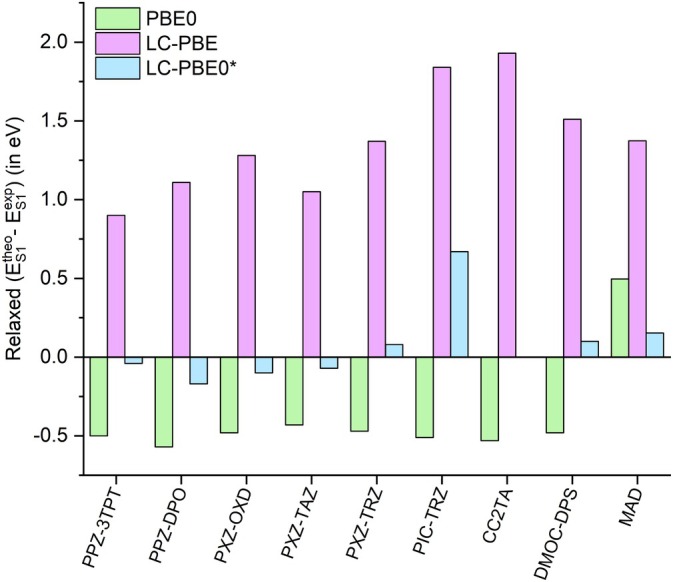
Difference (in eV) between computed and experimental relaxed *S*_1_ obtained using different levels of theory and the 6‐311G(d) basis set. Refer to Scheme 1 for molecular systems labelling. MAD represents the mean absolute deviation with respect to the experimental values computed on the full set of molecules.

Let us first concentrate on the predicted relaxed singlet energies. From the comparison of the computed and experimental energies (Table 3 and Figure 2), it is easy to note both from raw data and from computed deviation that the underestimation of the singlet energies at the PBE0 level already pointed out for vertical transitions is still present upon relaxation while the overestimation of the energies at the LC‐PBE level is still extremely high (up to 1.93 eV). On the other hand, for all molecules but PIC‐TRZ, LC‐PBE0* improves the prediction not only with respect to LC‐PBE but also with respect to PBE0, with acceptable deviation with respect to the experimental data. Indeed, the MAD decreases from 1.37 to 0.50 eV to 0.15 eV going from LC‐PBE to PBE0 to LC‐PBE0*. The reason for the larger error associated with the PIC‐TRZ system is the same as explained above for vertical excitations. Furthermore, the LC‐PBE0* predicts (in agreement with the experimental data) all these states to possess a marked CT character, thus allowing for correcting the wrong energetics of PBE0 without impacting the prediction of the nature of the first ES, as is the case of LC‐PBE that predicts all singlet states to be of LE character except for PXZ‐TRZ and CC2TA.

Considering the triplet state, it is interesting to note how there is a marked difference in the prediction of the character of these states, comparing PBE0 and the range‐separated hybrids. PBE0 predicts the lowest energy triplet of all molecules to be of CT nature. On the opposite side, LC‐PBE predicts all lowest energy triplets to possess a LE character. Interestingly, and in full agreement with experimental spectroscopic data, only LC‐PBE0* is able to correctly recover that while some of the triplets are indeed proven to be of LE type (such as PPZ‐3TPT PIC‐TRZ, or CC2TA for instance), others (such as PXZ‐TRZ) are still of CT character. For the conception of novel TADF molecules, the ability to reproduce not only the energetic but also the character of the ES is clearly a very positive point, making the difference between the global hybrid and our novel adaptable RSH.

Finally, in Table 4, we collect computed vertical and adiabatic singlet–triplet gaps together with the available experimental data.

**TABLE 4:** Experimental and computed singlet (*S*_1_)–triplet (*T*_1_) energy gap (in eV). Positive energies indicate that the first triplet (*T*_1_) is more stable than the first excited singlet state (*S*_1_).

	${∆E}_{\mathrm{ST}}^{\mathrm{ex}p}$	PBE0		LC‐PBE		LC‐PBE0*	
		${∆E}_{\mathrm{ST}}^{V}$	${∆E}_{\mathrm{ST}}^{\text{adia}}$	${∆E}_{\mathrm{ST}}^{V}$	${∆E}_{\mathrm{ST}}^{\text{adia}}$	${∆E}_{\mathrm{ST}}^{V}$	${∆E}_{\mathrm{ST}}^{\text{adia}}$
PPZ‐3TPT	0.27	0.01	0.01	0.98	0.92	0.19	0.17
PPZ‐DPO	0.09	0.01	0.01	0.97	0.92	0.01	0.02
PXZ‐OXD	/	0.01	0.01	1.11	1.12	0.17	0.16
PXZ‐TAZ	/	0.01	0.05	1.19	1.03	0.22	0.22
PXZ‐TRZ	0.06	0.01	0.01	1.00	0.92	0.16	0.10
PIC‐TRZ	0.18	0.10	0.03	1.19	1.47	0.75	0.68
CC2TA	0.20	0.08	0.06	1.23	1.44	0.39	0.13
DMOC‐DPS	0.24	0.29	0.01	1.17	1.31	0.74	0.53
MAD		0.107	0.152	0.917	0.990	0.253	0.178

First of all, we can note that all methods recover the fact that triplets are always more stable than the singlet. The PBE0 functional always predicts very small singlet‐triplet energy gaps, especially when considering adiabatic (relaxed) energies. The very small MAD with respect to the experimental values actually increases going from vertical to adiabatic energies, showing that the good agreement is actually also resulting from error compensation and a significant and coherent underestimate of both singlet and triplet energies. Considering LC‐PBE, there is a significant (and systematic) overestimation of the gaps. Relaxation only very slightly affects all molecules, with the exception of PIC‐TRZ, CC2TA, and DMOC‐DPS, for which the already large gap is further increased, negatively impacting the MAD. Finally, for the LC‐PBE0* functional, with the exception of the above‐mentioned PIC‐TRZ molecule, the computed singlet‐triplet gaps are close to the experimental ones and of state‐of‐the‐art computational approaches. Interestingly, the consideration of structural relaxation going from vertical to adiabatic energies actually improves the description of the energy gap with a MAD reducing from 0.25 to 0.18 eV. Interestingly, the LC‐PBE0* is also able in most of the cases to make a difference between systems experimentally showing a very small gap (i.e., below 0.1 eV, such as PPZ‐DPO) and the other (showing a gap close to or larger than 0.2 eV). Previous studies [64] have pointed out that the relaxation energy for a given ES (defined as the difference between its vertical and adiabatic energy) may be correlated with the local nature of the state. Unfortunately, due to the very different nature of the systems analyzed, no significant correlation can be pointed out in our study between the ES local nature (measured by the D_CT_) and its relaxation. The only qualitative and general result in this respect is that the computed triplet relaxation at LC‐PBE level is very high when compared to what is predicted with all other approaches.

Overall, the LC‐PBE0* approach produces the most balanced description of the singlet and triplet energy and of their relaxation, not only reducing the MAD of gaps to 0.178 eV but also providing a reliable estimate of the singlet and triplet ES absolute energy and nature.

## 4 Conclusions

In this work, we have shown how the recently developed adaptable RSHs can be used to compute emission from singlet and triplet states. It is worth mentioning here that we have essentially focused on recovering a correct description of the exchange contribution in our functional, but recent work has also shown the importance of correctly considering correlation effects for an accurate description of TADF systems [68].

Indeed, comparing the behavior of the LC‐PBE0* functional with that of the corresponding global (PBE0) and range‐separated hybrid (LC‐PBE), this functional provides both the energy of vertical and relaxed ES in better agreement with the experimental values, correcting the underestimation/overestimation of its global/range‐separated hybrid analogues. It is also interesting to stress that the tuning procedure enables recovery of the nature (CT or LE) of the emissive states, including the triplet.

From the computational point of view, our procedure is not computationally heavy since it only adds a vertical TD‐DFT calculation at global hybrid level to define the initial tuned range separation parameter and to check its validity every five steps during the optimization process. Of note for the systems studied the range separation parameter was not significantly changing during the optimization process, allowing the use of the one computed vertically (i.e., at the GS optimized geometry).

Overall, besides the case of symmetric systems for which the underlying definition of the CT distance is not applicable, the adaptable RSH appears to be a significant improvement for the description of singlet and triplet ES relaxation and thus for the design of novel TADF systems.

## Conflicts of Interest

The authors declare no conflicts of interest.

## Supporting information


**Data S1:** jcc70275‐sup‐0001‐Supinfo.doc.

## Data Availability

The data that support the findings of this study are available in the Supporting Information of this article.
