# Supplementary material for: Emission Using Adaptable Range Separated Hybrids: Thermally Activated Delayed Fluorescence Emitters as Test Case
Source: J Comput Chem. 2025 Nov 21;46(31):e70275. doi: 10.1002/jcc.70275 (PMC12637433; doi:10.1002/jcc.70275)
Supplement: Supplementary file 1 — Data S1: jcc70275‐sup‐0001‐Supinfo.doc. [file JCC-46-0-s001.doc]

**Emission via *Adaptable* Range Separated Hybrids: Thermally Activated Delayed Fluorescence emitters as test case**

*Tianhong Yan, Carlo Adamo* and Ilaria Ciofini**

Supporting Information

**Table S.1** Vertical energies (E, eV), oscillator strengths (f, a.u.), charge transfer distances (D_CT_, Å) and ghost-hunter indexes (M_AC_, eV) computed for TADF systems (Scheme 1) using different levels of theory and the 6-311G(d) basis set. When relevant the range separation parameter (γ, bohr^-1^) is reported. The value used to evaluate the range separation parameter for LC-PBE0* is reported in bold.

|  | CC2TA | | | | |
| --- | --- | --- | --- | --- | --- |
|  | E/f/D_CT_/M_AC_ | | | | |
|  | PBE0 | | LC-PBE | | LC-PBE0* |
| ES1 | 3.16/0.05/7.760/6.82 | | 4.74/0.81/0.514/-18.00 | | 3.75/0.35/4.399/5.69 |
| ES2 | 3.19/0.05/8.664/7.02 | | 4.77/0.04/0.577/-14.60 | | 3.83/0.11/4.553/5.71 |
| ES3 | 3.57/0.19/4.184/5.80 | | 4.78/0.14/0.106/-125.64 | | 3.99/0.11/3.807/5.61 |
| ES4 | 3.61/0.04/4.260/5.85 | | 4.87/0.09/0.671/-11.25 | | 4.04/0.02/3.916/5.76 |
| ES5 | 3.67/0.00/9.440/7.45 | | 5.03/0.38/1.100/-2.98 | | 4.12/0.27/2.289/3.30 |
| ES6 | 3.70/0.00/**10.510**/7.61 | | 5.15/0.30/0.987/-4.37 | | 4.17/0.02/1.253/-1.63 |
| ES7 | 3.78/0.01/5.622/6.94 | | 5.18/0.05/0.059/-233.51 | | 4.17/0.07/0.325/-34.45 |
| ES8 | 3.80/0.03/4.193/6.11 | | 5.19/0.14/0.424/-23.46 | | 4.19/0.12/1.854/1.87 |
| ES9 | 3.80/0.14/2.056/2.55 | | 5.20/0.17/0.307/-36.38 | | 4.26/0.09/3.733/5.64 |
| ES10 | 3.83/0.05/4.118/6.00 | | 5.22/0.38/0.413/-24.25 | | 4.32/0.13/3.271/5.13 |
| γ | -- | | 0.470 | | 0.101 |
|  | DMOC-DPS | | | | |
|  | E/f/D_CT_/M_AC_ | | | | |
|  | PBE0 | | LC-PBE | | LC-PBE0* |
| ES1 | 3.21/0.41/3.848/5.21 | | 4.38/0.22/0.259/-45.79 | | 3.81/0.73/2.597/3.72 |
| ES2 | 3.36/0.11/3.853/5.31 | | 4.46/0.23/0.147/-88.16 | | 3.92/0.02/0.531/-17.65 |
| ES3 | 3.70/0.06/0.714/-10.81 | | 4.62/0.52/2.306/3.52 | | 3.98/0.10/1.174/-2.77 |
| ES4 | 3.77/0.05/0.562/-16.16 | | 4.84/0.21/2.358/3.61 | | 4.04/0.10/2.883/4.43 |
| ES5 | 3.79/0.00/6.130/7.13 | | 4.92/0.27/0.106/-125.64 | | 4.38/0.00/3.445/5.90 |
| ES6 | 3.82/0.00/4.357/6.54 | | 4.97/0.34/0.189/-65.89 | | 4.46/0.01/3.400/5.91 |
| ES7 | 3.88/0.00/6.137/7.24 | | 5.23/0.00/2.149/4.45 | | 4.55/0.20/0.678/-11.33 |
| ES8 | 3.90/0.00/4.339/6.66 | | 5.31/0.00/2.140/4.57 | | 4.60/0.26/0.218/-56.01 |
| ES9 | 4.11/0.05/**7.522**/7.96 | | 5.47/0.05/1.545/1.70 | | 4.63/0.03/4.731/6.76 |
| ES10 | 4.19/0.03/2.200/3.22 | | 5.53/0.04/2.345/4.89 | | 4.72/0.01/4.910/7.00 |
| γ | -- | | 0.470 | | 0.141 |
|  | PIC-TRZ | | | | |
|  | E/f/D_CT_/M_AC_ | | | | |
|  | PBE0 | LC-PBE | | LC-PBE0* | |
| ES1 | 3.02/0.02/6.616/6.13 | 4.45/0.02/0.453/-22.27 | | 3.90/0.04/2.177/2.50 | |
| ES2 | 3.10/0.01/**6.653**/6.23 | 4.47/0.02/0.137/-95.54 | | 3.93/0.02/2.508/3.22 | |
| ES3 | 3.29/0.06/6.218/6.30 | 4.65/0.18/1.132/-3.19 | | 4.08/0.10/2.321/2.99 | |
| ES4 | 3.33/0.07/6.260/6.29 | 4.76/0.02/1.003/-4.63 | | 4.17/0.40/4.169/5.45 | |
| ES5 | 3.48/0.02/3.365/4.76 | 4.77/0.15/0.455/-21.90 | | 4.20/0.05/4.201/5.72 | |
| ES6 | 3.59/0.01/3.446/4.95 | 4.90/2.65/0.129/-101.76 | | 4.24/0.25/2.995/4.44 | |
| ES7 | 3.78/0.13/2.694/3.97 | 5.07/0.06/0.177/-71.38 | | 4.27/0.02/3.935/5.44 | |
| ES8 | 3.80/0.03/2.832/4.22 | 5.13/0.34/2.173/3.12 | | 4.41/0.88/1.618/0.55 | |
| ES9 | 3.82/0.00/3.311/4.99 | 5.20/0.09/2.952/5.04 | | 4.41/0.03/4.318/5.91 | |
| ES10 | 3.93/1.26/1.517/-0.05 | 5.20/0.00/1.928/2.56 | | 4.48/0.88/2.898/4.40 | |
| γ | -- | 0.470 | | 0.159 | |
|  | PXZ-TRZ | | | | |
|  | E/f/D_CT_/M_AC_ | | | | |
|  | PBE0 | | LC-PBE | | LC-PBE0* |
| ES1 | 2.29/0.00/6.377/5.95 | | 4.31/0.00/4.292/5.53 | | 2.93/0.11/5.369/5.68 |
| ES2 | 2.62/0.00/8.956/6.63 | | 4.36/0.02/0.183/-68.38 | | 3.63/0.00/7.109/6.75 |
| ES3 | 3.53/0.00/3.113/5.24 | | 4.85/0.17/0.543/-15.66 | | 3.83/0.01/2.830/4.68 |
| ES4 | 3.75/0.00/8.041/7.99 | | 4.97/0.00/0.024/-586.59 | | 4.12/0.07/4.206/6.58 |
| ES5 | 3.86/0.05/2.119/3.56 | | 5.01/0.05/0.250/-46.46 | | 4.33/0.00/0.146/-85.86 |
| ES6 | 3.90/0.00/6.981/7.81 | | 5.06/0.00/0.458/-18.04 | | 4.39/0.00/1.305/1.31 |
| ES7 | 4.14/0.00/**9.240**/8.39 | | 5.06/0.00/0.667/-8.23 | | 4.40/0.00/0.016/-887.75 |
| ES8 | 4.21/0.00/0.307/-34.34 | | 5.14/0.00/1.096/0.26 | | 4.42/0.00/1.749/2.51 |
| ES9 | 4.23/0.00/1.663/2.43 | | 5.32/0.00/0.358/-28.88 | | 4.47/0.01/1.466/3.27 |
| ES10 | 4.23/0.00/2.410/4.38 | | 5.37/0.54/1.127/-1.82 | | 4.51/0.09/2.054/3.72 |
| γ | -- | | 0.470 | | 0.115 |

**Figure S.1** Isocontour representation (isovalue 0.05 e.u.) of the NTOs (hole: left; electron: right) associated to first excited state of CC2TA

| ES1 PBE0 |  |
| --- | --- |
| 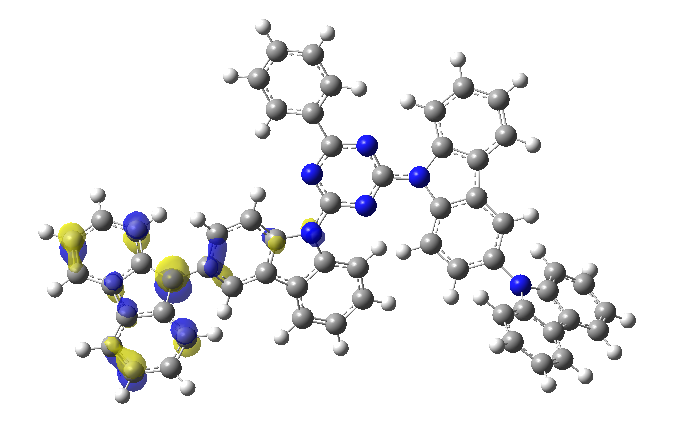 | 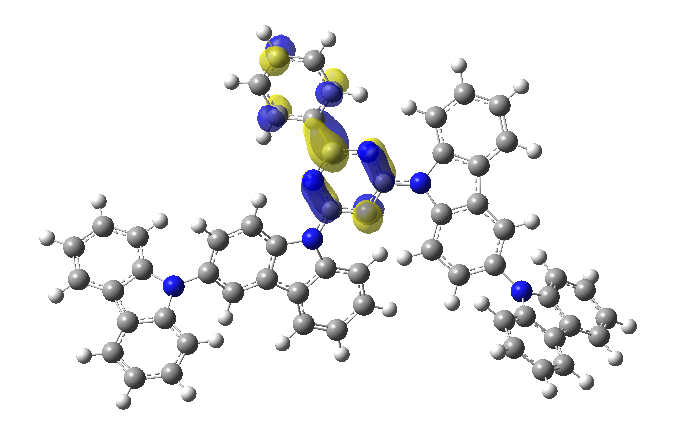 |
| ES1 LC-PBE |  |
| 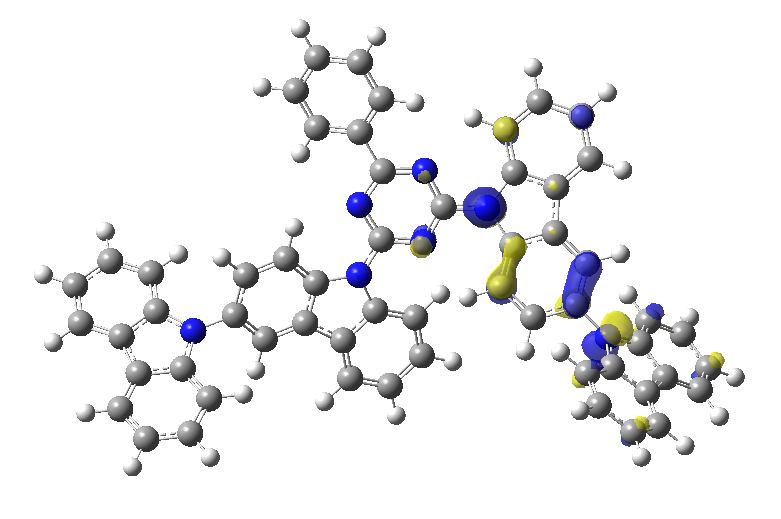 | 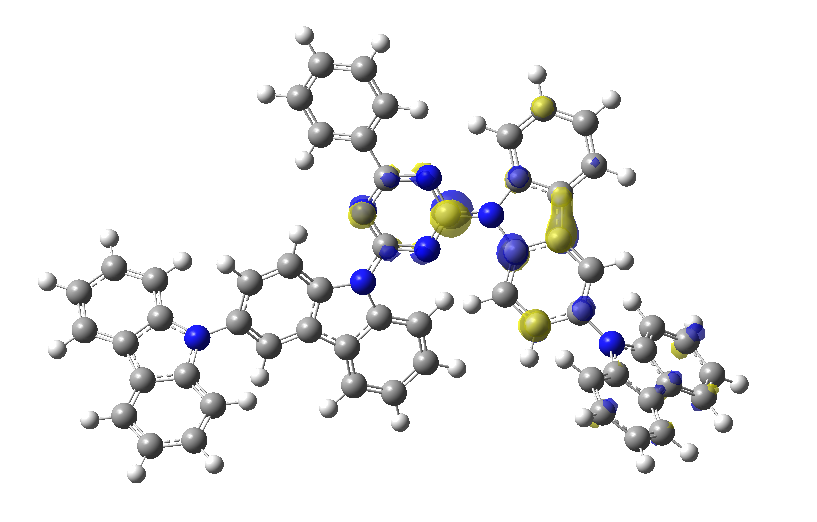 |
| LC-PBE0* |  |
| 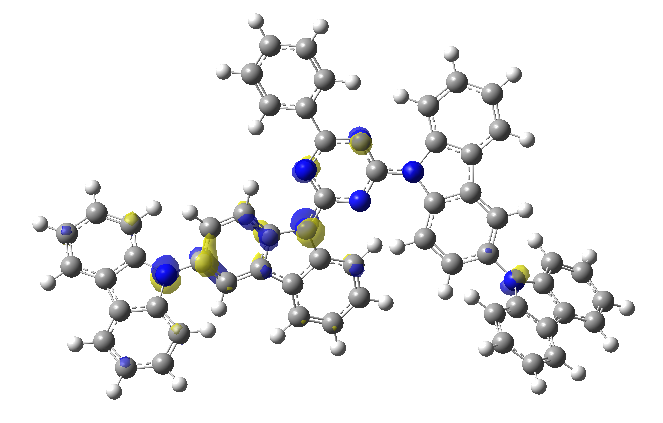 | 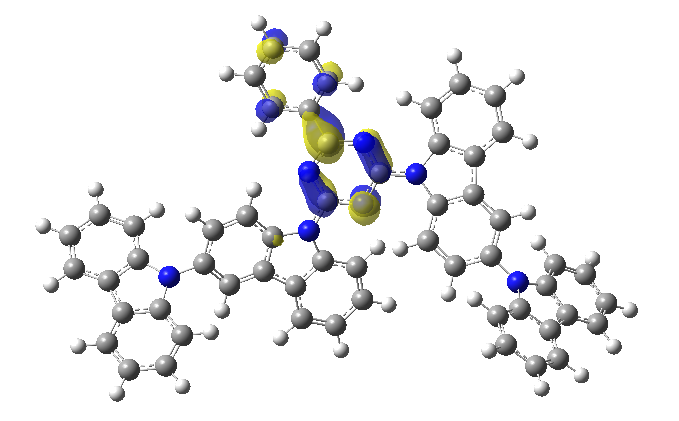 |

**Figure S.2** Isocontour representation (isovalue 0.05 e.u.) of the NTOs (hole: left; electron: right) associated to first excited state of DMOC-DPS

| ES1 PBE0 |  |
| --- | --- |
| 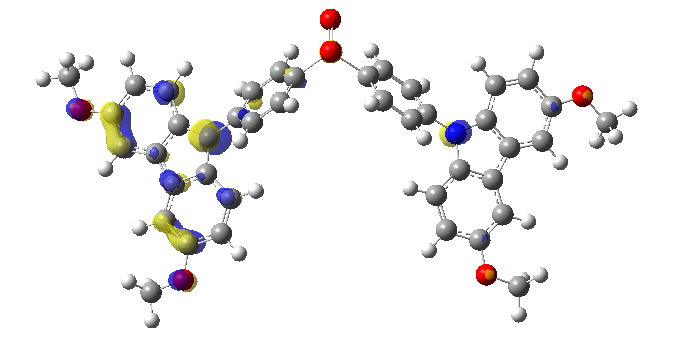 | 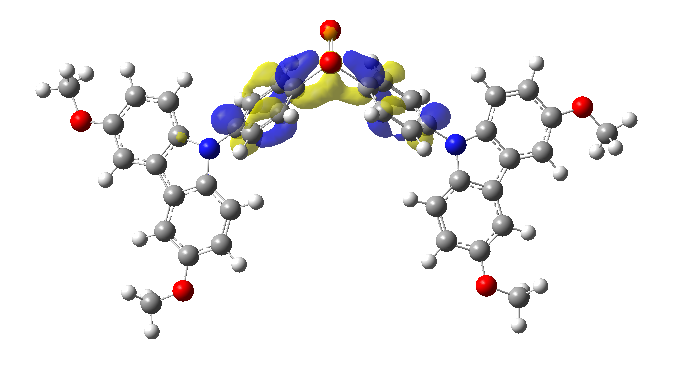 |
| ES1 LC-PBE |  |
| 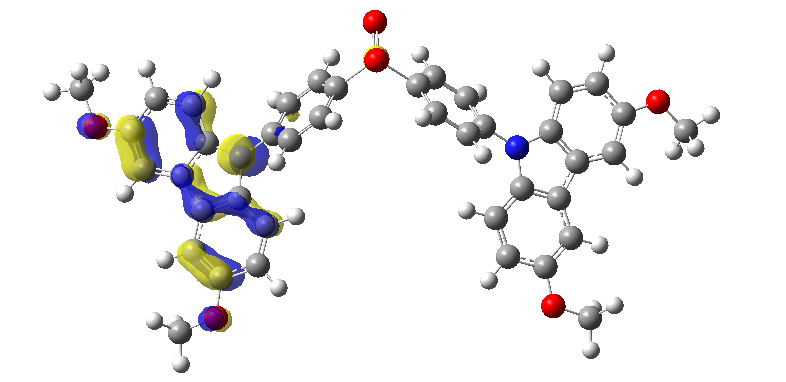 | 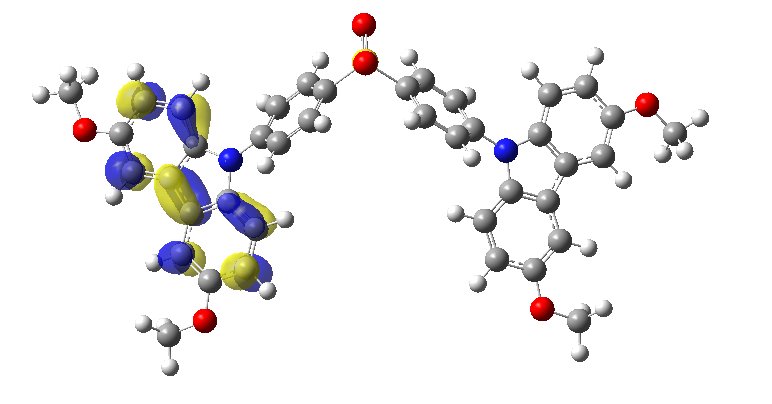 |
| LC-PBE0* |  |
| 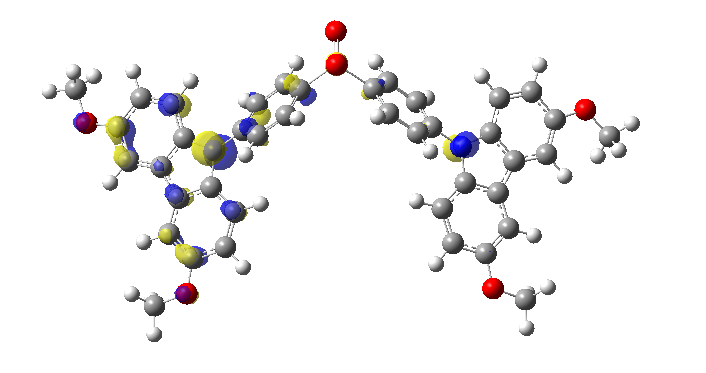 | 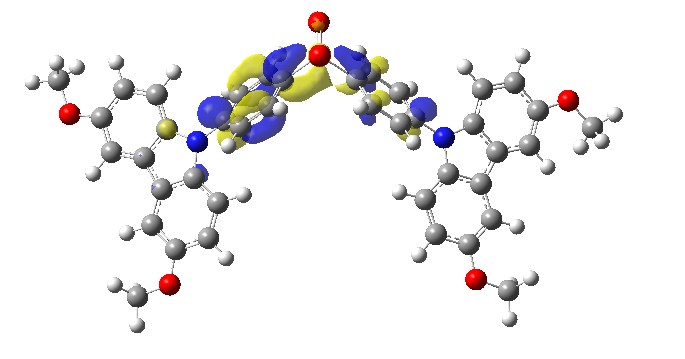 |

**Figure S.3** Isocontour representation (isovalue 0.05 e.u.) of the NTOs (hole: left; electron: right) associated to first excited state of PIC-TRZ

| ES1 PBE0 |  |
| --- | --- |
| 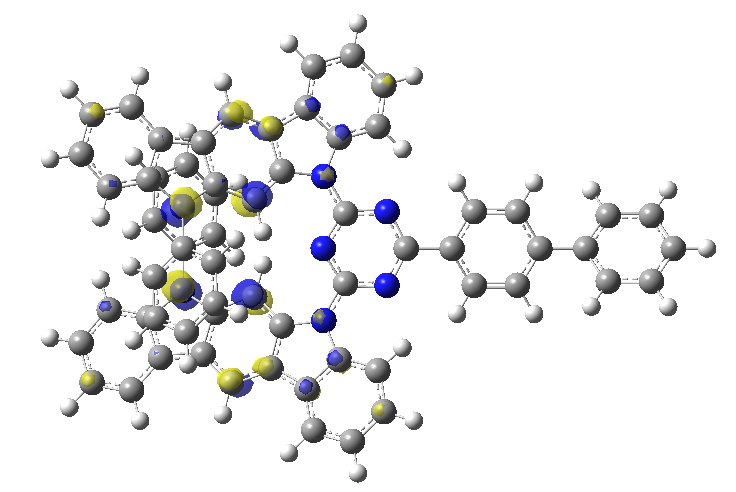 | 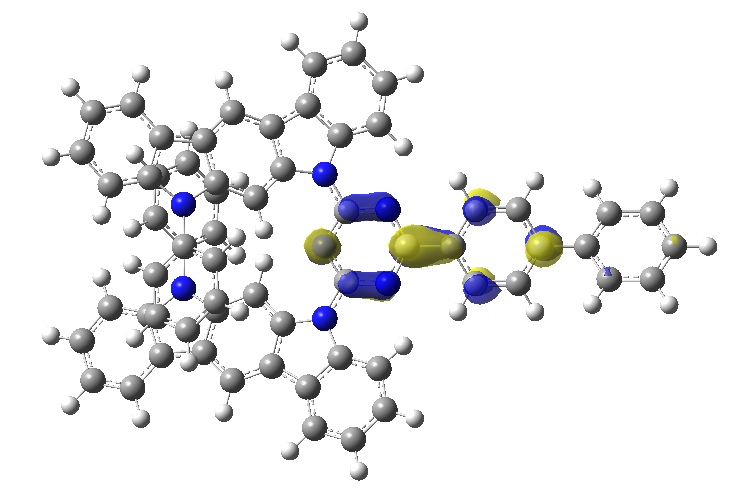 |
| ES1 LC-PBE |  |
| 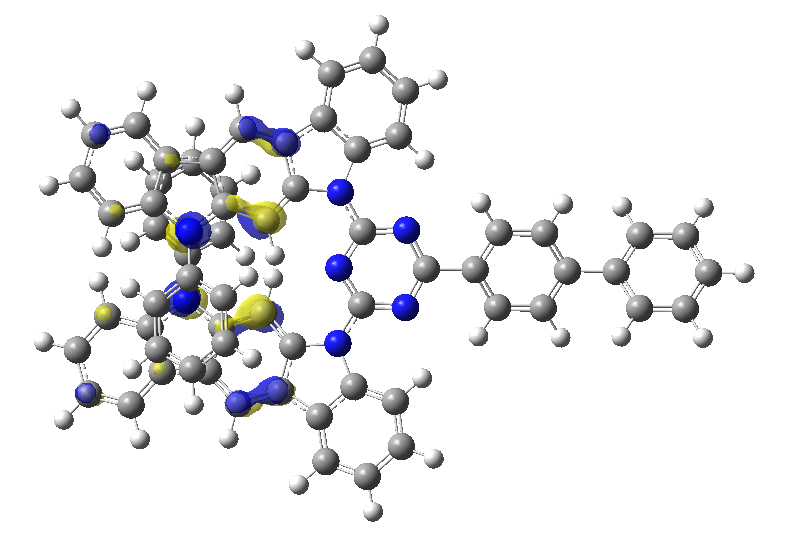 | 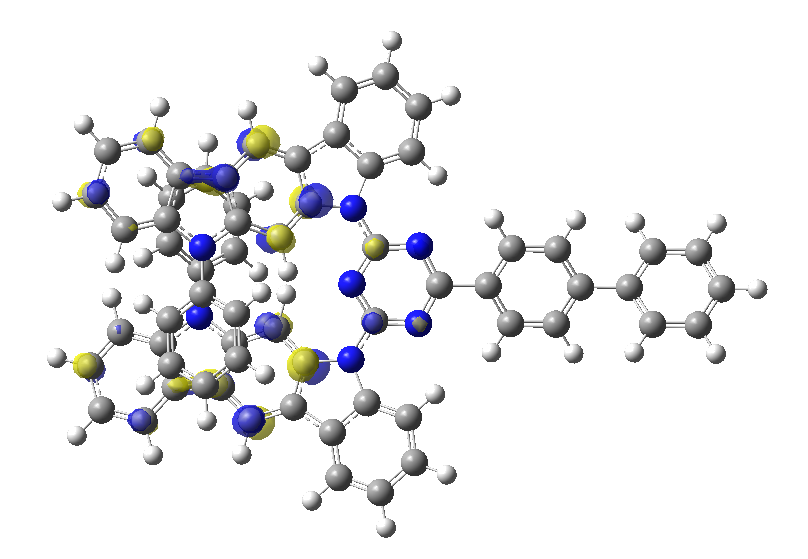 |
| LC-PBE0* |  |
| 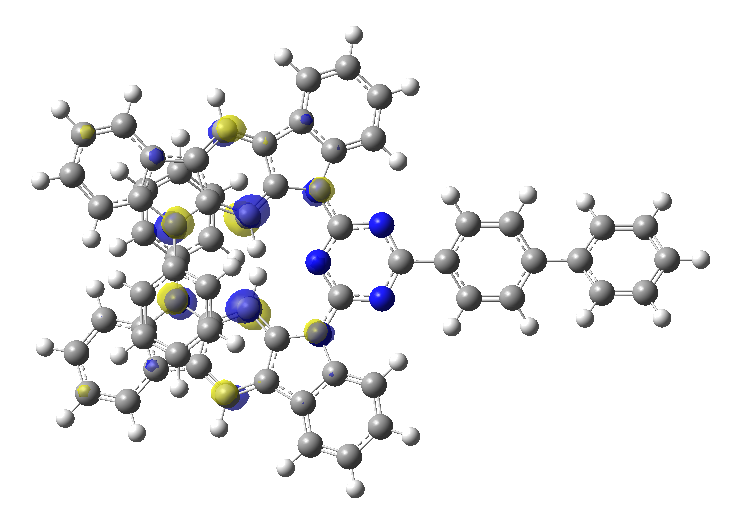 | 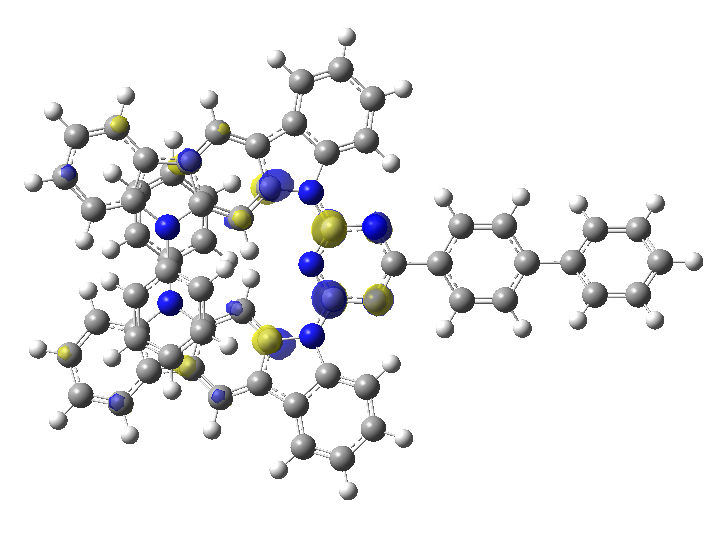 |

**Figure S.4** Isocontour representation (isovalue 0.05 e.u.) of the NTOs (hole: left; electron: right) associated to first excited state of PXZ-TRZ

| ES1 PBE0 |  |
| --- | --- |
| 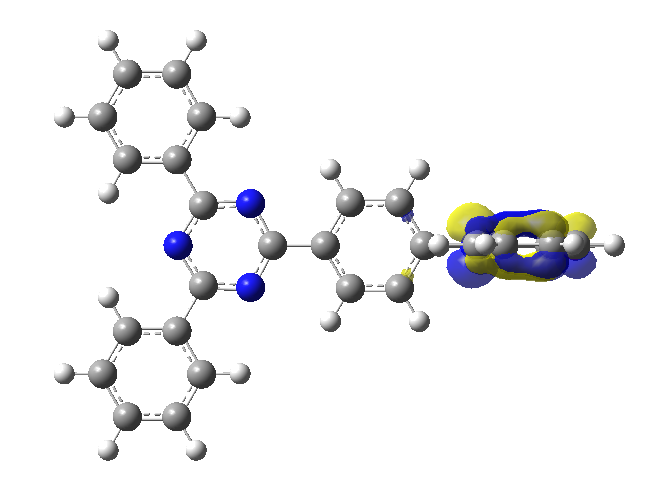 | 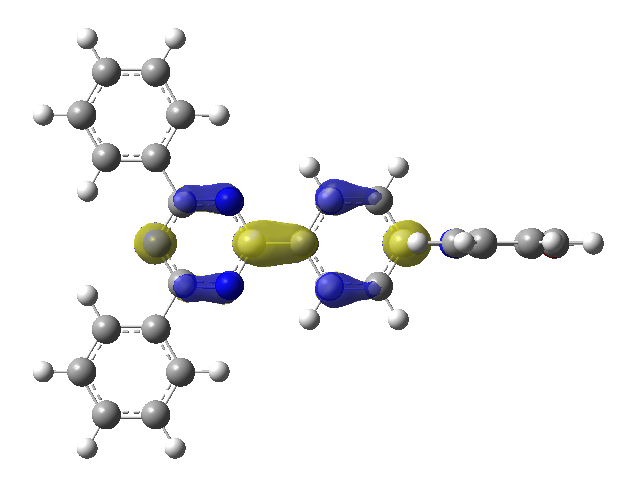 |
| ES1 LC-PBE |  |
| 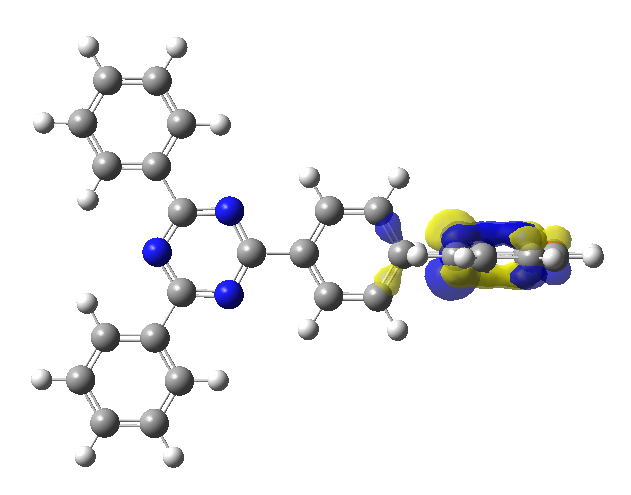 | 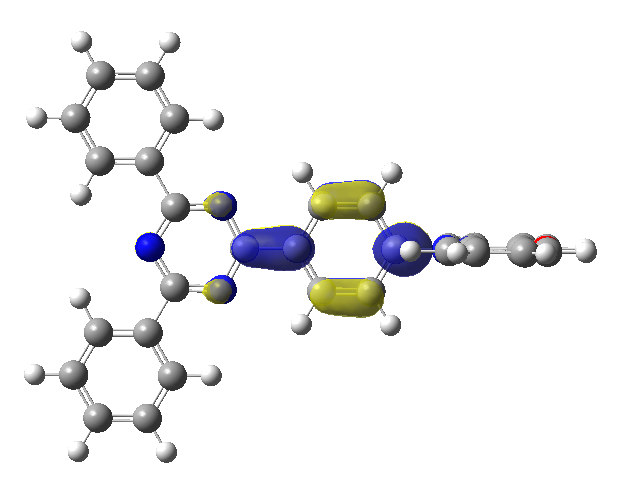 |
| LC-PBE0* |  |
| 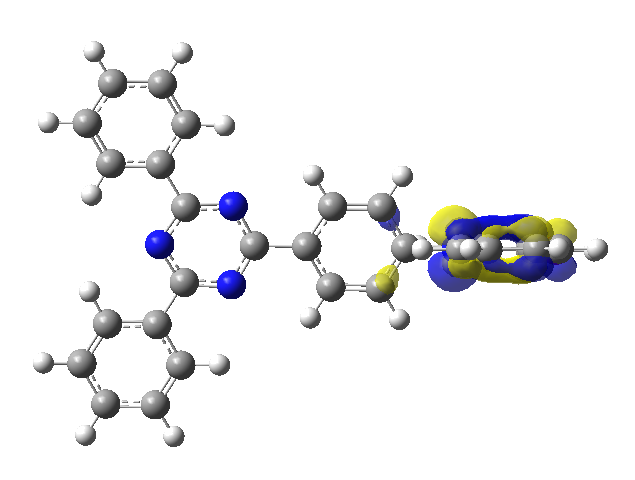 | 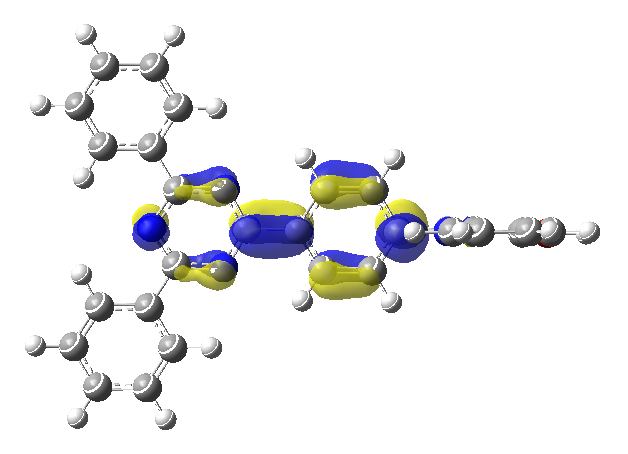 |

**Table S.2** Effect of basis set and solvent (toluene) on vertical singlet and triplet energies of PPZ-3TPT computed at TD level using different DFA.

|  | PBE0 | | | LC-PBE | | | LC-PBE0* | | |
| --- | --- | --- | --- | --- | --- | --- | --- | --- | --- |
|  | 6-311G(d)  vacuum | 6-311g(d)  Toluene | 6-31+G(d) | 6-311G(d)  vacuum | 6-311g(d)  Toluene | 6-31+G(d) | 6-311G(d)  vacuum | 6-311g(d)  Toluene | 6-31+G(d) |
| E(S_1_) | 2.51 | 2.59 | 2.56 | 3.94 | 3.84 | 3.99 | 3.00 | 3.09 | 3.00 |
| D_CT_(S_1_) | 5.8 | 5.7 | 5.7 | 0.2 | 0.1 | 0.2 | 4.8 | 4.7 | 4.8 |
| E(T_0_) | 2.44 | 2.43 | 2.48 | 2.44 | 2.39 | 2.45 | 2.61 | 2.54 | 2.64 |
| D_CT_(T_0_) | LE | LE | LE | LE | LE | LE | LE | LE | LE |
| ΔE_ST_ | 0.07 | 0.16 | 0.08 | 1.50 | 1.45 | 1.54 | 0.39 | 0.55 | 0.36 |

**Table S.3**. Excited State energies (E, eV), oscillator strengths (f, a.u.), charge transfer distances (D_CT_, Å) and M_AC_ index (in eV) computed for the first singlet (S_1_) and triplet (T_1_) excited states optimized at TD level using different levels of theory and the 6-311G(d) basis set. The range separation parameter (γ^*^, Bohr^-1^) used for each system at LC-PBE0* is reported. For LC-PBE calculations, the standard γ value (γ=0.470 Bohr^-1^) is used.

|  | PBE0 | | LC-PBE | | LC-PBE0* | | |
| --- | --- | --- | --- | --- | --- | --- | --- |
|  | E (S_1_)/  f (S_1_) | D_CT_ (S_1_)/  M_AC_ (S_1_) | E(S_1_)/  f (S_1_) | D_CT_ (S_1_)/  M_AC_ (S_1_) | E (S_1_)/  f (S_1_) | D_CT_ (S_1_)/  M_AC_ (S_1_) | *γ* * |
| **PPZ-3TPT** | 1.84 | 6.258/5.02 | 3.11 | 0.044/LE | 2.30 | 5.080/4.76 | 0.105 |
| **PPZ-DPO** | 1.58 | 6.208/4.78 | 3.12 | 0.038/LE | 1.98 | 5.332/4.56 | 0.095 |
| **PXZ-OXD** | 2.02 | 5.893/5.33 | 3.62 | 0.085/LE | 2.40 | 5.110/5.12 | 0.094 |
| **PXZ-TAZ** | 2.29 | 5.874/5.56 | 3.61 | 0.123/LE | 2.64 | 4.715/5.16 | 0.107 |
| **PXZ-TRZ** | 1.80 | 6.152/5.21 | 3.56 | 3.866/4.58 | 2.35 | 5.311/4.99 | 0.115 |
| **PIC-TRZ** | 2.00 | 7.121/4.99 | 4.18 | 0.708/LE | 3.19 | 5.592/4.96 | 0.159 |
| **CC2TA** | 2.09 | 7.224/5.38 | 4.36 | 2.009/2.23 | 2.62 | 6.073/5.11 | 0.101 |
| **DMOC-DPS** | 2.31 | 5.550/5.59 | 4.05 | 0.448/LE | 2.88 | 4.523/5.08 | 0.141 |
|  | PBE0 | | LC-PBE | | LC-PBE0* | | |
|  | E (T_1_)/  f (T_1_) | D_CT_(T_1_)/  M_AC_ (T_1_) | E(T_1_)/  f (T_1_) | D_CT_ (T_1_)/  M_AC_ (T_1_) | E (T_1_)/  f (T_1_.) | D_CT_ (T_1_)/  M_AC_ (T_1_) | *γ** |
| **PPZ-3TPT** | 1.84 | 6.213/5.13 | 1.52 | 0.085/LE | 1.95 | 0.048/LE | 0.105 |
| **PPZ-DPO** | 1.57 | 6.177/4.77 | 1.54 | 0.002/LE | 1.85 | 4.042/4.12 | 0.095 |
| **PXZ-OXD** | 1.80 | 3.025/3.25 | 1.90 | 0.076/LE | 1.78 | 1.212/LE | 0.094 |
| **PXZ-TAZ** | 2.21 | 0.063/LE | -1.22 | 0.142/LE | 2.27 | 0.066/LE | 0.107 |
| **PXZ-TRZ** | 1.79 | 6.128/5.20 | 1.89 | 0.074/LE | 2.11 | 3.705/4.47 | 0.115 |
| **PIC-TRZ** | 2.00 | 6.978/5.02 | -0.93 | 0.292/LE | 2.29 | 0.497/LE | 0.159 |
| **CC2TA** | 2.04 | 6.894/5.34 | -0.87 | 0.364/LE | 2.41 | 0.735/LE | 0.101 |
| **DMOC-DPS** | 2.42 | 4.532/5.46 | -1.35 | 0.417/LE | 2.37 | 0.420/LE | 0.141 |

CC2TA_S0_PBE0

C 1.15587000 2.38156800 -0.04368800

N 0.56953700 3.52525700 0.29983100

C -0.76267400 3.50604700 0.26809500

N -1.50019800 2.45088200 -0.07781000

C -0.80622400 1.36545000 -0.41021400

N 0.52105800 1.27084800 -0.41034200

C -1.47257800 4.74229000 0.64472100

C -0.78627300 5.78402900 1.27386200

C -1.45691200 6.94345800 1.63325100

C -2.81427400 7.07755600 1.36003600

C -3.50166900 6.04567400 0.72934000

C -2.83674100 4.88088800 0.37679200

N -1.52255300 0.24631100 -0.78791200

N 2.53617500 2.34154000 -0.01131800

C 3.32902900 1.17720300 0.05369300

C 4.68234900 1.56042500 0.07010600

C 4.72595000 3.00070700 0.00488500

C 3.39775200 3.46114000 -0.03449100

C -2.87665500 -0.00570500 -0.48446900

C -3.22609500 -1.25915400 -1.01824000

C -2.06240400 -1.78545500 -1.68857800

C -1.02607800 -0.84791500 -1.53070100

C 2.97145200 -0.16437900 0.15685000

C 3.98548300 -1.10709000 0.24108200

C 5.33476100 -0.73527300 0.24919700

C 5.68732200 0.60582000 0.17411400

C 5.78688100 3.90093800 -0.03787300

C 5.51246400 5.25519000 -0.12963500

C 4.19053700 5.70128700 -0.18556700

C 3.11754400 4.82084700 -0.13978900

C -1.85075300 -2.95607400 -2.41147600

C -0.60750800 -3.17629600 -2.98072800

C 0.40819900 -2.22909300 -2.83738400

C 0.21907600 -1.05630300 -2.11764500

C -3.79872200 0.73308800 0.25202300

C -5.06638600 0.20069200 0.43425600

C -5.43084000 -1.03334500 -0.11620200

C -4.50847900 -1.76843100 -0.84855100

N 6.33714100 -1.72698200 0.33776700

N -6.73770400 -1.53315200 0.08385000

C 6.46180100 -2.66531400 1.35435900

C 7.58036600 -3.48460800 1.08848000

C 8.15549900 -3.01399100 -0.14730000

C 7.36041400 -1.93010900 -0.57928000

C -7.91392100 -0.89900100 -0.29322400

C -9.01085900 -1.70686300 0.07784400

C -8.46523100 -2.88421200 0.70682100

C -7.06082300 -2.73916800 0.69211000

C 5.68918700 -2.83305200 2.50042200

C 6.04464500 -3.85068700 3.37283600

C 7.14299500 -4.68069700 3.11899700

C 7.91540900 -4.50032600 1.98305000

C 9.24579400 -3.41561300 -0.91801800

C 9.52321800 -2.74296500 -2.09711700

C 8.71682700 -1.67824700 -2.51628500

C 7.62683300 -1.25765100 -1.76903600

C -9.03158200 -4.02150200 1.28138500

C -8.20111800 -4.98490500 1.83071000

C -6.81115500 -4.81857400 1.81795600

C -6.22128700 -3.69790800 1.25291600

C -8.08958900 0.31190300 -0.95757600

C -9.38850700 0.71292800 -1.23164500

C -10.48860200 -0.06915100 -0.86016600

C -10.30632700 -1.27909400 -0.21014400

H 0.26957900 5.66434500 1.48885500

H -0.92018500 7.74525500 2.13041200

H -3.33718200 7.98720300 1.63922700

H -4.55965200 6.15056900 0.51077800

H -3.35982900 4.07294700 -0.12216000

H 1.93743100 -0.47397400 0.15195400

H 3.73086300 -2.16046100 0.29090800

H 6.73274200 0.89374600 0.21399500

H 6.81182600 3.54424900 -0.00586900

H 6.32590600 5.97266500 -0.16334500

H 3.98861100 6.76491100 -0.26564500

H 2.10031100 5.17947300 -0.17894500

H -2.65104700 -3.67981200 -2.53181200

H -0.42272900 -4.08360500 -3.54654300

H 1.37515800 -2.40684400 -3.29740600

H 1.01608400 -0.33518800 -2.01810600

H -3.53563700 1.68909200 0.67832600

H -5.79563800 0.74584700 1.02422500

H -4.79534100 -2.72199200 -1.27965800

H 4.84333600 -2.18708300 2.70956600

H 5.45802300 -4.00261100 4.27372700

H 7.39218300 -5.46864000 3.82252500

H 8.77286100 -5.13947000 1.79363000

H 9.86747000 -4.24743000 -0.59990400

H 10.36981900 -3.04492900 -2.70539000

H 8.94560400 -1.17034700 -3.44827100

H 7.00029400 -0.43874500 -2.10594000

H -10.11001300 -4.14882100 1.30115400

H -8.62939400 -5.87531600 2.27976900

H -6.17849000 -5.58079900 2.26248700

H -5.14406900 -3.57044800 1.25680500

H -7.24115100 0.91720300 -1.25827100

H -9.55296700 1.65299600 -1.74948400

H -11.49194600 0.27548500 -1.08919800

H -11.16136600 -1.88824500 0.06815200

CC2TA_S0_LC-PBE

C 1.14462800 2.36520800 -0.01611700

N 0.55413600 3.49882100 0.30342200

C -0.76245500 3.47919200 0.26364500

N -1.48528400 2.42920600 -0.07001000

C -0.79891900 1.34820300 -0.38087700

N 0.51502000 1.26334300 -0.36772800

C -1.47415800 4.71383300 0.61741600

C -0.80126300 5.74329200 1.24538400

C -1.46980700 6.89609100 1.58222600

C -2.80865400 7.02790200 1.28429800

C -3.48104500 6.00415700 0.65301600

C -2.81696800 4.84655700 0.32401300

N -1.50193600 0.23356800 -0.74343800

N 2.51021700 2.32805900 0.02357000

C 3.29838200 1.17189700 0.07590600

C 4.63383100 1.54973500 0.08353800

C 4.67595300 2.98684300 0.03035100

C 3.36479500 3.43996700 0.00492100

C -2.85198600 -0.01093900 -0.46209400

C -3.18157100 -1.26762300 -0.95026300

C -2.00484800 -1.81160000 -1.57416400

C -0.99073200 -0.87549200 -1.43244300

C 2.94359400 -0.15907000 0.17446700

C 3.94940000 -1.09107000 0.24012100

C 5.28503800 -0.72021100 0.23618000

C 5.63349200 0.60626600 0.16919600

C 5.72970000 3.87559100 -0.00785200

C 5.45917100 5.21748300 -0.08146900

C 4.14795000 5.66044900 -0.12302300

C 3.08580100 4.78967000 -0.08135300

C -1.78024200 -2.99782700 -2.24061400

C -0.53824700 -3.23207800 -2.77194300

C 0.46110600 -2.28176900 -2.64805300

C 0.25549300 -1.09563300 -1.98509900

C -3.78507500 0.74646200 0.21829300

C -5.04147800 0.22024200 0.38772700

C -5.38368400 -1.02312100 -0.12051900

C -4.45328600 -1.77357800 -0.79554000

N 6.28153500 -1.70597900 0.30694300

N -6.68462500 -1.51665000 0.06644300

C 6.41061200 -2.64904500 1.30179100

C 7.51167800 -3.45515300 1.02599800

C 8.07933400 -2.96998300 -0.20261500

C 7.29150900 -1.89625400 -0.60914400

C -7.84436500 -0.90180500 -0.34748000

C -8.93339700 -1.68884000 0.01749900

C -8.39996900 -2.84395800 0.68701600

C -7.01552900 -2.69567800 0.69541200

C 5.64582400 -2.82962500 2.43849400

C 5.99949100 -3.84794900 3.28609100

C 7.08776100 -4.66920300 3.02011800

C 7.84802600 -4.47515000 1.89599600

C 9.15819800 -3.35804500 -0.97438100

C 9.42849200 -2.67592300 -2.13247800

C 8.62549100 -1.61429100 -2.52925400

C 7.55000900 -1.20995500 -1.78043600

C -8.97235100 -3.95706500 1.27333600

C -8.15990900 -4.89427700 1.85724500

C -6.78122400 -4.72487300 1.86676400

C -6.18958800 -3.62970100 1.29129400

C -8.00502500 0.28301900 -1.04027000

C -9.28434700 0.67064500 -1.34636000

C -10.38281600 -0.09645200 -0.97958900

C -10.21316900 -1.27632100 -0.30238000

H 0.25112300 5.61859100 1.47740000

H -0.94202400 7.70052200 2.08417600

H -3.33500500 7.94012700 1.54773700

H -4.53431000 6.11037200 0.41436500

H -3.32793800 4.03227100 -0.17863900

H 1.90835600 -0.46707400 0.18447400

H 3.70105000 -2.14657600 0.28900300

H 6.68007300 0.89302200 0.20022700

H 6.75369300 3.51554800 0.01487900

H 6.27212000 5.93532000 -0.11238500

H 3.94729500 6.72509200 -0.18981400

H 2.06714300 5.14668700 -0.11122600

H -2.57672100 -3.72786900 -2.34723100

H -0.33826800 -4.15874700 -3.29954800

H 1.43601800 -2.47141300 -3.08585500

H 1.04487800 -0.36291100 -1.90364600

H -3.53155400 1.71848400 0.61443200

H -5.79066700 0.78014900 0.93855300

H -4.72840900 -2.74377700 -1.19748000

H 4.80195600 -2.18368100 2.65691600

H 5.41659200 -4.01265100 4.18697600

H 7.33816200 -5.46623500 3.71242000

H 8.70420200 -5.11154200 1.69283300

H 9.78089000 -4.19352500 -0.66854900

H 10.27237200 -2.96790600 -2.74880800

H 8.85124600 -1.09362400 -3.45473400

H 6.91927500 -0.38732400 -2.10082100

H -10.05070900 -4.08535700 1.27360700

H -8.59393400 -5.77393500 2.32099900

H -6.15666200 -5.47410000 2.34333800

H -5.11308000 -3.49596500 1.31204300

H -7.14915200 0.87910100 -1.33921600

H -9.44062900 1.59645800 -1.89136000

H -11.38116100 0.24123600 -1.23753200

H -11.07157300 -1.88025600 -0.02361300

CC2TA_S0_LC-PBE0*

C 1.14929400 2.37167600 -0.00770800

N 0.55742700 3.51890200 0.30079200

C -0.77020400 3.49087100 0.25698300

N -1.49968600 2.42869900 -0.06828900

C -0.79877800 1.34182900 -0.36751400

N 0.52526400 1.25217600 -0.35269400

C -1.48567200 4.72743900 0.59560500

C -0.80172800 5.79294100 1.17488300

C -1.47854700 6.95522600 1.49906400

C -2.83768100 7.06298400 1.23964500

C -3.52163900 6.00451600 0.65826200

C -2.84989600 4.83764800 0.34015100

N -1.50188800 0.21641200 -0.72145400

N 2.52141300 2.33768100 0.03734900

C 3.31351500 1.17828800 0.08256300

C 4.66195100 1.56349500 0.09091300

C 4.70031400 3.00192700 0.04757700

C 3.37410700 3.45692200 0.02564900

C -2.86159300 -0.01822100 -0.45472200

C -3.18851600 -1.29940900 -0.92192500

C -2.00200500 -1.86041900 -1.51334800

C -0.97800600 -0.91227600 -1.37767200

C 2.95820200 -0.16105700 0.17631700

C 3.97307000 -1.09970800 0.23729500

C 5.31777100 -0.72471100 0.23437600

C 5.66833100 0.61426200 0.17289300

C 5.75563100 3.90257900 0.01444800

C 5.47550900 5.25416500 -0.04948600

C 4.15483200 5.69530300 -0.08654000

C 3.08682100 4.81355500 -0.04995800

C -1.76318200 -3.06952100 -2.15142200

C -0.50212600 -3.31688100 -2.65971200

C 0.50133700 -2.35790800 -2.54322600

C 0.28427000 -1.14572400 -1.90786900

C -3.80895000 0.76364200 0.19338900

C -5.07955800 0.24093700 0.35684900

C -5.41960200 -1.02279800 -0.12879900

C -4.47246200 -1.80007300 -0.77471300

N 6.31660800 -1.71000900 0.30027900

N -6.72613100 -1.50838100 0.04834700

C 6.39332300 -2.72044700 1.24335000

C 7.52975600 -3.50744300 0.97936700

C 8.16722000 -2.93849300 -0.17846700

C 7.38820800 -1.83257900 -0.56717900

C -7.88782700 -0.84979200 -0.31425400

C -8.99353400 -1.65292200 0.02239200

C -8.46720600 -2.85433000 0.61411300

C -7.06561800 -2.72575000 0.61254100

C 5.56363200 -2.97676800 2.32727700

C 5.88153000 -4.05346100 3.13542700

C 6.99637700 -4.85449200 2.87939100

C 7.82607600 -4.58415300 1.80748000

C 9.29959400 -3.27204900 -0.91270900

C 9.63297900 -2.50928200 -2.01620900

C 8.84032100 -1.42423900 -2.39616700

C 7.70924500 -1.06997400 -1.68275300

C -9.05048000 -3.99822100 1.14717500

C -8.23653900 -4.98516400 1.67059000

C -6.84825100 -4.83413100 1.67421500

C -6.24207300 -3.70691000 1.14934900

C -8.04238800 0.37892200 -0.94305900

C -9.33161400 0.80161600 -1.21278200

C -10.44094700 0.02485400 -0.87185500

C -10.27863200 -1.20364200 -0.25943200

H 0.25887900 5.68556500 1.37918900

H -0.94528700 7.78136100 1.95879300

H -3.36696800 7.97682600 1.49225100

H -4.58362400 6.09117500 0.45119700

H -3.36515700 4.00262300 -0.12389300

H 1.92248500 -0.46979900 0.18591700

H 3.72351700 -2.15558300 0.27534000

H 6.71443700 0.90343500 0.21144200

H 6.78181800 3.54727300 0.03347400

H 6.28570900 5.97572700 -0.07532500

H 3.94954800 6.75984100 -0.14415600

H 2.06500200 5.16411400 -0.07552200

H -2.55789200 -3.80269300 -2.25415300

H -0.29357800 -4.25663500 -3.16073700

H 1.48346900 -2.55709300 -2.96075400

H 1.07111400 -0.40864700 -1.83304300

H -3.55640800 1.74465900 0.56954300

H -5.83332600 0.81608000 0.88567800

H -4.74454200 -2.77748800 -1.16208800

H 4.70408100 -2.34917100 2.54002900

H 5.24986500 -4.27650800 3.98993100

H 7.21414700 -5.69270200 3.53357200

H 8.69993000 -5.20067300 1.61645100

H 9.91062800 -4.12286400 -0.62470700

H 10.51514100 -2.75682800 -2.59801100

H 9.11393800 -0.84549400 -3.27321200

H 7.08913700 -0.23420000 -1.99126700

H -10.13087400 -4.11173000 1.15548100

H -8.67769500 -5.88436000 2.08867800

H -6.22813500 -5.61639400 2.10142900

H -5.16329600 -3.58831300 1.16865000

H -7.18289900 0.97957700 -1.22350300

H -9.48173700 1.75787500 -1.70480100

H -11.43830100 0.38905100 -1.09688100

H -11.14196200 -1.81226900 -0.00559600

DMOC-DPS_S0_PBE0

C 6.92055000 5.81702100 0.00439900

O 5.69443100 5.30859600 0.46601600

C 5.48877400 3.97037400 0.36530100

C 4.24321400 3.52189600 0.83806000

C 3.89476000 2.18580400 0.80650600

C 4.82010600 1.27611000 0.29687000

C 6.05874700 1.71468400 -0.20035200

C 6.40104700 3.06918200 -0.16449100

C 6.74561400 0.55016900 -0.70167700

C 7.99678800 0.36279700 -1.27513600

C 8.39601500 -0.92100600 -1.62980600

O 9.62941500 -1.02254100 -2.19159900

C 10.09077400 -2.30083500 -2.54955100

C 7.54880600 -2.01315700 -1.39410600

C 6.29471600 -1.83780000 -0.82017400

C 5.88976500 -0.55246400 -0.49042500

N 4.71598100 -0.10472600 0.11885400

C 3.62552500 -0.90162900 0.49032100

C 3.08889500 -1.82434900 -0.41233200

C 2.01574900 -2.61855800 -0.04301300

C 1.46743300 -2.47935400 1.22655200

S 0.08225400 -3.49567100 1.70000600

O 0.08488000 -3.58172200 3.15428000

O 0.10607700 -4.68069300 0.85271000

C -1.34481000 -2.53733100 1.22863200

C -1.88746600 -2.69752800 -0.04081400

C -2.99255600 -1.94747600 -0.40865700

C -3.56604200 -1.04950600 0.49590400

N -4.68881500 -0.29661300 0.12602200

C -4.84496200 1.07871600 0.29758600

C -3.95538500 2.02599500 0.80281200

C -4.35518300 3.34726500 0.82871800

C -5.61793800 3.74422500 0.35404000

O -5.87477700 5.07431400 0.44928400

C -7.11858000 5.53405100 -0.01452900

C -6.49477900 2.80677200 -0.17141900

C -6.10092100 1.46505300 -0.20241100

C -6.73912200 0.27257600 -0.69723600

C -7.99147700 0.03897300 -1.27470200

C -8.33130900 -1.26074700 -1.61949500

O -9.50799200 -1.62602000 -2.19039700

C -10.44823800 -0.61566200 -2.45214700

C -7.43618800 -2.31735900 -1.37451500

C -6.19780000 -2.09906100 -0.80464100

C -5.84401500 -0.78973000 -0.48096700

C -3.02023400 -0.91360400 1.77568300

C -1.90620300 -1.65128400 2.14086700

C 1.99219100 -1.56923600 2.13699900

C 3.07439100 -0.78652800 1.77014500

H 7.76761000 5.39049800 0.55505000

H 7.05754600 5.63057000 -1.06758800

H 6.88635200 6.89151200 0.17801400

H 3.55468300 4.26382800 1.22781200

H 2.91961400 1.86944000 1.15940800

H 7.35962500 3.39247600 -0.55306100

H 8.67908300 1.18839500 -1.44870700

H 11.08367700 -2.15412300 -2.97225500

H 9.44674900 -2.76863500 -3.30384700

H 10.16697200 -2.96363200 -1.67929600

H 7.86176100 -3.01776400 -1.65172300

H 5.66645300 -2.70009500 -0.62698600

H 3.50764000 -1.90103700 -1.40954200

H 1.60683700 -3.35095200 -0.73027200

H -1.44934700 -3.41178500 -0.72914200

H -3.40849300 -2.04011500 -1.40572200

H -2.96880300 1.74871700 1.15691000

H -3.69637600 4.11746800 1.21487100

H -7.12518600 6.60984200 0.15435900

H -7.94935800 5.07841900 0.53785800

H -7.24818600 5.33809700 -1.08586800

H -7.46487000 3.09339100 -0.56047800

H -8.67416200 0.86523300 -1.43541400

H -11.30515000 -1.11073200 -2.90655700

H -10.77003900 -0.11439300 -1.53120400

H -10.05356800 0.13258600 -3.15024800

H -7.75239000 -3.31938600 -1.64333400

H -5.53543100 -2.93425100 -0.60643800

H -3.49591900 -0.25141800 2.49059000

H -1.49726300 -1.57731600 3.14251700

H 1.58120700 -1.51068500 3.13884800

H 3.52232000 -0.10425900 2.48407400

DMOC-DPS_S0_LC-PBE

C 6.78447000 5.76837600 0.01825200

O 5.57390900 5.26322500 0.48060600

C 5.37516400 3.93612500 0.37741100

C 4.14405700 3.48045300 0.84873100

C 3.80767900 2.15809200 0.81259200

C 4.73219000 1.26463100 0.29749600

C 5.94771300 1.70997900 -0.19340000

C 6.28152100 3.05564400 -0.15334800

C 6.64058900 0.55571400 -0.69778500

C 7.87906500 0.38360000 -1.27272100

C 8.28110700 -0.88227800 -1.62993900

O 9.50139000 -0.98089500 -2.19312300

C 9.95597500 -2.24571700 -2.55122800

C 7.45117400 -1.97142200 -1.39713500

C 6.21139500 -1.80910100 -0.82253300

C 5.80357000 -0.53829800 -0.48831200

N 4.63787100 -0.10459900 0.11916700

C 3.56096800 -0.90542000 0.48923400

C 3.03581600 -1.81582900 -0.40935200

C 1.98025800 -2.61275400 -0.04231000

C 1.44441700 -2.48131500 1.21774500

S 0.08396700 -3.48619400 1.68141700

O 0.08696600 -3.57365900 3.11873600

O 0.10835500 -4.65313200 0.83783500

C -1.31977100 -2.54081100 1.22041900

C -1.84960900 -2.69249300 -0.03985300

C -2.93842000 -1.94056700 -0.40505300

C -3.50171900 -1.05629000 0.49612600

N -4.61208800 -0.29995100 0.12793400

C -4.75920000 1.06359600 0.29985400

C -3.87112100 1.99580000 0.81206100

C -4.25964800 3.30302100 0.84224700

C -5.50854700 3.70638500 0.36720200

O -5.75930100 5.02531400 0.46511200

C -6.98754700 5.48138100 -0.00018900

C -6.37850400 2.78932500 -0.16050600

C -5.99208600 1.45599000 -0.19555000

C -6.63407500 0.27307200 -0.69405600

C -7.87583100 0.05411600 -1.27600800

C -8.21590700 -1.22651100 -1.62305400

O -9.38244800 -1.57554000 -2.19680400

C -10.30006100 -0.56294700 -2.45288700

C -7.33543900 -2.28185700 -1.37912300

C -6.11392200 -2.07693700 -0.80792200

C -5.75839300 -0.77857300 -0.47873000

C -2.97124300 -0.92964300 1.76682600

C -1.87282700 -1.66852400 2.12927000

C 1.95941900 -1.58342500 2.12410400

C 3.02472300 -0.79862500 1.75965500

H 7.63256000 5.34011800 0.56401500

H 6.91479600 5.58063200 -1.05332800

H 6.75784000 6.84262400 0.18955900

H 3.45605900 4.22068000 1.24267500

H 2.83629500 1.83013800 1.16637100

H 7.23778800 3.38538400 -0.54230900

H 8.55563800 1.21321100 -1.44888700

H 10.94669800 -2.10694800 -2.97957400

H 9.30697500 -2.71097000 -3.30132900

H 10.03453100 -2.90796100 -1.68204500

H 7.77186800 -2.97214400 -1.66047300

H 5.58786400 -2.67507600 -0.62835300

H 3.45540600 -1.88010900 -1.40752800

H 1.57106200 -3.34953900 -0.72584200

H -1.40953400 -3.40963200 -0.72507300

H -3.35518000 -2.02060300 -1.40332500

H -2.88805000 1.70770500 1.16839400

H -3.60278500 4.07232100 1.23349700

H -7.00287800 6.55652600 0.16696300

H -7.81914000 5.02274700 0.54647600

H -7.10927200 5.28478600 -1.07129400

H -7.34624800 3.08188000 -0.55096300

H -8.55073700 0.88633100 -1.43875500

H -11.16425200 -1.03673500 -2.91419800

H -10.61641300 -0.06488300 -1.52952700

H -9.89128600 0.18402300 -3.14261200

H -7.66233000 -3.27837900 -1.65558800

H -5.45549900 -2.91521700 -0.60738300

H -3.45152200 -0.26742700 2.47926300

H -1.46348500 -1.60801700 3.13246500

H 1.54819500 -1.53807100 3.12734300

H 3.47573000 -0.11471000 2.47070800

DMOC-DPS_S0_LC-PBE0*

C 6.88724700 5.75472200 -0.01888000

O 5.67487400 5.27701700 0.48465900

C 5.45515500 3.94874700 0.38216300

C 4.22767900 3.50551800 0.89064000

C 3.87141900 2.17645200 0.86079900

C 4.77378100 1.27092100 0.31447400

C 5.99109900 1.70576800 -0.21924600

C 6.34259800 3.05287700 -0.18465400

C 6.65140100 0.54410100 -0.75337500

C 7.87611900 0.35620600 -1.36949500

C 8.25103000 -0.92348400 -1.74511700

O 9.45551200 -1.03435300 -2.34839800

C 9.86999900 -2.31593800 -2.71931400

C 7.41036200 -2.00937800 -1.48784500

C 6.18191300 -1.83239600 -0.87142200

C 5.79974500 -0.54996700 -0.52175000

N 4.65673600 -0.10132100 0.12902500

C 3.58097800 -0.88988800 0.52871400

C 3.05282100 -1.84081200 -0.34138100

C 1.99129700 -2.62859900 0.05744900

C 1.45310500 -2.44987800 1.32038400

S 0.08459800 -3.44446600 1.82378200

O 0.08646600 -3.47496500 3.27452100

O 0.10976200 -4.64848100 1.01403500

C -1.32691400 -2.50968200 1.32335300

C -1.85636100 -2.70738500 0.05959200

C -2.95085600 -1.96475500 -0.33707200

C -3.51996200 -1.04152000 0.53663000

N -4.62888200 -0.29764400 0.13914900

C -4.80022300 1.06785600 0.32133300

C -3.93622800 2.01047300 0.86757600

C -4.34603400 3.32351700 0.89433600

C -5.59021300 3.71480900 0.38228700

O -5.86339500 5.03363000 0.48234500

C -7.09227400 5.46195400 -0.02425200

C -6.43953600 2.78343200 -0.18446800

C -6.03419900 1.45026600 -0.21688200

C -6.64275400 0.26191000 -0.74860700

C -7.86718200 0.02826900 -1.37230600

C -8.17983500 -1.26632600 -1.74130200

O -9.32667000 -1.62729100 -2.35626300

C -10.24217700 -0.60865300 -2.62893000

C -7.29048800 -2.31594800 -1.47509300

C -6.07961500 -2.09656800 -0.86014200

C -5.75117100 -0.79070000 -0.51283900

C -2.98777700 -0.86878600 1.81226800

C -1.88371200 -1.59889200 2.20517900

C 1.96926400 -1.51213900 2.19879700

C 3.04028600 -0.73575800 1.80346900

H 7.74337700 5.30436800 0.49972900

H 6.97944000 5.56269800 -1.09562000

H 6.88863000 6.83006700 0.15423000

H 3.55885900 4.25173600 1.30777100

H 2.90511500 1.85938600 1.24005700

H 7.29007200 3.37559200 -0.60356400

H 8.55895200 1.17849100 -1.56161100

H 10.84955200 -2.19745100 -3.18083600

H 9.18356900 -2.76874500 -3.44616400

H 9.96099900 -2.97943000 -1.84988400

H 7.71170700 -3.01398400 -1.76353100

H 5.55472600 -2.69193500 -0.65694100

H 3.46946700 -1.93900600 -1.33886600

H 1.57994800 -3.39054500 -0.59789100

H -1.41233500 -3.44852900 -0.59824700

H -3.36256100 -2.07738700 -1.33511800

H -2.95880600 1.73304100 1.24952300

H -3.70943600 4.09747900 1.31122400

H -7.13696700 6.53667300 0.14711300

H -7.93109300 4.97873700 0.49331500

H -7.17448600 5.26495400 -1.10102300

H -7.39818500 3.06912800 -0.60507700

H -8.55152500 0.85160200 -1.54932500

H -11.08972600 -1.08202600 -3.12284700

H -10.58789300 -0.12108500 -1.70818900

H -9.81295000 0.14982900 -3.29639900

H -7.59359400 -3.31655800 -1.76653600

H -5.41769900 -2.92849200 -0.64119100

H -3.47217900 -0.18462000 2.50198200

H -1.47545300 -1.50517000 3.20710600

H 1.55716100 -1.43253300 3.20038500

H 3.49366600 -0.02860200 2.49107000

PIC-TRZ_S0_PBE0

C -12.10307800 -0.00003000 0.00006900

C -11.40339900 -1.05123200 -0.58101600

C -10.01543800 -1.05197200 -0.57937800

C -9.29877100 -0.00001000 0.00002700

C -7.82220500 0.00000100 0.00000600

C -7.10241700 -1.17976100 0.22165200

C -5.71820800 -1.18296100 0.21815600

C -5.00829200 0.00002200 -0.00003300

C -3.53486300 0.00003100 -0.00005300

N -2.91984200 -1.18293400 -0.02651200

C -1.58967100 -1.12189300 -0.01473500

N -0.89176000 -2.31207400 -0.01252400

C -1.38644500 -3.56394600 -0.44459600

C -2.62605200 -3.91430500 -0.96895600

C -2.81726300 -5.23502000 -1.35851700

C -1.80654600 -6.18834200 -1.23368100

C -0.56908500 -5.83041700 -0.72112100

C -0.35437400 -4.51262200 -0.32886200

C 0.80413400 -3.83838300 0.20728600

C 2.07077000 -4.29657800 0.53958900

C 2.97585200 -3.39330200 1.08342300

C 4.34867700 -3.49403000 1.51344300

C 5.27288800 -4.53702600 1.54965400

C 6.54207900 -4.29811600 2.05405500

C 6.89069300 -3.02970300 2.53115400

C 5.99036000 -1.97409500 2.50699600

C 4.72307400 -2.21615300 1.98478300

N 3.64393200 -1.35133200 1.85411400

C 3.61123800 0.00429300 2.26012400

C 4.53608600 0.91372900 1.75201900

C 4.48782100 2.24487800 2.14434000

C 3.51178300 2.67702200 3.03412700

C 2.58990000 1.76885600 3.54150900

C 2.64214900 0.43361400 3.16504100

C 2.57971300 -2.05055800 1.30200900

C 1.32494700 -1.56084300 0.95671700

C 0.45072400 -2.47997200 0.39474900

N -0.87506000 0.00004300 -0.00001500

C -1.58968100 1.12197100 0.01468300

N -0.89176400 2.31215000 0.01248300

C -1.38641600 3.56402800 0.44458200

C -2.62601400 3.91442200 0.96894000

C -2.81718500 5.23513600 1.35852400

C -1.80643500 6.18842700 1.23371300

C -0.56898200 5.83046900 0.72115900

C -0.35431200 4.51267400 0.32887700

C 0.80418200 3.83840900 -0.20726900

C 2.07083700 4.29656600 -0.53954900

C 2.97589400 3.39327100 -1.08339300

C 4.34872800 3.49396000 -1.51339500

C 5.27297600 4.53692400 -1.54957400

C 6.54216600 4.29797800 -2.05396300

C 6.89074100 3.02956000 -2.53108000

C 5.99037000 1.97398500 -2.50695500

C 4.72308500 2.21607900 -1.98475700

N 3.64391400 1.35129200 -1.85411100

C 3.61117300 -0.00432000 -2.26016200

C 2.64208900 -0.43357500 -3.16511600

C 2.58979400 -1.76880400 -3.54162200

C 3.51162800 -2.67702200 -3.03424200

C 4.48766300 -2.24494300 -2.14442100

C 4.53597200 -0.91380800 -1.75206000

C 2.57971500 2.05054200 -1.30200100

C 1.32492800 1.56086300 -0.95673200

C 0.45073000 2.48001300 -0.39476200

N -2.91985300 1.18300100 0.02642800

C -5.71823200 1.18299400 -0.21820300

C -7.10244000 1.17977400 -0.22166100

C -10.01543700 1.05194200 0.57945400

C -11.40339800 1.05118100 0.58113300

H -13.18865600 -0.00003900 0.00008500

H -11.94116100 -1.87227000 -1.04537400

H -9.47768900 -1.86421900 -1.05927800

H -7.63796900 -2.10090500 0.42961100

H -5.16920500 -2.09843000 0.40804100

H -3.41622700 -3.18479700 -1.06387700

H -3.78055000 -5.52515800 -1.76659600

H -1.98972400 -7.21284400 -1.54199100

H 0.22455200 -6.56605100 -0.63008100

H 2.34295000 -5.33619400 0.38308900

H 5.00055800 -5.52642000 1.19330100

H 7.27064000 -5.10187900 2.08727800

H 7.88569400 -2.86553000 2.93338100

H 6.26359300 -0.99711500 2.89053600

H 5.28082600 0.58115700 1.03739100

H 5.20266200 2.94860900 1.73029100

H 3.46548800 3.72078800 3.32745800

H 1.82737700 2.09914700 4.23986700

H 1.93641900 -0.28416700 3.57015900

H 1.05985600 -0.52491900 1.09850000

H -3.41621400 3.18494000 1.06384400

H -3.78046400 5.52529900 1.76660200

H -1.98958000 7.21292900 1.54204200

H 0.22468100 6.56607800 0.63014200

H 2.34305000 5.33617100 -0.38302800

H 5.00067700 5.52632100 -1.19320600

H 7.27075600 5.10171400 -2.08716100

H 7.88574200 2.86536000 -2.93329600

H 6.26357200 0.99700000 -2.89050700

H 1.93639800 0.28424700 -3.57022900

H 1.82727500 -2.09904500 -4.24000800

H 3.46529800 -3.72077800 -3.32760300

H 5.20246600 -2.94871400 -1.73037500

H 5.28071000 -0.58128300 -1.03740800

H 1.05980300 0.52495100 -1.09853800

H -5.16924800 2.09847000 -0.40810900

H -7.63801200 2.10091000 -0.42960700

H -9.47768600 1.86419800 1.05933800

H -11.94115800 1.87221100 1.04550700

PIC-TRZ_S0_LC-PBE

C -12.01508100 -0.00002400 -0.00009200

C -11.32290100 -1.03051800 -0.59592600

C -9.94729600 -1.03083500 -0.59391900

C -9.24302000 -0.00001600 -0.00002600

C -7.77216100 -0.00001200 0.00001000

C -7.06651900 -1.16377900 0.24913700

C -5.69378800 -1.16673800 0.24556800

C -4.99782400 -0.00000300 0.00008500

C -3.52986300 0.00000200 0.00013400

N -2.92065700 -1.16896300 -0.02020500

C -1.60295900 -1.11471200 -0.00819100

N -0.90724100 -2.28915700 0.00063300

C -1.40431800 -3.54915900 -0.36790000

C -2.64833800 -3.92061900 -0.83231900

C -2.84257800 -5.24268400 -1.16053100

C -1.83219300 -6.17811900 -1.03414100

C -0.59296000 -5.79809800 -0.58134800

C -0.37845800 -4.47751100 -0.25200400

C 0.79099700 -3.78143500 0.22122800

C 2.05449700 -4.21945800 0.53167700

C 2.96169700 -3.29912800 1.00646500

C 4.34179000 -3.37685600 1.40144500

C 5.25976400 -4.40778300 1.44925700

C 6.53143100 -4.14904300 1.89481500

C 6.88558800 -2.87142400 2.30551000

C 5.99046800 -1.83153100 2.26876600

C 4.71779300 -2.09551200 1.80011300

N 3.64197200 -1.24575600 1.66578100

C 3.61261900 0.12097000 1.99172900

C 4.57847400 0.97890100 1.50854600

C 4.52371300 2.31898700 1.81382900

C 3.49698300 2.81248900 2.58664700

C 2.53712000 1.95464100 3.07430600

C 2.60011500 0.61024500 2.79096900

C 2.57542800 -1.96639100 1.17687000

C 1.31797300 -1.50088900 0.85746900

C 0.43961100 -2.43518300 0.36689500

N -0.90350100 0.00001200 -0.00004900

C -1.60296100 1.11473200 0.00818800

N -0.90723800 2.28917000 -0.00063500

C -1.40431200 3.54918900 0.36786600

C -2.64832900 3.92069000 0.83226900

C -2.84255900 5.24277200 1.16041200

C -1.83217100 6.17819800 1.03397700

C -0.59294000 5.79814000 0.58121000

C -0.37845100 4.47753400 0.25193700

C 0.79100500 3.78144000 -0.22126300

C 2.05450300 4.21945700 -0.53171600

C 2.96170800 3.29912200 -1.00648900

C 4.34179400 3.37685800 -1.40149200

C 5.25973300 4.40781100 -1.44937100

C 6.53137800 4.14910500 -1.89501200

C 6.88553700 2.87149600 -2.30572800

C 5.99044700 1.83157800 -2.26891700

C 4.71779800 2.09551800 -1.80017200

N 3.64199300 1.24574400 -1.66578500

C 3.61268600 -0.12100400 -1.99163500

C 2.60006500 -0.61042900 -2.79063000

C 2.53711900 -1.95485800 -3.07383600

C 3.49714900 -2.81258800 -2.58631000

C 4.52400900 -2.31893400 -1.81375700

C 4.57871700 -0.97882400 -1.50858900

C 2.57544700 1.96637900 -1.17686200

C 1.31799900 1.50088000 -0.85742400

C 0.43962400 2.43518400 -0.36688500

N -2.92066300 1.16896800 0.02036600

C -5.69378300 1.16672500 -0.24544200

C -7.06651300 1.16375900 -0.24908200

C -9.94733000 1.03079800 0.59383200

C -11.32293500 1.03047400 0.59577400

H -13.10047500 -0.00002700 -0.00011800

H -11.86131900 -1.84246500 -1.07469200

H -9.40530100 -1.83439000 -1.08407700

H -7.60732500 -2.07783100 0.47485100

H -5.13878200 -2.07509900 0.45476700

H -3.44295300 -3.19562800 -0.93012300

H -3.81624000 -5.55344500 -1.52605700

H -2.02001000 -7.21400400 -1.29729600

H 0.20858400 -6.52442800 -0.48658400

H 2.32610600 -5.26408700 0.41070300

H 4.97699400 -5.41066900 1.14252900

H 7.26408300 -4.94821100 1.93784000

H 7.89180500 -2.68926900 2.66995800

H 6.26982400 -0.84053100 2.60957200

H 5.37300500 0.60007400 0.87585800

H 5.28185100 2.98626000 1.41651400

H 3.44306400 3.87302700 2.80931400

H 1.72972100 2.33510900 3.69173700

H 1.86169900 -0.07557300 3.19339400

H 1.04813900 -0.46369800 0.97862100

H -3.44294400 3.19571100 0.93012800

H -3.81622000 5.55355600 1.52592300

H -2.01998300 7.21409600 1.29708000

H 0.20861600 6.52445300 0.48641200

H 2.32610500 5.26409200 -0.41077500

H 4.97694700 5.41069100 -1.14264100

H 7.26400600 4.94829300 -1.93809400

H 7.89173100 2.68936300 -2.67024900

H 6.26981300 0.84059300 -2.60975900

H 1.86151800 0.07528600 -3.19298700

H 1.72961900 -2.33543900 -3.69106500

H 3.44326100 -3.87315100 -2.80886400

H 5.28228700 -2.98611200 -1.41654900

H 5.37335700 -0.59988200 -0.87610600

H 1.04815600 0.46369100 -0.97854900

H -5.13877400 2.07509000 -0.45461400

H -7.60731300 2.07780700 -0.47482400

H -9.40536300 1.83435600 1.08401600

H -11.86138000 1.84241700 1.07451400

PIC-TRZ_S0_LC-PBE0*

C -12.06458800 -0.00001200 0.00022400

C -11.36847300 -1.03272800 -0.60601600

C -9.98520200 -1.03301900 -0.60475900

C -9.27550600 0.00000200 0.00018900

C -7.80473500 0.00000800 0.00017400

C -7.09341300 -1.18495800 0.17473500

C -5.71349100 -1.18804500 0.17104000

C -5.01289700 0.00002000 0.00014400

C -3.54565900 0.00001300 0.00009300

N -2.93643700 -1.17850000 -0.05168900

C -1.61143200 -1.11777600 -0.03358300

N -0.91312200 -2.29605600 -0.04378600

C -1.41998300 -3.55666400 -0.40676100

C -2.67357200 -3.92102100 -0.87187300

C -2.87748300 -5.25208200 -1.19592100

C -1.86850500 -6.19900800 -1.06336900

C -0.61824200 -5.82576600 -0.60825600

C -0.39133000 -4.49835000 -0.28268500

C 0.78233600 -3.80571800 0.18941200

C 2.04863000 -4.24991600 0.51962600

C 2.96122000 -3.32386900 0.99600500

C 4.34043700 -3.40454300 1.39632500

C 5.25726100 -4.44493500 1.45461400

C 6.53987800 -4.18517700 1.89695800

C 6.90310300 -2.89961100 2.29659600

C 6.00825600 -1.84700800 2.24955100

C 4.72839300 -2.10909100 1.78060200

N 3.65363500 -1.25205900 1.63303100

C 3.64763600 0.13241400 1.88052900

C 4.68817700 0.93581200 1.43155900

C 4.65103500 2.30208300 1.64340500

C 3.56695400 2.87977900 2.28338500

C 2.53553100 2.07840300 2.74171500

C 2.58138100 0.70737400 2.55940800

C 2.58004800 -1.97547700 1.14657000

C 1.32127700 -1.50491900 0.81054500

C 0.43654600 -2.44687900 0.32328500

N -0.90148600 -0.00000500 -0.00041100

C -1.61139300 1.11778700 0.03329400

N -0.91305400 2.29603200 0.04353800

C -1.41997300 3.55670600 0.40626700

C -2.67358500 3.92116600 0.87124500

C -2.87752600 5.25229700 1.19499600

C -1.86856000 6.19921000 1.06231000

C -0.61826200 5.82587100 0.60737000

C -0.39132900 4.49838700 0.28210900

C 0.78239200 3.80570700 -0.18977200

C 2.04872500 4.24990300 -0.51979700

C 2.96143500 3.32383600 -0.99592400

C 4.34071200 3.40449200 -1.39603200

C 5.25751800 4.44490000 -1.45429000

C 6.54019600 4.18513900 -1.89645300

C 6.90349100 2.89955400 -2.29595700

C 6.00866200 1.84693300 -2.24894000

C 4.72873300 2.10901300 -1.78016300

N 3.65397000 1.25197700 -1.63267000

C 3.64793500 -0.13247300 -1.88031800

C 2.58176300 -0.70730800 -2.55943600

C 2.53588400 -2.07831800 -2.74189900

C 3.56719000 -2.87979200 -2.28348300

C 4.65119000 -2.30222200 -1.64325200

C 4.68836100 -0.93597600 -1.43125800

C 2.58030200 1.97543300 -1.14641400

C 1.32150300 1.50486100 -0.81049200

C 0.43665200 2.44683800 -0.32348000

N -2.93640300 1.17850000 0.05187200

C -5.71350400 1.18807900 -0.17073900

C -7.09342600 1.18497900 -0.17440300

C -9.98519800 1.03301500 0.60515600

C -11.36846800 1.03271100 0.60644600

H -13.15076100 -0.00001800 0.00023700

H -11.90738600 -1.84158400 -1.09121600

H -9.44245800 -1.83126500 -1.10499300

H -7.63535200 -2.11144800 0.34535800

H -5.15586000 -2.10733500 0.32458400

H -3.46172000 -3.18714700 -0.97161800

H -3.85392100 -5.55790000 -1.56052700

H -2.06430700 -7.23543800 -1.32185700

H 0.17785100 -6.55900900 -0.50916900

H 2.31624500 -5.29834100 0.41276900

H 4.96776500 -5.45058200 1.16005500

H 7.26906500 -4.98823900 1.94676100

H 7.91091000 -2.71885600 2.65958900

H 6.29393500 -0.85491500 2.58467500

H 5.52314700 0.49337100 0.89863600

H 5.46311500 2.92046900 1.27123900

H 3.52474700 3.95575400 2.42278600

H 1.68743800 2.52202900 3.25517200

H 1.79144000 0.07232300 2.94888100

H 1.04950100 -0.46588900 0.91769400

H -3.46172300 3.18730900 0.97115600

H -3.85399100 5.55816500 1.55948900

H -2.06439100 7.23569200 1.32056700

H 0.17784500 6.55908400 0.50816900

H 2.31625300 5.29836400 -0.41306400

H 4.96795700 5.45056300 -1.15985100

H 7.26936900 4.98821500 -1.94623800

H 7.91134400 2.71879200 -2.65881900

H 6.29440300 0.85483600 -2.58399200

H 1.79192400 -0.07219200 -2.94900200

H 1.68785400 -2.52185000 -3.25554000

H 3.52492600 -3.95575500 -2.42297500

H 5.46317600 -2.92068900 -1.27101300

H 5.52324700 -0.49362400 -0.89813100

H 1.04974500 0.46583600 -0.91767200

H -5.15589100 2.10737600 -0.32430000

H -7.63537700 2.11146400 -0.34501600

H -9.44245000 1.83126600 1.10537700

H -11.90737800 1.84156000 1.09165900

PPZ-3TPT_S0_PBE0

C -9.56120700 -0.01288000 0.00996900

C -8.62354100 1.01201600 0.01291900

C -7.27835700 0.73510500 -0.19380800

C -6.85430600 -0.58059600 -0.40177200

C -5.46257200 -0.95192200 -0.66479700

N -5.11756700 -1.98733900 -1.39309000

N -3.76974200 -2.04902200 -1.43567800

C -3.28698000 -1.05297900 -0.73219300

C -1.85292000 -0.81462000 -0.56001100

C -0.99896300 -1.27375100 -1.57027600

C 0.37242700 -1.11634100 -1.45937300

C 0.91851300 -0.49849100 -0.33644600

N 2.32583700 -0.32840500 -0.22048700

C 2.92185900 0.82876300 -0.74104100

C 2.17188400 1.81271800 -1.37461600

C 2.77169600 2.96694100 -1.87784700

C 4.13518600 3.13982900 -1.75033300

C 4.90040700 2.15831500 -1.12092400

C 4.31536300 1.00418500 -0.61287000

N 5.07469200 0.01691000 0.02981900

C 6.48256500 0.19037400 0.15137200

C 7.01105800 0.79369600 1.28850700

C 8.38521500 0.96356400 1.40870500

C 9.23191300 0.53143800 0.39413100

C 8.70352100 -0.07150400 -0.74219200

C 7.33012800 -0.24260700 -0.86479400

C 4.49072400 -1.17968900 0.46998100

C 5.25084000 -2.19558800 1.03766300

C 4.65931800 -3.37722000 1.48339800

C 3.29533700 -3.54900300 1.35878000

C 2.52113800 -2.53898000 0.78843700

C 3.09759800 -1.35536800 0.34303400

C 0.07827100 -0.05194200 0.67723800

C -1.29673200 -0.20950300 0.57087000

N -4.32777200 -0.31472700 -0.21369100

C -4.24756400 0.84723900 0.60494600

C -4.67266300 0.78755700 1.92777000

C -4.59316400 1.92385300 2.72228300

C -4.08448300 3.10816300 2.20037600

C -3.65858100 3.15850400 0.87758600

C -3.74295400 2.02931400 0.07351600

C -7.80682200 -1.60612100 -0.41614900

C -9.14774800 -1.32263000 -0.21112500

H -10.61152900 0.20836200 0.17291200

H -8.93930500 2.03890200 0.16914500

H -6.56605100 1.55175000 -0.20955300

H -1.43305100 -1.76155600 -2.43555500

H 1.03393800 -1.46857400 -2.24427200

H 1.10142600 1.67607400 -1.47476200

H 2.15864900 3.71736400 -2.36632400

H 4.62199900 4.03024500 -2.13456200

H 5.97083800 2.29424300 -1.02056000

H 6.33553800 1.12476500 2.07076600

H 8.79528100 1.43428900 2.29686900

H 10.30520000 0.66442000 0.48894500

H 9.36260300 -0.41001900 -1.53566500

H 6.90173600 -0.71152700 -1.74493600

H 6.32149700 -2.06027300 1.13658800

H 5.27982400 -4.15062600 1.92412300

H 2.81525300 -4.46088600 1.69829400

H 1.45028800 -2.67431300 0.69101300

H 0.51262100 0.41415000 1.55560100

H -1.92915600 0.12723200 1.38366300

H -5.06468800 -0.14415900 2.32162100

H -4.92738300 1.88147600 3.75381800

H -4.01960000 3.99302300 2.82535000

H -3.26049300 4.08089300 0.46738700

H -3.41817100 2.05410500 -0.96135400

H -7.47337300 -2.62128200 -0.59945900

H -9.87444500 -2.12903400 -0.22268600

PPZ-3TPT_S0_LC-PBE

C -9.45095300 0.02973500 0.03151100

C -8.51568500 1.03734300 -0.03176200

C -7.18844400 0.74037500 -0.24305600

C -6.78832400 -0.57427400 -0.38846300

C -5.40568500 -0.96365800 -0.64953600

N -5.06985600 -1.99913700 -1.34681000

N -3.73167400 -2.06581600 -1.38327300

C -3.25760400 -1.07350400 -0.70468200

C -1.82990000 -0.82588000 -0.52630500

C -0.98076600 -1.25738800 -1.53039700

C 0.37568000 -1.08704800 -1.41160400

C 0.90038800 -0.48610200 -0.28606000

N 2.29436500 -0.30473800 -0.15840800

C 2.88455900 0.82062700 -0.72690900

C 2.14354500 1.76514100 -1.39418400

C 2.74031800 2.89178300 -1.93160400

C 4.08890200 3.06933800 -1.80390400

C 4.84577300 2.12062300 -1.13966700

C 4.26275000 1.00094700 -0.59811400

N 5.00778400 0.04941800 0.09234900

C 6.40493700 0.22448700 0.20683700

C 6.92919500 0.81609400 1.33380500

C 8.29084700 0.98803000 1.44879800

C 9.12587800 0.56858100 0.43751700

C 8.60002500 -0.02373700 -0.69049900

C 7.24004300 -0.19613000 -0.80646500

C 4.44804500 -1.17151200 0.46078000

C 5.21474800 -2.19445700 0.96324000

C 4.64116400 -3.39338900 1.34701100

C 3.29221600 -3.57037900 1.22080500

C 2.51150000 -2.54857100 0.71082100

C 3.07039000 -1.35231000 0.33281900

C 0.06191800 -0.06853600 0.72123500

C -1.29888200 -0.23846900 0.60576900

N -4.28431300 -0.32978000 -0.20998000

C -4.20339400 0.83803800 0.58278900

C -4.68447100 0.81711900 1.87175100

C -4.60459900 1.95602700 2.63936600

C -4.03864300 3.10047600 2.12186000

C -3.55645300 3.10999500 0.83139000

C -3.64250600 1.97804200 0.05428500

C -7.73356300 -1.58232400 -0.33875200

C -9.05723500 -1.28109500 -0.12813200

H -10.49667900 0.26798400 0.19898300

H -8.82223500 2.07274100 0.07741000

H -6.46605200 1.54677600 -0.30936000

H -1.41126000 -1.74096700 -2.40069900

H 1.04974000 -1.41988500 -2.19478800

H 1.07308900 1.62325400 -1.49164900

H 2.13060000 3.62412400 -2.45019400

H 4.57526400 3.94649400 -2.21740500

H 5.91554100 2.26177100 -1.03422200

H 6.25162800 1.13837100 2.11803500

H 8.70369700 1.45428500 2.33773900

H 10.19900900 0.70412200 0.52858700

H 9.25721400 -0.35511800 -1.48823900

H 6.80541400 -0.66072500 -1.68636500

H 6.28440800 -2.05089100 1.06658600

H 5.26968000 -4.18260100 1.74579200

H 2.82402400 -4.50330900 1.51615300

H 1.44050700 -2.68684600 0.61346200

H 0.49305300 0.38790000 1.60622200

H -1.94368700 0.07929200 1.41771800

H -5.12483200 -0.09469100 2.26220100

H -4.98625900 1.94753100 3.65499000

H -3.97234600 3.99574100 2.73155700

H -3.10949500 4.01028400 0.42282500

H -3.27141800 1.96654100 -0.96554100

H -7.40575700 -2.60649600 -0.48165500

H -9.79130000 -2.07954600 -0.08902800

PPZ-3TPT_S0_LC-PBE0*

C -9.51751000 -0.01650100 -0.03548700

C -8.57758200 0.99799700 0.05132400

C -7.23323700 0.72744000 -0.14717800

C -6.81740200 -0.57234600 -0.43055700

C -5.42892100 -0.93797800 -0.68546600

N -5.07121000 -1.95541000 -1.42689700

N -3.72660300 -2.01012400 -1.45120000

C -3.26131000 -1.02744000 -0.72372900

C -1.83864800 -0.77987100 -0.52197100

C -0.96604900 -1.26218800 -1.49957900

C 0.39778100 -1.09494900 -1.36324500

C 0.91117100 -0.44370400 -0.24749600

N 2.30802600 -0.26035200 -0.10237900

C 2.91630700 0.81544800 -0.75529000

C 2.18296100 1.72918700 -1.49453500

C 2.80060500 2.81713500 -2.10344900

C 4.16240900 2.98668600 -1.98168500

C 4.91083800 2.06830000 -1.25218400

C 4.30517000 0.98685000 -0.63310100

N 5.03441200 0.07470900 0.13441000

C 6.44095900 0.22872300 0.22190500

C 6.99313700 0.86079900 1.32513100

C 8.36857800 1.01379500 1.41592000

C 9.18837500 0.53548700 0.40522800

C 8.63397900 -0.09715400 -0.69866700

C 7.26039600 -0.25133900 -0.79167500

C 4.46743500 -1.14635200 0.51204200

C 5.23477700 -2.17831000 1.02739500

C 4.64723200 -3.37213200 1.43371500

C 3.28500200 -3.54037100 1.31407100

C 2.50580400 -2.51483500 0.78812300

C 3.07891500 -1.31794600 0.39079100

C 0.05258400 0.02502200 0.73313100

C -1.31644500 -0.14291700 0.60217100

N -4.30850100 -0.30832400 -0.20938900

C -4.24567400 0.83178400 0.62871000

C -4.70558300 0.74602200 1.93379600

C -4.64243300 1.86387500 2.74951600

C -4.11506900 3.05184300 2.26435400

C -3.65431700 3.12613600 0.95786600

C -3.72277900 2.01598400 0.13213400

C -7.76965500 -1.58691300 -0.53009800

C -9.10936200 -1.30908800 -0.33321100

H -10.56961400 0.20092700 0.12189100

H -8.89164100 2.01448900 0.26785300

H -6.51371300 1.53812200 -0.09690500

H -1.38637200 -1.77662700 -2.35746900

H 1.08242700 -1.46426200 -2.12108800

H 1.11038300 1.59175400 -1.58536500

H 2.20186700 3.52270200 -2.67039000

H 4.66098100 3.82962800 -2.44915300

H 5.98323600 2.19914800 -1.15099600

H 6.33100000 1.22663300 2.10377200

H 8.80139700 1.50856700 2.27997900

H 10.26507700 0.65562900 0.47729200

H 9.27516900 -0.47231000 -1.49048800

H 6.80614400 -0.74373600 -1.64668300

H 6.30668200 -2.03957300 1.12325600

H 5.27013000 -4.16113300 1.84249200

H 2.80986000 -4.46483500 1.62567500

H 1.43212800 -2.64188800 0.69529800

H 0.47184700 0.51676900 1.60517500

H -1.97134700 0.21170200 1.39099100

H -5.11356700 -0.19313100 2.29420900

H -5.00457200 1.80470000 3.77086100

H -4.06258400 3.92435300 2.90785100

H -3.24047900 4.05438800 0.57727100

H -3.36951300 2.05348700 -0.89367500

H -7.43148600 -2.58860800 -0.77445000

H -9.84093900 -2.10745500 -0.41134900

PPZ-DPO_S0_PBE0

C -9.62123600 -0.00200100 1.80913300

C -8.38016600 -0.00321700 2.43516400

C -7.21695800 -0.00236900 1.67862900

C -7.29353800 -0.00028500 0.28436600

C -6.09577700 0.00063000 -0.53545300

N -6.00611800 0.00218700 -1.82744200

N -4.66987700 0.00239400 -2.13130100

C -4.03115200 0.00115600 -1.00550500

C -2.59639900 0.00087900 -0.78587800

C -2.06018800 0.00001400 0.50344400

C -0.68499800 -0.00019900 0.68236300

C 0.16481800 0.00044800 -0.41983400

N 1.57337000 0.00023700 -0.23159300

C 2.25499000 1.22368300 -0.14940300

C 1.58684400 2.43781800 -0.25336400

C 2.27242900 3.64919800 -0.16646100

C 3.63936200 3.64812900 0.02541300

C 4.32214600 2.43672400 0.13002600

C 3.65142900 1.22206200 0.04573600

N 4.32902500 -0.00017100 0.15239200

C 5.74018200 -0.00038400 0.34243700

C 6.26818600 -0.00124400 1.63017700

C 7.64553000 -0.00146400 1.81485900

C 8.49591200 -0.00081700 0.71476700

C 7.96796100 0.00004900 -0.57167400

C 6.59114200 0.00026200 -0.75917200

C 3.65123300 -1.22219100 0.04454000

C 4.32176100 -2.43704300 0.12757900

C 3.63878400 -3.64823500 0.02176400

C 2.27184700 -3.64889600 -0.17009000

C 1.58645400 -2.43732100 -0.25577600

C 2.25479200 -1.22339600 -0.15059200

C -0.37007300 0.00130900 -1.70732300

C -1.74190400 0.00152400 -1.89280000

O -4.87536700 -0.00000100 0.05388300

C -8.54258000 0.00094100 -0.34399300

C -9.69852200 0.00007600 0.41918500

H -10.52937700 -0.00266900 2.40381800

H -8.31676300 -0.00484200 3.51865600

H -6.24806300 -0.00332900 2.16604700

H -2.72034200 -0.00047500 1.36380800

H -0.25622900 -0.00085700 1.67897000

H 0.51361300 2.43951800 -0.40460900

H 1.72230200 4.58052400 -0.25142500

H 4.19218800 4.57913600 0.09579100

H 5.39530200 2.43632900 0.28064300

H 5.59017500 -0.00173700 2.47767500

H 8.05533600 -0.00213800 2.82013300

H 9.57176000 -0.00098800 0.86012100

H 8.62986900 0.00055400 -1.43213200

H 6.16277200 0.00092900 -1.75641700

H 5.39492300 -2.43697100 0.27815200

H 4.19146600 -4.57939800 0.09118600

H 1.72157400 -4.58005000 -0.25598600

H 0.51322500 -2.43869500 -0.40703800

H 0.30318900 0.00179600 -2.55813900

H -2.17052800 0.00219100 -2.88887800

H -8.58568200 0.00255800 -1.42758600

H -10.66641800 0.00103500 -0.07181800

PPZ-DPO_S0_LC-PBE

C 9.55647100 0.00007400 1.77089500

C 8.33380400 0.00011000 2.40363700

C 7.17446300 0.00007400 1.66318600

C 7.24319900 0.00000200 0.28534200

C 6.03969600 -0.00003600 -0.52211800

N 5.93890700 -0.00004900 -1.79437000

N 4.61107000 -0.00011800 -2.08211600

C 3.99348900 -0.00004100 -0.96598200

C 2.56322000 -0.00003200 -0.73101800

C 2.05342700 0.00005600 0.55035500

C 0.69143200 0.00005900 0.74315200

C -0.15789800 -0.00002200 -0.33968000

N -1.55335200 -0.00001400 -0.13372300

C -2.23856200 -1.21318000 -0.15175700

C -1.58615100 -2.40779500 -0.33452500

C -2.27682100 -3.60641600 -0.32369500

C -3.62991300 -3.60569200 -0.13419500

C -4.29738500 -2.40729400 0.04433100

C -3.62064600 -1.21187900 0.04065500

N -4.27542600 0.00000700 0.24196400

C -5.67703600 0.00001900 0.42027700

C -6.20385200 0.00007400 1.69202600

C -7.56966900 0.00008900 1.86917400

C -8.40608600 0.00004800 0.77540200

C -7.87755500 -0.00000800 -0.49755200

C -6.51340000 -0.00002100 -0.67584900

C -3.62063500 1.21187900 0.04060200

C -4.29736400 2.40729900 0.04421800

C -3.62988200 3.60568200 -0.13436900

C -2.27679100 3.60638400 -0.32387600

C -1.58613100 2.40775700 -0.33464800

C -2.23855100 1.21315700 -0.15181200

C 0.35458900 -0.00010800 -1.62197100

C 1.71168200 -0.00011500 -1.82017800

O 4.83937200 0.00004500 0.07088600

C 8.46914100 -0.00003100 -0.35154700

C 9.62242900 0.00000400 0.39350000

H 10.46989100 0.00010300 2.35727100

H 8.28139800 0.00016500 3.48749400

H 6.20764000 0.00010100 2.15549800

H 2.72714000 0.00012100 1.40059900

H 0.26667400 0.00012600 1.74149100

H -0.51177000 -2.40797900 -0.48067700

H -1.73593300 -4.53604200 -0.46511700

H -4.18898400 -4.53528100 -0.12112300

H -5.37048800 -2.40561400 0.19882200

H -5.52520400 0.00010500 2.53903300

H -7.98467000 0.00013200 2.87206500

H -9.48247500 0.00005900 0.91552400

H -8.53586000 -0.00004000 -1.36051500

H -6.07660500 -0.00006400 -1.66992700

H -5.37046700 2.40563500 0.19870800

H -4.18894400 4.53527600 -0.12134200

H -1.73589600 4.53599800 -0.46535000

H -0.51175200 2.40792300 -0.48081400

H -0.33197300 -0.00017000 -2.46268400

H 2.13642700 -0.00018300 -2.81865800

H 8.49607900 -0.00008600 -1.43632600

H 10.58613300 -0.00002300 -0.10523300

PPZ-DPO_S0_LC-PBE0*

C 9.62574600 -0.00086400 1.74593400

C 8.40031300 -0.00119800 2.39535400

C 7.22543200 -0.00083300 1.66292000

C 7.27877800 -0.00013100 0.27215000

C 6.07007600 0.00026200 -0.52210900

N 5.95801700 0.00087600 -1.80894300

N 4.62031900 0.00102500 -2.08659900

C 4.00616400 0.00048600 -0.95104800

C 2.58078300 0.00038300 -0.70541500

C 2.07403400 -0.00018900 0.59043300

C 0.70463000 -0.00026600 0.79421900

C -0.15898200 0.00021900 -0.29059400

N -1.55736600 0.00011600 -0.07053600

C -2.24827000 -1.21491300 -0.13459300

C -1.59210800 -2.41683300 -0.34522700

C -2.29150500 -3.61966700 -0.36489000

C -3.65745200 -3.61912700 -0.18322700

C -4.32817800 -2.41673900 0.01762000

C -3.64090100 -1.21374600 0.04928900

N -4.29244100 -0.00009400 0.28586300

C -5.70216600 -0.00021300 0.43987700

C -6.25180600 -0.00084000 1.71282800

C -7.63004100 -0.00099100 1.86910200

C -8.45517600 -0.00050700 0.75452600

C -7.90329300 0.00012300 -0.51909400

C -6.52679900 0.00026300 -0.67806000

C -3.64101100 1.21373200 0.04988200

C -4.32838600 2.41668600 0.01886200

C -3.65776600 3.61923400 -0.18137200

C -2.29182100 3.61998400 -0.36304600

C -1.59232100 2.41720000 -0.34400300

C -2.24837900 1.21511300 -0.13400300

C 0.34777400 0.00078100 -1.58664600

C 1.71227600 0.00087000 -1.79665400

O 4.86610400 -0.00002400 0.08898700

C 8.50974500 0.00020500 -0.38117800

C 9.67753400 -0.00016300 0.35796700

H 10.54515000 -0.00115100 2.32312400

H 8.35882600 -0.00174600 3.47990100

H 6.26324900 -0.00109200 2.16480600

H 2.75438100 -0.00056500 1.43567300

H 0.28712500 -0.00070300 1.79576200

H -0.51596200 -2.41375300 -0.48389700

H -1.75225600 -4.54781200 -0.52286000

H -4.21907500 -4.54744700 -0.19436800

H -5.40311000 -2.41206900 0.16440400

H -5.58614600 -0.00120700 2.57025800

H -8.06073300 -0.00148300 2.86557100

H -9.53378300 -0.00062100 0.87797600

H -8.54841300 0.00049900 -1.39224600

H -6.07491300 0.00074400 -1.66564700

H -5.40331200 2.41185200 0.16568600

H -4.21946700 4.54751200 -0.19201600

H -1.75264900 4.54825700 -0.52053200

H -0.51617200 2.41429000 -0.48264800

H -0.34467400 0.00114900 -2.42283900

H 2.12945800 0.00130700 -2.79844900

H 8.52607000 0.00075200 -1.46627900

H 10.63632200 0.00009800 -0.15059700

PXZ-OXD_S0_PBE0

C -8.33887000 0.01419900 -1.56686700

C -7.12478100 0.02156800 -2.24380100

C -5.93129100 0.01477200 -1.53611900

C -5.95023500 0.00048900 -0.13987100

C -4.71980900 -0.00689700 0.62979800

N -4.57698200 -0.01971600 1.91714200

N -3.22975100 -0.02177900 2.16574300

C -2.63792900 -0.01030700 1.01488300

C -1.21307600 -0.00727200 0.73777700

C -0.72906700 0.00484800 -0.57202500

C 0.63787200 0.00724800 -0.80601200

C 1.53043400 -0.00214600 0.26195500

N 2.93009800 0.00033000 0.01808700

C 3.62411200 1.21039500 -0.09705000

C 3.00186000 2.45010400 0.02644400

C 3.73170400 3.62966100 -0.09610300

C 5.09342400 3.58656000 -0.34330500

C 5.72582400 2.35217000 -0.46774900

C 5.00274300 1.18152800 -0.34665800

O 5.69387400 0.00523000 -0.48240900

C 5.00542700 -1.17356600 -0.35489200

C 5.73114400 -2.34167500 -0.48438000

C 5.10144200 -3.57834700 -0.36927500

C 3.73968600 -3.62628800 -0.12317100

C 3.00718300 -2.44929100 0.00782300

C 3.62683100 -1.20732500 -0.10571600

C 1.04824500 -0.01408200 1.56997700

C -0.31525000 -0.01677900 1.80979300

O -3.52445400 -0.00032100 -0.00901300

C -7.17219700 -0.00691300 0.53970500

C -8.35871400 -0.00004200 -0.17501100

H -9.27080700 0.01954700 -2.12346800

H -7.10641900 0.03267800 -3.32891900

H -4.98341500 0.02052700 -2.06320700

H -1.42321800 0.01213400 -1.40509300

H 1.02617700 0.01640400 -1.81907200

H 1.93606500 2.49100400 0.22118500

H 3.22049400 4.58137700 0.00524100

H 5.66945500 4.50053200 -0.43983300

H 6.79055500 2.27447300 -0.66090000

H 6.79573800 -2.26019400 -0.67673000

H 5.67953300 -4.49030400 -0.47233100

H 3.23048200 -4.57985700 -0.02943100

H 1.94130200 -2.49401000 0.20124500

H 1.75478600 -0.02123600 2.39336800

H -0.70387100 -0.02603500 2.82206200

H -7.17053300 -0.01800100 1.62408400

H -9.30550100 -0.00582100 0.35548800

PXZ-OXD_S0_LC-PBE

C -8.31718700 -0.00072800 -1.45335300

C -7.13321700 -0.00082900 -2.15587900

C -5.93301200 -0.00047000 -1.48376900

C -5.92196300 -0.00000700 -0.10422900

C -4.67373100 0.00039000 0.63205100

N -4.49888100 0.00103900 1.89640900

N -3.15668200 0.00119000 2.10629500

C -2.60537100 0.00055300 0.95618800

C -1.19090800 0.00038800 0.63907100

C -0.75631000 0.00007500 -0.66949800

C 0.59247300 -0.00006800 -0.94089700

C 1.50187800 0.00009400 0.09161900

N 2.88344200 -0.00005300 -0.19519000

C 3.59200100 1.19667900 -0.17024200

C 2.99079200 2.41518000 0.05120600

C 3.73534700 3.57772300 0.05297300

C 5.08950600 3.53266700 -0.15849400

C 5.70075000 2.31446800 -0.37506700

C 4.96147100 1.16477700 -0.38691500

O 5.61263500 -0.00032800 -0.64495100

C 4.96129100 -1.16525800 -0.38657400

C 5.70039700 -2.31505600 -0.37439000

C 5.08897000 -3.53309700 -0.15744600

C 3.73480700 -3.57788400 0.05405200

C 2.99042700 -2.41523100 0.05193800

C 3.59181600 -1.19688600 -0.16988800

C 1.06516400 0.00042000 1.40181400

C -0.27819300 0.00056900 1.67767300

O -3.50975700 0.00003200 -0.02975000

C -7.10892900 0.00009400 0.60264000

C -8.30339000 -0.00026600 -0.07446700

H -9.26300500 -0.00101300 -1.98587500

H -7.14366900 -0.00119100 -3.24093800

H -4.99641400 -0.00055000 -2.03140500

H -1.47800600 -0.00005800 -1.47935100

H 0.95906400 -0.00031100 -1.96200200

H 1.91987300 2.45597900 0.21809800

H 3.23749400 4.52627400 0.22430100

H 5.68084100 4.44179400 -0.15692100

H 6.76826700 2.23248700 -0.54934300

H 6.76792500 -2.23329000 -0.54869500

H 5.68016800 -4.44231300 -0.15560700

H 3.23681500 -4.52630800 0.22567900

H 1.91950500 -2.45581700 0.21885900

H 1.79843200 0.00055000 2.20228500

H -0.64487200 0.00082500 2.69889500

H -7.07325300 0.00046400 1.68716800

H -9.23661900 -0.00018800 0.47916400

PXZ-OXD_S0_LC-PBE0*

C -8.32619100 0.66540200 -1.35824300

C -7.12198600 0.99518700 -1.96211600

C -5.92446600 0.67098200 -1.34758700

C -5.93363600 0.01269500 -0.12127400

C -4.70036200 -0.34013400 0.54670100

N -4.54715500 -0.94706300 1.67671100

N -3.20139000 -1.05346200 1.88626600

C -2.62376400 -0.50409400 0.87069000

C -1.20741200 -0.36073200 0.61788400

C -0.74048600 0.25410900 -0.54044600

C 0.62048300 0.37009500 -0.75887800

C 1.52090400 -0.11428200 0.18136900

N 2.90823100 0.01909900 -0.04220700

C 3.52126100 1.26746000 0.08167700

C 2.82253800 2.41391400 0.43786400

C 3.47087900 3.63745800 0.54519400

C 4.82914900 3.73002300 0.30864900

C 5.54029000 2.58608000 -0.02903100

C 4.89693600 1.37240000 -0.13948100

O 5.66414700 0.29259500 -0.47079300

C 5.07138800 -0.93765900 -0.48681700

C 5.88769700 -2.02014500 -0.73369900

C 5.35512100 -3.30069900 -0.80251000

C 3.99632200 -3.47870500 -0.62565100

C 3.17366000 -2.38885200 -0.37200400

C 3.69833200 -1.10542400 -0.28759700

C 1.05352500 -0.72145800 1.34316100

C -0.30391700 -0.85140600 1.55947500

O -3.51622400 -0.02801300 -0.02259300

C -7.14327800 -0.31905100 0.48617300

C -8.33392600 0.00849600 -0.13447100

H -9.26342600 0.92098100 -1.84267400

H -7.11498900 1.50841700 -2.91839400

H -4.97880100 0.92611000 -1.81480300

H -1.44635900 0.63106800 -1.27319100

H 1.00190100 0.83909300 -1.66044000

H 1.75825200 2.34402100 0.63675400

H 2.89910300 4.51711800 0.82184600

H 5.34322800 4.68161900 0.38987100

H 6.60938400 2.61074600 -0.21315700

H 6.94667100 -1.83261600 -0.87817100

H 6.00501600 -4.14656200 -0.99882500

H 3.55827100 -4.46948700 -0.68631100

H 2.10591500 -2.53370800 -0.24560800

H 1.76965400 -1.08980900 2.07120300

H -0.68798300 -1.32170900 2.45883800

H -7.12530800 -0.83218000 1.44223200

H -9.27607500 -0.24972300 0.33832800

PXZ-TAZ_S0_PBE0

C 8.30470200 -0.22195800 -0.13710700

C 7.83984800 -1.50387300 -0.41247600

C 6.48539800 -1.73012000 -0.59886000

C 5.57086000 -0.67415600 -0.51079600

C 4.16124800 -0.98365200 -0.75791800

N 3.76306900 -1.96949000 -1.52711000

N 2.41370900 -1.98291200 -1.53910000

C 1.98271600 -1.00716500 -0.77603700

C 0.56167300 -0.73257500 -0.55648800

C 0.05259900 -0.15657600 0.61116500

C -1.31412800 0.03550800 0.76028200

C -2.19200100 -0.34759900 -0.24734900

N -3.59011700 -0.14422400 -0.08594000

C -4.18886000 1.03204600 -0.54906100

C -3.46978200 2.04971400 -1.17118400

C -4.10660400 3.20753900 -1.61056400

C -5.47174000 3.36315600 -1.43876100

C -6.20141700 2.34998800 -0.82189300

C -5.56983200 1.20346100 -0.38109200

O -6.34954500 0.25816700 0.23346900

C -5.77107800 -0.93766300 0.57528900

C -6.60294800 -1.91563300 1.08433300

C -6.08419800 -3.14825600 1.47263100

C -4.72644100 -3.38540600 1.33928800

C -3.88792500 -2.40182700 0.82123700

C -4.39570500 -1.16471500 0.43257400

C -1.69370600 -0.93545000 -1.40813700

C -0.33080700 -1.12672400 -1.56114200

N 3.06044000 -0.33142000 -0.24818400

C 3.04077600 0.79043600 0.62836400

C 2.57387700 2.01622400 0.16598800

C 2.54866800 3.10609200 1.02642200

C 2.99557900 2.97287500 2.33649700

C 3.46630200 1.74513100 2.78906900

C 3.48692100 0.64796500 1.93782600

C 6.04615400 0.61398600 -0.24829100

C 7.40468200 0.83341600 -0.06084300

H 9.36564400 -0.04567500 0.01107800

H 8.53678600 -2.33332500 -0.48136700

H 6.11194800 -2.72268600 -0.82396800

H 0.71475300 0.12991500 1.41938200

H -1.71197800 0.47850300 1.66743000

H -2.40102400 1.93193200 -1.31010200

H -3.52071100 3.98461100 -2.09072800

H -5.97577200 4.26124700 -1.77927600

H -7.27220400 2.43194600 -0.66777900

H -7.66014400 -1.68871200 1.17324300

H -6.74415700 -3.90969200 1.87415000

H -4.30266200 -4.33938000 1.63553000

H -2.82593800 -2.59416800 0.71846900

H -2.38454300 -1.23789100 -2.18859300

H 0.06682800 -1.59208900 -2.45573500

H 2.23265500 2.10549000 -0.86003500

H 2.18082600 4.06283800 0.67023900

H 2.97704400 3.82710300 3.00559300

H 3.81705100 1.63829300 3.81038100

H 3.84925900 -0.31666700 2.27708000

H 5.36351900 1.45469100 -0.20640200

H 7.76048600 1.83957200 0.13819200

PXZ-TAZ_S0_LC-PBE

C 8.21051800 -0.20759300 -0.16517900

C 7.75720400 -1.48887700 -0.39227400

C 6.41598300 -1.72304300 -0.57515200

C 5.51288100 -0.67689000 -0.52915700

C 4.10751700 -0.99375500 -0.76562300

N 3.70630700 -1.97001700 -1.51228300

N 2.36633200 -1.98133400 -1.50561700

C 1.95629500 -1.01515300 -0.75181900

C 0.54583700 -0.72649200 -0.50896200

C 0.07543100 -0.18005900 0.66935100

C -1.27392200 0.02956800 0.84235500

C -2.15997500 -0.30851500 -0.15362200

N -3.54191800 -0.08955900 0.03462800

C -4.13994400 1.04754200 -0.49461700

C -3.43121300 2.02247400 -1.16001500

C -4.06854100 3.14145700 -1.65791500

C -5.42242700 3.29363300 -1.50269900

C -6.14144800 2.31805100 -0.84218000

C -5.50845100 1.21533100 -0.34057600

O -6.26313600 0.30877000 0.33468600

C -5.71606600 -0.90891900 0.59479800

C -6.55453900 -1.90261200 1.01642500

C -6.05246100 -3.14839600 1.33369200

C -4.70641500 -3.38153000 1.21360400

C -3.86151500 -2.38049500 0.77781600

C -4.35358800 -1.13325300 0.46543900

C -1.69665400 -0.86835700 -1.32652900

C -0.35204200 -1.07709700 -1.50244700

N 3.02842500 -0.34458700 -0.24933500

C 3.02274900 0.77359400 0.61602000

C 2.50084000 1.96834600 0.17588700

C 2.48842200 3.05182400 1.02360700

C 3.00458200 2.93953200 2.29598100

C 3.53116100 1.74082700 2.72459100

C 3.53762200 0.65016900 1.88600900

C 5.97223900 0.60884400 -0.31605800

C 7.31690000 0.83854000 -0.13300100

H 9.27016800 -0.02238700 -0.01968900

H 8.45797200 -2.31681700 -0.42846500

H 6.04115500 -2.72220800 -0.77001300

H 0.75827500 0.07393200 1.47247900

H -1.65802800 0.45439600 1.76396100

H -2.36034700 1.90447800 -1.28477500

H -3.48711500 3.89771700 -2.17482200

H -5.93014500 4.16894600 -1.89294700

H -7.21308700 2.39813000 -0.69418900

H -7.61153900 -1.67313400 1.09900900

H -6.72199500 -3.93090300 1.67376500

H -4.29349900 -4.35447700 1.45826900

H -2.79759500 -2.56925000 0.68381100

H -2.40796800 -1.13881000 -2.10088600

H 0.03125100 -1.52982200 -2.41055400

H 2.10282700 2.03778300 -0.83143300

H 2.07324000 3.99529800 0.68491700

H 2.99652600 3.79669000 2.96142600

H 3.93981700 1.65157400 3.72570900

H 3.94643700 -0.30291000 2.20580600

H 5.28275900 1.44612800 -0.30681600

H 7.67008700 1.85180300 0.02970600

PXZ-TAZ_S0_LC-PBE0*

C 8.26112600 -0.25654000 -0.15765800

C 7.80361200 -1.55268700 -0.34877800

C 6.45134200 -1.79751400 -0.49701300

C 5.53598800 -0.74573400 -0.45491500

C 4.13079200 -1.08141400 -0.65277400

N 3.72288500 -2.14073200 -1.30432200

N 2.37730600 -2.15133800 -1.29602500

C 1.96143400 -1.09967700 -0.63859600

C 0.55251700 -0.79129700 -0.42615300

C 0.07703100 -0.04728500 0.65227500

C -1.28294800 0.16650600 0.80392800

C -2.17932800 -0.35500200 -0.11546800

N -3.56444700 -0.11376700 0.04190700

C -4.12090500 1.06095200 -0.46274300

C -3.35996700 2.04599800 -1.07748700

C -3.95457500 3.20767200 -1.55283500

C -5.31809500 3.39368900 -1.43079300

C -6.08965100 2.40636800 -0.83152300

C -5.49940400 1.25831600 -0.35120000

O -6.31242700 0.33972400 0.24883500

C -5.78759900 -0.89283400 0.51920800

C -6.66571100 -1.87926000 0.91023800

C -6.19646300 -3.14252500 1.24599800

C -4.84100300 -3.40169200 1.18010900

C -3.95602700 -2.41052500 0.77627100

C -4.41607400 -1.14538400 0.43710300

C -1.71252800 -1.10522800 -1.18849300

C -0.35790200 -1.32621000 -1.33937100

N 3.04292200 -0.37882700 -0.20447700

C 3.03594700 0.82550200 0.54105400

C 2.54835800 1.98537400 -0.04131900

C 2.53379300 3.15916000 0.69413900

C 3.01248400 3.17109800 1.99619700

C 3.50442300 2.00640500 2.56726100

C 3.51404500 0.82602400 1.84231700

C 6.00101600 0.55625000 -0.27865300

C 7.35776800 0.79375100 -0.12843200

H 9.32320100 -0.06478100 -0.03848800

H 8.50627900 -2.37966900 -0.38134000

H 6.07458200 -2.80228900 -0.65819300

H 0.76119800 0.35139500 1.39375800

H -1.66508700 0.73704800 1.64471400

H -2.28975700 1.89656900 -1.17936100

H -3.33691800 3.96498600 -2.02492100

H -5.78999800 4.29774900 -1.80072400

H -7.16424100 2.50929200 -0.71963700

H -7.72113000 -1.63036000 0.95492700

H -6.89386000 -3.91250800 1.55838900

H -4.45468000 -4.38092700 1.44367600

H -2.89114700 -2.61506300 0.73071500

H -2.42482100 -1.51185600 -1.90038900

H 0.02601800 -1.91969200 -2.16258600

H 2.18133300 1.95485000 -1.06258900

H 2.14884200 4.06967200 0.24623500

H 3.00227900 4.09311500 2.56882800

H 3.88094000 2.01481500 3.58508600

H 3.89395700 -0.09622800 2.27070200

H 5.31040900 1.39323200 -0.27497600

H 7.71038600 1.81196800 0.00453000

PXZ-TRZ_S0_PBE0

C 5.89871000 -4.82154200 -0.01612700

C 6.59252000 -3.61584800 -0.01206300

C 5.89981400 -2.41464500 -0.00805000

C 4.50280900 -2.40769900 -0.00806000

C 3.76428300 -1.12959100 -0.00378300

N 4.47262300 0.00023600 -0.00001000

C 3.76398200 1.12987400 0.00376400

C 4.50216700 2.40817900 0.00805300

C 5.89917000 2.41549300 0.00806700

C 6.59155800 3.61688000 0.01209100

C 5.89743000 4.82239000 0.01614000

C 4.50639400 4.82207900 0.01614800

C 3.81114900 3.62230700 0.01212900

N 2.43002600 1.17973100 0.00396600

C 1.81108300 -0.00012100 0.00001400

C 0.33214300 -0.00032100 0.00001000

C -0.37331700 1.20488300 0.00456900

C -1.75943600 1.20495600 0.00465100

C -2.45650100 -0.00069800 0.00000200

N -3.87832700 -0.00090300 -0.00000400

C -4.58350600 -0.00944900 1.20833300

C -3.95123400 -0.01874600 2.44916700

C -4.69320000 -0.02712800 3.62748100

C -6.07713000 -0.02626800 3.58240700

C -6.71960900 -0.01683100 2.34690200

C -5.98462700 -0.00848600 1.17751400

O -6.68707400 0.00083700 -0.00000200

C -5.98461800 0.00912100 -1.17752000

C -6.71959100 0.01814200 -2.34690800

C -6.07710300 0.02674100 -3.58241500

C -4.69317200 0.02605900 -3.62748900

C -3.95121500 0.01693900 -2.44917500

C -4.58349700 0.00848100 -1.20834000

C -1.75911200 -1.20615800 -0.00464300

C -0.37298500 -1.20571200 -0.00455400

N 2.43034200 -1.17980700 -0.00395900

C 3.81211200 -3.62201000 -0.01214900

C 4.50767500 -4.82159900 -0.01615900

H 6.44230600 -5.76154900 -0.01926700

H 7.67807700 -3.61310200 -0.01202500

H 6.42705100 -1.46797500 -0.00486100

H 6.42665800 1.46896300 0.00488700

H 7.67711600 3.61442100 0.01207200

H 6.44077700 5.76254100 0.01928800

H 3.96229500 5.76142300 0.01930000

H 2.72766300 3.60748100 0.01207600

H 0.18102700 2.13580100 0.00815500

H -2.31575300 2.13666000 0.00828900

H -2.86785700 -0.01935900 2.49118300

H -4.17350100 -0.03428300 4.58000700

H -6.66259400 -0.03277100 4.49549200

H -7.80161100 -0.01568900 2.26739400

H -7.80159400 0.01825700 -2.26739900

H -6.66256000 0.03381300 -4.49550000

H -4.17346700 0.03255600 -4.58001700

H -2.86783800 0.01633700 -2.49119200

H -2.31518800 -2.13800600 -0.00828400

H 0.18160800 -2.13648200 -0.00813600

H 2.72862300 -3.60747100 -0.01211400

H 3.96382400 -5.76108600 -0.01932300

PXZ-TRZ_S0_LC-PBE

C 5.84283800 -4.77674000 -0.01254300

C 6.53159400 -3.58336500 -0.00941500

C 5.84418800 -2.39320400 -0.00630800

C 4.46335800 -2.39165400 -0.00631700

C 3.72781200 -1.11884600 -0.00299000

N 4.42502900 0.00016800 -0.00002100

C 3.72758900 1.11904300 0.00293300

C 4.46288100 2.39199700 0.00630000

C 5.84371000 2.39382100 0.00633600

C 6.53088100 3.58411800 0.00948000

C 5.84188800 4.77735600 0.01260000

C 4.46404300 4.77909000 0.01257200

C 3.77550200 3.58956300 0.00943000

N 2.40876600 1.16460900 0.00305500

C 1.79311100 -0.00009500 -0.00004400

C 0.32053400 -0.00024300 -0.00003300

C -0.37164200 1.19434000 0.00352500

C -1.74549100 1.19446800 0.00361200

C -2.43250500 -0.00051800 -0.00001400

N -3.84331500 -0.00066500 -0.00000300

C -4.54231600 -0.00734300 1.19936700

C -3.91323600 -0.01445700 2.42402400

C -4.64610800 -0.02100900 3.59402100

C -6.01646900 -0.02045900 3.55134900

C -6.65496300 -0.01320800 2.32768500

C -5.92946200 -0.00670400 1.16924000

O -6.62249200 0.00044500 0.00002700

C -5.92948100 0.00703500 -1.16920000

C -6.65500200 0.01406100 -2.32763000

C -6.01652900 0.02087000 -3.55130700

C -4.64616900 0.02044700 -3.59400800

C -3.91327800 0.01333700 -2.42402600

C -4.54233600 0.00664900 -1.19935600

C -1.74525400 -1.19536200 -0.00364900

C -0.37139900 -1.19496100 -0.00358100

N 2.40899900 -1.16467600 -0.00312400

C 3.77621700 -3.58935700 -0.00945400

C 4.46499400 -4.77874700 -0.01256000

H 6.38642300 -5.71658300 -0.01499600

H 7.61686400 -3.58136100 -0.00939900

H 6.36651500 -1.44324500 -0.00381900

H 6.36622700 1.44396600 0.00385100

H 7.61615100 3.58233000 0.00950000

H 6.38528500 5.71730800 0.01508200

H 3.92092000 5.71867200 0.01502700

H 2.69162700 3.56908200 0.00934500

H 0.18867100 2.12228800 0.00630900

H -2.30648300 2.12356200 0.00647000

H -2.82920300 -0.01481300 2.46116700

H -4.12579100 -0.02655200 4.54598200

H -6.59968400 -0.02558800 4.46569300

H -7.73672700 -0.01239300 2.24670800

H -7.73676500 0.01404500 -2.24663000

H -6.59975900 0.02643100 -4.46563900

H -4.12586800 0.02564400 -4.54597900

H -2.82924500 0.01291400 -2.46119200

H -2.30607100 -2.12456000 -0.00650000

H 0.18909800 -2.12279700 -0.00637200

H 2.69233700 -3.56909000 -0.00940400

H 3.92205700 -5.71843700 -0.01502100

PXZ-TRZ_S0_LC-PBE0*

C 5.87866900 -4.68576100 -1.02189800

C 6.57066600 -3.51082200 -0.76547600

C 5.87818600 -2.34110500 -0.51022400

C 4.48656900 -2.34076300 -0.51016000

C 3.74960500 -1.09833000 -0.23930900

N 4.45600300 0.00196300 0.00020600

C 3.74664500 1.10062200 0.23846700

C 4.48026700 2.34475100 0.51060800

C 5.87187600 2.34825700 0.51328200

C 6.56121600 3.51955700 0.76977300

C 5.86607100 4.69293500 1.02482300

C 4.47861000 4.69327500 1.02299000

C 3.78647300 3.52353600 0.76682200

N 2.41732400 1.14991100 0.24692100

C 1.80244100 -0.00109500 -0.00216000

C 0.33151300 -0.00282300 -0.00335200

C -0.36964800 1.17075700 0.25650100

C -1.75146400 1.16849700 0.26169300

C -2.44455100 -0.00607100 -0.00572200

N -3.85583400 -0.00863600 -0.00607100

C -4.56012400 -0.66154800 1.00599400

C -3.93021300 -1.30494000 2.06307200

C -4.66936800 -1.94180500 3.05069600

C -6.04970400 -1.93755900 3.00052500

C -6.68941900 -1.28188400 1.95771700

C -5.95663800 -0.64980700 0.97733200

O -6.65934800 -0.01131300 -0.00302300

C -5.95988800 0.64051000 -0.97693700

C -6.69610200 1.28165400 -1.94882800

C -6.06010600 1.94726800 -2.98759400

C -4.67993700 1.95312200 -3.04184700

C -3.93728500 1.30824100 -2.06208100

C -4.56342000 0.65412100 -1.00932800

C -1.74827700 -1.17901800 -0.27165900

C -0.36645900 -1.17805500 -0.26424700

N 2.42041900 -1.15067900 -0.25013100

C 3.79593900 -3.52110600 -0.76774200

C 4.49121200 -4.68925300 -1.02267800

H 6.42359300 -5.60368100 -1.22228500

H 7.65640800 -3.50819100 -0.76491200

H 6.39777600 -1.41066300 -0.30721800

H 6.39396000 1.41898900 0.31130200

H 7.64696100 3.51939300 0.77125400

H 6.40852900 5.61210100 1.22618200

H 3.93465900 5.61145900 1.22270600

H 2.70190400 3.50026400 0.76033400

H 0.19066400 2.07626100 0.46349100

H -2.31165700 2.07483600 0.47174600

H -2.84624300 -1.30073500 2.11369400

H -4.14938500 -2.43823300 3.86368400

H -6.63501500 -2.43379800 3.76739600

H -7.77159600 -1.24276100 1.88423800

H -7.77802100 1.24062100 -1.87264500

H -6.64813500 2.44986600 -3.74821800

H -4.16278700 2.45718500 -3.85193900

H -2.85348900 1.30536600 -2.11617100

H -2.30619700 -2.08659700 -0.48232200

H 0.19630500 -2.08228000 -0.47015900

H 2.71130900 -3.50030100 -0.76328600

H 3.94972500 -5.60865800 -1.22347000

CC2TA_S1_PBE0_TDA

C -1.11254700 2.49036700 0.06676300

N -0.55074700 3.61549100 -0.24771900

C 0.80722000 3.70242500 -0.10155500

N 1.53602800 2.61466900 0.38070000

C 0.82568000 1.56598400 0.61173700

N -0.48149200 1.36366500 0.50320900

C 1.48504500 4.89836700 -0.44655300

C 0.79811900 5.99535800 -1.03235400

C 1.46499800 7.15618800 -1.36240200

C 2.83746600 7.29272000 -1.12908600

C 3.52793800 6.22375100 -0.55208000

C 2.87850400 5.05246400 -0.21870000

N 1.55508700 0.38922800 1.05727900

N -2.50884200 2.39406600 -0.03141800

C -3.25418100 1.21278000 -0.12455000

C -4.62327700 1.54667600 -0.19798500

C -4.71555600 2.98184500 -0.14234800

C -3.40021300 3.47788000 -0.04817700

C 2.79798800 0.04405400 0.64033900

C 3.17238500 -1.18968800 1.26066500

C 2.07823200 -1.56635200 2.11603800

C 1.09009100 -0.57384000 1.95890700

C -2.85082700 -0.12148300 -0.18924500

C -3.82729600 -1.09858000 -0.29902500

C -5.18754900 -0.77267100 -0.36806100

C -5.58962600 0.55361500 -0.32673100

C -5.80167600 3.85468900 -0.15193700

C -5.56753800 5.21557400 -0.05928100

C -4.25903900 5.69735700 0.04648100

C -3.16204800 4.84807500 0.05215700

C 1.86826000 -2.63880100 2.97899600

C 0.67330000 -2.70501000 3.67885900

C -0.29653400 -1.71178300 3.51652500

C -0.10959300 -0.63353300 2.66119900

C 3.66349500 0.69365500 -0.25938500

C 4.88311500 0.11744200 -0.51431400

C 5.25927200 -1.10224400 0.09517700

C 4.39612400 -1.76011500 0.98221900

N -6.15294900 -1.80562400 -0.47902300

N 6.50717400 -1.65706600 -0.19041200

C -6.25760800 -2.70574400 -1.52707900

C -7.32746700 -3.59240300 -1.27050700

C -7.89296100 -3.20045400 -0.00367600

C -7.13833200 -2.09584400 0.45130600

C 7.71806600 -0.96513700 -0.22658400

C 8.75187400 -1.87067500 -0.54218300

C 8.13460500 -3.17249600 -0.70362800

C 6.75299600 -2.99894600 -0.48077600

C -5.50069900 -2.79092200 -2.69309000

C -5.82456000 -3.78970600 -3.59846600

C -6.87589600 -4.68311500 -3.35712600

C -7.63078800 -4.58764600 -2.19916100

C -8.94484500 -3.68025500 0.77630400

C -9.22487700 -3.06370400 1.98510300

C -8.46056800 -1.97515500 2.42392000

C -7.40991700 -1.47679800 1.66894900

C 8.62174100 -4.42358500 -1.04428900

C 7.72417300 -5.48303500 -1.16458500

C 6.35525800 -5.28963300 -0.97457900

C 5.84452400 -4.04220400 -0.64185600

C 7.97488400 0.36949900 0.07745300

C 9.29256500 0.79968400 0.00146700

C 10.32164800 -0.07824100 -0.34174900

C 10.06099300 -1.42219900 -0.60272100

H -0.26208200 5.89508200 -1.23508900

H 0.91176300 7.97526600 -1.81456700

H 3.35438300 8.21042500 -1.38933200

H 4.59323200 6.31600900 -0.35535800

H 3.41958400 4.23833500 0.24968300

H -1.80488900 -0.38277000 -0.13218600

H -3.53878700 -2.14451700 -0.32546900

H -6.64319200 0.80290400 -0.40523600

H -6.81482400 3.47042300 -0.22679300

H -6.40016900 5.91199800 -0.06706600

H -4.08908200 6.76723300 0.12200100

H -2.15401700 5.22844800 0.12039200

H 2.62551000 -3.40682500 3.10686100

H 0.48869800 -3.53083700 4.35773500

H -1.22718700 -1.78168500 4.07025000

H -0.86991300 0.12125500 2.52093900

H 3.36603700 1.62312900 -0.72179800

H 5.54814300 0.57159000 -1.23983200

H 4.71981900 -2.66547300 1.48388600

H -4.69164300 -2.09489300 -2.88719000

H -5.25149800 -3.87636000 -4.51683400

H -7.10226500 -5.45323200 -4.08790800

H -8.45080600 -5.27692800 -2.01885100

H -9.53622800 -4.52761900 0.44118500

H -10.04250700 -3.42620900 2.60022800

H -8.69383700 -1.50944300 3.37678500

H -6.81766700 -0.63599800 2.01392800

H 9.68000800 -4.57782400 -1.22707000

H 8.09214400 -6.46829000 -1.42920400

H 5.67341500 -6.12298000 -1.10253200

H 4.77750000 -3.88810200 -0.53771700

H 7.18591400 1.04648500 0.38059200

H 9.52347100 1.83511300 0.22499100

H 11.34144500 0.28766700 -0.38876300

H 10.87244800 -2.10310000 -0.83740700

CC2TA_S1_LC-PBE_TDA

C -1.16800700 2.34349800 0.03001200

N -0.58987500 3.47727100 -0.32117900

C 0.72454300 3.47237600 -0.29373700

N 1.46182400 2.42612000 0.06451800

C 0.78285200 1.34698000 0.39890300

N -0.52779700 1.23645500 0.39512700

C 1.42052200 4.70244900 -0.67606200

C 0.72958800 5.73107700 -1.29017900

C 1.38563200 6.88279000 -1.65160400

C 2.73359400 7.01912100 -1.39637500

C 3.42464000 5.99793600 -0.78106400

C 2.77244600 4.84150300 -0.42487600

N 1.50469700 0.24215100 0.77923400

N -2.54202400 2.29481200 0.00415100

C -3.30487400 1.15673500 -0.05172400

C -4.68155300 1.53961000 -0.06235000

C -4.72361100 2.95406000 -0.01802400

C -3.40148000 3.41625000 0.03059700

C 2.84846200 0.00236700 0.48513100

C 3.19905000 -1.24068800 0.99954100

C 2.03620600 -1.77932500 1.65000200

C 1.01276100 -0.85206000 1.49835500

C -2.93374400 -0.17882600 -0.15303200

C -3.91371600 -1.13485900 -0.25789300

C -5.27481500 -0.76274500 -0.23697000

C -5.65143100 0.57058600 -0.14220800

C -5.76408100 3.86598700 0.00677800

C -5.47343900 5.20745400 0.10191400

C -4.16417100 5.64699000 0.17760500

C -3.10907900 4.75282900 0.14440700

C 1.82598600 -2.95151500 2.34532500

C 0.59089700 -3.18247100 2.89510500

C -0.41734500 -2.24223400 2.76041400

C -0.22680000 -1.06999600 2.06914000

C 3.76483300 0.75226700 -0.22805800

C 5.02429300 0.23668200 -0.40146700

C 5.38714500 -0.99130400 0.13326400

C 4.47404200 -1.73575200 0.83908300

N -6.24567200 -1.73706000 -0.32333500

N 6.69071300 -1.47484300 -0.05782600

C -6.22971300 -2.82946500 -1.17987500

C -7.38322700 -3.58071700 -0.97875200

C -8.13914900 -2.91873100 0.05258900

C -7.40792600 -1.79756100 0.43213600

C 7.84964000 -0.82755600 0.30668100

C 8.94265100 -1.61579100 -0.04382300

C 8.41306800 -2.80601100 -0.65178000

C 7.02662900 -2.67586500 -0.64075300

C -5.31807900 -3.15629800 -2.16399600

C -5.56336200 -4.28433800 -2.90802700

C -6.69254500 -5.05996000 -2.69648300

C -7.61242200 -4.70690500 -1.73950600

C -9.33184700 -3.20698000 0.68067100

C -9.77008500 -2.37998300 1.68584500

C -9.01334100 -1.28715200 2.07918700

C -7.82127900 -0.98392500 1.46792100

C 8.99010900 -3.93588400 -1.20000200

C 8.18038400 -4.90795900 -1.72823100

C 6.79960100 -4.75640800 -1.72056700

C 6.20339800 -3.64501300 -1.18204500

C 8.00695800 0.38765600 0.94570600

C 9.28623000 0.80430700 1.21096000

C 10.38826900 0.03633200 0.85669900

C 10.22233600 -1.17377600 0.23412600

H -0.32895600 5.60395300 -1.49129600

H 0.84092100 7.68402800 -2.14084600

H 3.25056600 7.93052500 -1.68042400

H 4.48491400 6.10555700 -0.57549400

H 3.29838500 4.03090200 0.06805200

H -1.89107400 -0.46064500 -0.12025300

H -3.64967600 -2.18504400 -0.25767000

H -6.70235500 0.83307700 -0.20224100

H -6.79453400 3.52805300 -0.04407300

H -6.28308100 5.93013700 0.12219700

H -3.95422200 6.70746000 0.26492500

H -2.08520700 5.09357400 0.19944500

H 2.62840600 -3.67390100 2.46015800

H 0.40381300 -4.09837400 3.44596100

H -1.38609300 -2.42882700 3.21304300

H -1.01877500 -0.34064100 1.97884600

H 3.49016300 1.71057800 -0.64359600

H 5.75884300 0.79024800 -0.97775500

H 4.76631600 -2.69200700 1.26194600

H -4.44877800 -2.53868300 -2.35735900

H -4.85981200 -4.56707700 -3.68444400

H -6.85619400 -5.94411700 -3.30358600

H -8.50987300 -5.29958400 -1.59111800

H -9.91044600 -4.07900100 0.39176200

H -10.70736800 -2.59132900 2.18976200

H -9.36211000 -0.66242500 2.89514400

H -7.21933500 -0.14899900 1.80784200

H 10.06998800 -4.04991100 -1.21468900

H 8.61819700 -5.80118100 -2.16157300

H 6.17692300 -5.53297100 -2.15393500

H 5.12514500 -3.52514400 -1.19038900

H 7.14870300 0.98487300 1.23515000

H 9.43960100 1.75460100 1.71294700

H 11.38647600 0.39762600 1.08111800

H 11.08358000 -1.77844700 -0.03417200

CC2TA_S1_LC-PBE0*_TDA

C -1.09953300 2.43167100 0.00371400

N -0.50771800 3.55589200 -0.23589900

C 0.84955800 3.59119300 -0.09418000

N 1.54378000 2.46675000 0.32547000

C 0.80654000 1.42735400 0.48574200

N -0.49883800 1.26058200 0.35855000

C 1.56783800 4.77567900 -0.38421700

C 0.91922300 5.91383900 -0.91366600

C 1.62655700 7.05912500 -1.19925500

C 3.00128800 7.13002400 -0.97206300

C 3.65245800 6.01845200 -0.44719800

C 2.95972600 4.86114600 -0.15869100

N 1.50580100 0.21726700 0.85524500

N -2.48886100 2.37716900 -0.08586000

C -3.26644600 1.22029300 -0.15557600

C -4.62296800 1.59197500 -0.18337600

C -4.67166300 3.02664700 -0.13112600

C -3.34439300 3.48327400 -0.07920700

C 2.77629500 -0.06390700 0.51256300

C 3.12553300 -1.34929500 1.03473200

C 1.96842500 -1.82751700 1.74398800

C 0.97987000 -0.83977600 1.60620500

C -2.90393700 -0.12144200 -0.23776500

C -3.90966100 -1.06722500 -0.31118200

C -5.25772100 -0.70221300 -0.33152900

C -5.61999900 0.63187500 -0.27876700

C -5.72967800 3.92651700 -0.11565600

C -5.45295900 5.27745500 -0.04102900

C -4.13176800 5.72028300 0.02121700

C -3.06159900 4.84263600 0.00169400

C 1.70936400 -2.98390800 2.46164400

C 0.45778600 -3.13871700 3.03833200

C -0.51040300 -2.14983600 2.89739600

C -0.27129000 -0.98209500 2.18372600

C 3.69685300 0.68892000 -0.24270300

C 4.94461000 0.16570600 -0.43451500

C 5.30218500 -1.09779400 0.08876100

C 4.37747100 -1.86176400 0.82082100

N -6.24826700 -1.70130100 -0.40871500

N 6.57088800 -1.58561400 -0.12388500

C -6.32557600 -2.68563800 -1.37466900

C -7.43764500 -3.50729200 -1.10715900

C -8.05926000 -2.98405700 0.07995200

C -7.29321500 -1.87154300 0.47889500

C 7.75330800 -0.84155000 -0.07228600

C 8.83489900 -1.69619500 -0.33504400

C 8.28918800 -3.01473100 -0.55940600

C 6.89666500 -2.91256000 -0.42242400

C -5.51126900 -2.89553100 -2.48057900

C -5.82142600 -3.95754300 -3.31038700

C -6.91318900 -4.79066400 -3.05381200

C -7.72606500 -4.56855200 -1.95809100

C -9.16783400 -3.35801700 0.83141600

C -9.49091200 -2.62875200 1.96030700

C -8.71275600 -1.53494500 2.34742200

C -7.60603200 -1.14021000 1.61792900

C 8.86068800 -4.23094500 -0.89490000

C 8.03296200 -5.32335400 -1.09983400

C 6.64980300 -5.19722300 -1.00056100

C 6.05754300 -3.98840000 -0.67239700

C 7.94092600 0.48785800 0.27766100

C 9.24166600 0.96430300 0.29703500

C 10.32185800 0.13731600 0.00049900

C 10.12764900 -1.20105400 -0.30324400

H -0.14673800 5.85697700 -1.11006200

H 1.10383900 7.91808700 -1.61157700

H 3.55167200 8.03730600 -1.19851800

H 4.72149600 6.06220100 -0.25508100

H 3.46676200 4.00636000 0.27670100

H -1.86207900 -0.40951500 -0.23053500

H -3.65472000 -2.12226700 -0.34779500

H -6.66842600 0.91208500 -0.32856300

H -6.75509100 3.56994200 -0.15864900

H -6.26436700 5.99845100 -0.02965800

H -3.92956400 6.78549000 0.08268900

H -2.03797800 5.18772400 0.03967000

H 2.46923000 -3.75180100 2.57443300

H 0.23172200 -4.03564700 3.60508100

H -1.48509900 -2.28885200 3.35361100

H -1.02697600 -0.21812800 2.05779100

H 3.40286300 1.64799400 -0.64578900

H 5.65855400 0.69274900 -1.05718700

H 4.68598800 -2.80282200 1.26360400

H -4.67047200 -2.24140700 -2.68894900

H -5.20345900 -4.14319400 -4.18387300

H -7.12641000 -5.61536000 -3.72666600

H -8.58131300 -5.21062900 -1.76617600

H -9.76953700 -4.21306300 0.53580000

H -10.35511200 -2.90746900 2.55507400

H -8.98064700 -0.98053200 3.24193200

H -6.99871700 -0.29451500 1.92501100

H 9.93628500 -4.32382900 -1.00844600

H 8.46428400 -6.28363300 -1.36184100

H 6.01835400 -6.05719400 -1.19728200

H 4.97800400 -3.89169800 -0.64694900

H 7.11329500 1.13194700 0.55163000

H 9.41779500 2.00190500 0.55966100

H 11.32770600 0.54270100 0.02642900

H 10.97414100 -1.85136200 -0.50079900

DMOC-DPS_S1_PBE0_TDA

C 4.97641900 6.15779800 -1.00955800

O 3.84209100 5.32227500 -0.87193900

C 4.01072200 4.01132000 -0.69056000

C 2.81458200 3.26753200 -0.56522500

C 2.83900600 1.90925000 -0.37469300

C 4.09216300 1.28427200 -0.30830300

C 5.30054000 2.01765900 -0.43328600

C 5.26888100 3.38350000 -0.62472400

C 6.37057300 1.04019900 -0.31654300

C 7.74202800 1.10701200 -0.35165700

C 8.48304000 -0.08714300 -0.19917500

O 9.81028100 0.07153400 -0.24645700

C 10.64323200 -1.06470900 -0.10433700

C 7.83777000 -1.31941700 -0.01500800

C 6.45410700 -1.39035000 0.02112100

C 5.73162100 -0.21126500 -0.12992400

N 4.36984400 -0.03812500 -0.12831400

C 3.40039700 -1.08419600 0.03545400

C 2.92253200 -1.77962600 -1.09777400

C 2.02302800 -2.80137400 -0.94161800

C 1.58206500 -3.17854800 0.35963600

S 0.25577600 -4.27035200 0.53569500

O 0.28904900 -4.81890900 1.89322000

O 0.21696600 -5.13712800 -0.64418200

C -1.20281800 -3.25529400 0.45137600

C -1.74882200 -2.92620900 -0.79235100

C -2.77987200 -2.00582700 -0.87108800

C -3.28709100 -1.41602100 0.28929700

N -4.34041600 -0.47532600 0.20735400

C -4.30027700 0.82975700 0.67314600

C -3.26539400 1.51903900 1.30515900

C -3.48147900 2.83572400 1.66009800

C -4.70406700 3.47875100 1.39452200

O -4.77210800 4.77664800 1.80604200

C -5.97408300 5.46428400 1.58181500

C -5.73070500 2.80035600 0.75665000

C -5.52605000 1.46445500 0.39053400

C -6.34672400 0.49339800 -0.28184900

C -7.65172500 0.53139600 -0.78865400

C -8.16852700 -0.61315600 -1.37473800

O -9.41762000 -0.71253900 -1.90872200

C -10.23805700 0.42377600 -1.85485800

C -7.39735200 -1.78793600 -1.44810600

C -6.11038500 -1.84009500 -0.95269000

C -5.58219200 -0.68579400 -0.37404100

C -2.75359100 -1.76435700 1.53221200

C -1.70820400 -2.66853900 1.61450000

C 2.07557800 -2.47022600 1.49324500

C 2.97383800 -1.44805700 1.33247700

H 5.59974700 6.12665100 -0.11106300

H 5.56803900 5.87729000 -1.88597500

H 4.58526600 7.16320900 -1.14380800

H 1.87863500 3.81011700 -0.62592500

H 1.93126200 1.32679700 -0.27841800

H 6.18570900 3.95213600 -0.72143500

H 8.28692100 2.03429100 -0.49120000

H 11.66247700 -0.69271900 -0.17577900

H 10.46131300 -1.78810500 -0.90434700

H 10.49554900 -1.54125300 0.86906800

H 8.41304600 -2.22878400 0.10101300

H 5.93898800 -2.33270200 0.16241400

H 3.28021100 -1.51327500 -2.08817500

H 1.66685200 -3.35843100 -1.80041500

H -1.36652600 -3.40924500 -1.68471000

H -3.20204000 -1.73498600 -1.83382900

H -2.31648500 1.03705100 1.51440400

H -2.70848000 3.41025200 2.15965900

H -5.83129600 6.46588900 1.98606700

H -6.81694400 4.98649900 2.09662900

H -6.20599900 5.53931300 0.51190400

H -6.67724200 3.28079000 0.53657800

H -8.23440400 1.44234200 -0.71226000

H -11.17905700 0.14150900 -2.32565800

H -10.43349300 0.73469300 -0.82087000

H -9.80060200 1.26677400 -2.40453600

H -7.85060700 -2.66066600 -1.90600400

H -5.53382800 -2.75708200 -1.00682700

H -3.18096100 -1.33482100 2.43293300

H -1.30237500 -2.96335900 2.57579100

H 1.75964400 -2.77531200 2.48417100

H 3.37075600 -0.92814700 2.19963400

DMOC-DPS_S1_LC-PBE_TDA

C 6.63727400 5.82464800 -0.01854600

O 5.42918900 5.27889700 0.41585500

C 5.25005700 3.95981900 0.33074500

C 4.01172600 3.50360400 0.78053500

C 3.71537500 2.14792000 0.74407000

C 4.67938500 1.29181000 0.26711600

C 5.93213600 1.74393300 -0.22330400

C 6.22295900 3.09870300 -0.17688400

C 6.63728800 0.61145300 -0.69035900

C 7.88356700 0.41833500 -1.25255400

C 8.28670200 -0.88411500 -1.56286200

O 9.50732900 -0.97110100 -2.10032100

C 9.99704600 -2.23217400 -2.43632400

C 7.48115200 -1.98563700 -1.32101100

C 6.21448800 -1.79472100 -0.75563300

C 5.81167000 -0.52003400 -0.47495800

N 4.64120400 -0.06911000 0.10170600

C 3.56848500 -0.88582000 0.46877500

C 3.05467600 -1.78920200 -0.44151500

C 2.00099600 -2.59099200 -0.08053500

C 1.47373600 -2.47812200 1.18525700

S 0.11912200 -3.49808900 1.64357900

O 0.13088000 -3.59839700 3.07958700

O 0.15173800 -4.65530100 0.78769600

C -1.28801600 -2.55480800 1.19486600

C -1.82421400 -2.70213000 -0.06356400

C -2.91653800 -1.95128700 -0.41945000

C -3.47688600 -1.07143100 0.48842400

N -4.59027000 -0.31662600 0.12972800

C -4.74300500 1.04500600 0.31532900

C -3.85736400 1.97652200 0.83282600

C -4.25149400 3.28174000 0.87593300

C -5.50358700 3.68379500 0.40837300

O -5.75994900 5.00035600 0.51889000

C -6.99122000 5.45554500 0.06049500

C -6.37098200 2.76757500 -0.12529700

C -5.97874800 1.43650500 -0.17337300

C -6.61691400 0.25558300 -0.68178600

C -7.85931100 0.03732300 -1.26221900

C -8.19554300 -1.24143400 -1.62026700

O -9.36213400 -1.58945600 -2.19398800

C -10.28436500 -0.57807100 -2.43859600

C -7.31053700 -2.29554000 -1.38819000

C -6.08816500 -2.09107800 -0.81847500

C -5.73641200 -0.79444500 -0.47893200

C -2.93918800 -0.94878200 1.75696500

C -1.83792500 -1.68708400 2.11057000

C 1.98790700 -1.59124400 2.10326600

C 3.04087800 -0.78876200 1.74203900

H 7.48076600 5.43122300 0.55659800

H 6.80026900 5.63233900 -1.08323400

H 6.56280100 6.89775500 0.14382200

H 3.30129500 4.23440800 1.14519700

H 2.74598000 1.78663200 1.06865500

H 7.17645500 3.46664900 -0.53351800

H 8.57097900 1.23090200 -1.45756100

H 10.98899300 -2.07461100 -2.85452100

H 9.36289700 -2.71788900 -3.18436000

H 10.07543800 -2.87547500 -1.55449900

H 7.81296300 -2.98855100 -1.55141100

H 5.58534400 -2.64991700 -0.53440100

H 3.48089800 -1.84245100 -1.43736600

H 1.59149200 -3.32147700 -0.77045200

H -1.38645900 -3.41528300 -0.75442600

H -3.33806200 -2.02785700 -1.41593800

H -2.87152000 1.69033400 1.18291400

H -3.59653100 4.05035300 1.27165300

H -7.01049400 6.52904600 0.23739800

H -7.81958900 4.98859300 0.60497300

H -7.11449700 5.26814200 -1.01205600

H -7.34095000 3.05940200 -0.51072200

H -8.53792500 0.86836800 -1.41517300

H -11.14779800 -1.05105200 -2.90211100

H -10.60037700 -0.08979500 -1.50993300

H -9.88014600 0.17666200 -3.12249000

H -7.63446300 -3.29080700 -1.67251400

H -5.42669000 -2.92901500 -0.62688000

H -3.41698700 -0.29089500 2.47498700

H -1.42426300 -1.62995300 3.11219700

H 1.58168300 -1.56351800 3.10906200

H 3.48706500 -0.10010000 2.45135400

DMOC-DPS_S1_LC-PBE0*_TDA

C 6.75918200 5.92377700 -1.22930200

O 5.44085200 5.45130300 -1.11739800

C 5.25299700 4.15472200 -0.87865900

C 3.91274100 3.75194900 -0.77917600

C 3.58191400 2.44239500 -0.53523100

C 4.62695800 1.53502700 -0.39119700

C 5.97697900 1.92344500 -0.48869400

C 6.30308400 3.23639600 -0.73315600

C 6.75573500 0.70997300 -0.28495300

C 8.08869400 0.41522700 -0.25844000

C 8.47658900 -0.92100800 -0.02491800

O 9.79254200 -1.12116200 -0.01510700

C 10.26122200 -2.42801900 0.21155200

C 7.53217000 -1.92856800 0.17596000

C 6.17960800 -1.63452700 0.15004400

C 5.81134000 -0.31942800 -0.08005600

N 4.54643800 0.19338600 -0.14689800

C 3.35858600 -0.56336300 0.01368300

C 2.89506200 -1.33377400 -1.09145200

C 1.97551200 -2.31701800 -0.89152900

C 1.52289500 -2.61678500 0.41857700

S 0.25430200 -3.73697700 0.64496600

O 0.29306000 -4.21515400 2.02189000

O 0.23159600 -4.65210400 -0.48997100

C -1.24740400 -2.79596800 0.51832200

C -1.82984600 -2.60276600 -0.72397100

C -2.95042600 -1.80148100 -0.83486800

C -3.49620000 -1.21104600 0.29936000

N -4.63741800 -0.40292600 0.18870400

C -4.78390300 0.86671500 0.71549500

C -3.87542300 1.64433100 1.42600600

C -4.27162200 2.90147100 1.81951100

C -5.54662600 3.39803100 1.51578900

O -5.80055900 4.64738600 1.97210700

C -7.06255500 5.17119700 1.69085200

C -6.44473400 2.63519600 0.79516500

C -6.05506900 1.35861900 0.38952300

C -6.70982900 0.33383200 -0.37343000

C -7.97772300 0.24266800 -0.94837300

C -8.31640000 -0.92196900 -1.60904700

O -9.50861300 -1.13738400 -2.21249700

C -10.43990500 -0.10098500 -2.14629000

C -7.40956300 -1.98822700 -1.68598900

C -6.15689900 -1.91081400 -1.12372300

C -5.80567500 -0.73185600 -0.47406500

C -2.91884500 -1.42825700 1.54539800

C -1.78637600 -2.21300500 1.65391000

C 2.02868400 -1.88805500 1.52514600

C 2.94811400 -0.90270500 1.33506600

H 7.31738900 5.76337400 -0.30046200

H 7.28213300 5.44407200 -2.06385000

H 6.67871300 6.99158200 -1.42105200

H 3.15131700 4.51432600 -0.90270800

H 2.55493700 2.10355700 -0.45416400

H 7.33883100 3.54849000 -0.81055300

H 8.86840900 1.15563500 -0.40776300

H 11.34677500 -2.36601600 0.17874700

H 9.91030600 -3.11215000 -0.56799800

H 9.94536800 -2.79396800 1.19399500

H 7.84740900 -2.94969400 0.35397300

H 5.41969400 -2.39345800 0.30230800

H 3.27244200 -1.12582600 -2.08879800

H 1.60248700 -2.90781000 -1.72272600

H -1.40087000 -3.09553800 -1.59130200

H -3.40686600 -1.61606600 -1.80299400

H -2.88006800 1.27770600 1.65752800

H -3.60231100 3.54766700 2.37892900

H -7.08924300 6.16572900 2.13507200

H -7.86066500 4.55839700 2.13031300

H -7.23327800 5.25499700 0.60934300

H -7.43138700 3.00767700 0.53890100

H -8.67225200 1.07242800 -0.86454600

H -11.32479400 -0.44780500 -2.67894500

H -10.71215500 0.13125800 -1.10817500

H -10.06335700 0.81117300 -2.62798600

H -7.73351900 -2.88571400 -2.20358100

H -5.47250400 -2.75189400 -1.17658600

H -3.38337500 -0.99795900 2.42805900

H -1.33308900 -2.41976000 2.61875500

H 1.69611500 -2.15403600 2.52403100

H 3.36614000 -0.36713000 2.18290700

PIC-TRZ_S1_PBE0_TDA

C -12.14215100 -0.02620200 -0.01137800

C -11.44155700 1.07280900 0.47518400

C -10.05502000 1.07773500 0.48213800

C -9.31555500 -0.01424100 -0.00239300

C -7.85135900 -0.00832000 0.00208300

C -7.11435500 1.18789800 -0.12037000

C -5.74005800 1.20222100 -0.11907800

C -4.98926400 0.00234700 0.01015800

C -3.57631700 0.00545000 0.01386900

N -2.89506900 1.21519700 -0.01874600

C -1.61154900 1.10998100 0.03324700

N -0.86048900 2.34214900 0.03508900

C -1.29955400 3.55478400 0.51107800

C -2.51537400 3.90742000 1.10341400

C -2.66189100 5.21487800 1.52000700

C -1.64594000 6.18624000 1.36228000

C -0.44963400 5.84999000 0.78044500

C -0.26121700 4.52502900 0.35010300

C 0.82730300 3.86194700 -0.25722700

C 2.10750200 4.31534400 -0.64475600

C 2.96387100 3.41461300 -1.22442100

C 4.32781000 3.48150600 -1.71484000

C 5.27070700 4.49300700 -1.81472100

C 6.51430800 4.19914400 -2.37198800

C 6.81150200 2.91717900 -2.83266300

C 5.87929900 1.88785300 -2.74639300

C 4.65155400 2.19057300 -2.17717300

N 3.54575800 1.35563500 -1.98766200

C 3.48895700 -0.02358700 -2.33166800

C 4.37850400 -0.91538200 -1.74002900

C 4.29262100 -2.26507100 -2.04924900

C 3.32808100 -2.71607700 -2.94314600

C 2.44906100 -1.81731500 -3.53566300

C 2.52623400 -0.46479700 -3.23471200

C 2.53247800 2.06531100 -1.41726500

C 1.27334400 1.57830000 -1.03251300

C 0.43320400 2.49893700 -0.44408900

N -0.84757000 0.02036300 0.08330400

C -1.59583700 -1.11135700 0.06246800

N -0.86166600 -2.30836300 0.03822300

C -1.32624000 -3.53753200 -0.45859600

C -2.55998500 -3.88041300 -1.00655700

C -2.72777500 -5.18215000 -1.46018300

C -1.70563700 -6.13059600 -1.37328200

C -0.47859700 -5.78385700 -0.83117500

C -0.28473900 -4.48213700 -0.37571500

C 0.85295000 -3.81550300 0.20735500

C 2.12688500 -4.26040400 0.53120200

C 3.01054100 -3.36267900 1.11807500

C 4.38595400 -3.45722200 1.54364600

C 5.32281600 -4.48904300 1.54368100

C 6.58927400 -4.25557700 2.06019700

C 6.92038700 -3.00457600 2.58887900

C 6.00570300 -1.95951600 2.60141500

C 4.74398000 -2.19277600 2.06227200

N 3.64947200 -1.33880300 1.96369200

C 3.60924200 0.00306500 2.39499700

C 2.58524100 0.43976900 3.23560800

C 2.53963100 1.76538000 3.64508100

C 3.52127000 2.66245000 3.23921800

C 4.54944700 2.22559500 2.41219900

C 4.59088600 0.90541700 1.98302100

C 2.58796900 -2.03829900 1.38762200

C 1.32481800 -1.56151200 1.06111800

C 0.46511300 -2.47151100 0.45363200

N -2.89036700 -1.18260400 0.04664300

C -5.73153700 -1.20374000 0.13509400

C -7.10514600 -1.19951100 0.12821000

C -10.04256300 -1.11258200 -0.49136700

C -11.42909800 -1.11925700 -0.49325400

H -13.22781700 -0.03100900 -0.01452300

H -11.98143600 1.93107300 0.86597500

H -9.52976000 1.93023700 0.90193700

H -7.64574400 2.12481000 -0.26366700

H -5.20770400 2.13665000 -0.25566400

H -3.30566000 3.17892200 1.20420600

H -3.59865100 5.51000300 1.98166300

H -1.82249400 7.20054800 1.70326000

H 0.33709100 6.58709400 0.65446500

H 2.39669900 5.34767700 -0.47661600

H 5.04819600 5.49849700 -1.47197000

H 7.26063300 4.98201400 -2.45463800

H 7.78321100 2.71680700 -3.27083800

H 6.10083100 0.89280500 -3.11491500

H 5.10302200 -0.55960100 -1.01570300

H 4.96310200 -2.96581700 -1.56413500

H 3.25173200 -3.77416500 -3.16874300

H 1.69390800 -2.16944600 -4.22990300

H 1.85044000 0.24768100 -3.69601300

H 0.98761700 0.54341800 -1.14787600

H -3.35944500 -3.15634300 -1.05881100

H -3.68565900 -5.46614200 -1.88540500

H -1.87603700 -7.14211500 -1.72871000

H 0.32113900 -6.51590500 -0.76121000

H 2.42136300 -5.28742000 0.33454000

H 5.06046700 -5.46906300 1.15525700

H 7.32558400 -5.05295900 2.06701800

H 7.90981900 -2.84488000 3.00635200

H 6.26602100 -1.00004200 3.03474900

H 1.83481200 -0.26708000 3.57220700

H 1.73728900 2.09409700 4.29808500

H 3.48729100 3.69536700 3.57080200

H 5.32128600 2.91703700 2.08802300

H 5.38479600 0.56848800 1.32456900

H 1.01807400 -0.54407300 1.24849700

H -5.19165700 -2.13403200 0.27127400

H -7.62928400 -2.14104200 0.26703400

H -9.50754300 -1.96116500 -0.90657500

H -11.95898100 -1.98252300 -0.88658900

PIC-TRZ_S1_LC-PBE_TDA

C -12.02210800 -0.05197600 0.02043100

C -11.32548100 -1.08898900 -0.55868100

C -9.94988600 -1.08226700 -0.55877300

C -9.24983200 -0.03797900 0.01636600

C -7.77902400 -0.03053500 0.01411200

C -7.06685700 -1.18649000 0.28109100

C -5.69424700 -1.18244500 0.27556500

C -5.00386300 -0.01664300 0.00978000

C -3.53598700 -0.00885700 0.00669500

N -2.92119800 -1.17388700 0.00491800

C -1.60237700 -1.11017300 0.00982900

N -0.89170900 -2.27585100 0.03425700

C -1.36292000 -3.53735500 -0.34544300

C -2.60037100 -3.93629800 -0.79352300

C -2.76094300 -5.26191500 -1.13622800

C -1.72092600 -6.17322800 -1.03948700

C -0.48660100 -5.77085400 -0.60289600

C -0.30078100 -4.44079000 -0.25719800

C 0.84472000 -3.74193900 0.20335800

C 2.13731300 -4.19325900 0.49222600

C 3.02867900 -3.25874000 0.99760400

C 4.39243800 -3.30137900 1.38756600

C 5.35121600 -4.29930000 1.41886900

C 6.62139500 -3.98998200 1.87925100

C 6.94404900 -2.72150300 2.31457300

C 5.99204900 -1.70277200 2.29464400

C 4.74941200 -2.01101600 1.82160000

N 3.62530400 -1.20424100 1.70257600

C 3.56296200 0.15830500 2.04729300

C 4.48598600 1.04508500 1.53481800

C 4.39115100 2.38024700 1.84850700

C 3.37739500 2.82969000 2.66504400

C 2.46611400 1.93778100 3.18458800

C 2.56008300 0.59934800 2.88493500

C 2.59023100 -1.91765700 1.20321600

C 1.32022600 -1.44603900 0.89963700

C 0.45525200 -2.39723800 0.37676400

N -0.90949700 0.00983700 -0.00256400

C -1.61537400 1.11850300 -0.01039700

N -0.92024300 2.29443200 -0.04168800

C -1.41310500 3.56149600 0.30314700

C -2.65927700 3.94518700 0.75148000

C -2.85063800 5.27332400 1.05567300

C -1.83543500 6.20264100 0.92107900

C -0.59435600 5.81057100 0.48374500

C -0.38229100 4.48373200 0.17844700

C 0.78703400 3.77456100 -0.27616100

C 2.05563400 4.20006200 -0.58523000

C 2.96011700 3.26664100 -1.04111000

C 4.34433200 3.32577300 -1.42656200

C 5.27275000 4.34706100 -1.48391500

C 6.54538800 4.06907200 -1.91524700

C 6.89108100 2.78170100 -2.30193700

C 5.98598300 1.75079800 -2.25558900

C 4.71204800 2.03448800 -1.80225500

N 3.62793400 1.19655600 -1.66202600

C 3.58087700 -0.17220800 -1.98031200

C 2.57420800 -0.64825300 -2.79579200

C 2.49329700 -1.99280100 -3.07244200

C 3.42993800 -2.86482300 -2.56360200

C 4.45170100 -2.38448500 -1.77575700

C 4.52391700 -1.04334700 -1.47614900

C 2.56480000 1.93462600 -1.19351400

C 1.30378400 1.48056800 -0.87372500

C 0.42818800 2.42792800 -0.40292800

N -2.93345600 1.16683400 0.00512800

C -5.70656200 1.14216700 -0.25349400

C -7.07938200 1.13230100 -0.25487900

C -9.95869200 0.99919500 0.59358400

C -11.33427900 0.99202400 0.59751000

H -13.10749100 -0.05743800 0.02200200

H -11.86043100 -1.91172600 -1.02271200

H -9.40451400 -1.89126500 -1.03606300

H -7.60264900 -2.09946900 0.52269400

H -5.13441900 -2.08435100 0.49919200

H -3.41651000 -3.23232100 -0.86756900

H -3.73119700 -5.59641100 -1.48952000

H -1.88847000 -7.20954600 -1.31445600

H 0.33649300 -6.47475900 -0.52968300

H 2.41413200 -5.23061200 0.34471900

H 5.11275400 -5.30894000 1.09960400

H 7.37771800 -4.76840200 1.90697600

H 7.94279400 -2.51201100 2.68103500

H 6.22896300 -0.70787600 2.65604500

H 5.26642900 0.69469700 0.86892300

H 5.10768500 3.07723800 1.42643800

H 3.29536800 3.88619900 2.89790000

H 1.67167400 2.28733300 3.83577200

H 1.86021100 -0.11775900 3.30110500

H 1.04043800 -0.41559300 1.04620100

H -3.45637600 3.22347100 0.85477700

H -3.82581300 5.59431900 1.40813200

H -2.02130400 7.24360300 1.16491200

H 0.21017600 6.53265500 0.38172100

H 2.33278500 5.24484800 -0.47864200

H 4.99704800 5.35774600 -1.19685200

H 7.28570200 4.86070600 -1.96572800

H 7.89856000 2.58431200 -2.65470900

H 6.25823300 0.75128200 -2.57689000

H 1.85447800 0.04801500 -3.21386100

H 1.69040600 -2.36287500 -3.70203400

H 3.36097800 -3.92577000 -2.77933800

H 5.19291700 -3.06164300 -1.36337400

H 5.31494900 -0.67630300 -0.83068000

H 1.03084700 0.44220300 -0.97843300

H -5.15626500 2.04960700 -0.47829600

H -7.62495200 2.03996800 -0.49459300

H -9.42010300 1.81369700 1.06920500

H -11.87613600 1.80936300 1.06306300

PIC-TRZ_S1_LC-PBE0*_TDA

C -12.10264000 -0.00755500 0.17105500

C -11.38888900 1.04754300 0.71610300

C -10.00642500 1.04715400 0.68235500

C -9.30034200 -0.00430500 0.09825100

C -7.83576400 -0.00270800 0.06125500

C -7.11371900 1.18821800 -0.07209100

C -5.74072700 1.19844900 -0.10751500

C -5.00562400 0.00237300 -0.00711300

C -3.58690800 0.00574700 -0.04178600

N -2.91025500 1.19425800 -0.07162200

C -1.62226800 1.12628600 -0.13064300

N -0.88422300 2.30927300 -0.12105200

C -1.36937900 3.56743400 0.24688500

C -2.62921400 3.94993600 0.68745000

C -2.81728500 5.28069100 1.01545800

C -1.79255900 6.21717500 0.90927100

C -0.54042700 5.83016400 0.47469100

C -0.32698100 4.50000200 0.14636600

C 0.83902000 3.78997100 -0.30928300

C 2.12371000 4.20894900 -0.60450300

C 3.03336600 3.26626800 -1.05149700

C 4.42997100 3.32072000 -1.39501200

C 5.36190300 4.34804600 -1.43448300

C 6.65827400 4.06672100 -1.82368500

C 7.02037000 2.77277600 -2.19125300

C 6.10946900 1.73173200 -2.16188200

C 4.81697700 2.01467500 -1.74353900

N 3.72121100 1.17501300 -1.63046500

C 3.71387500 -0.20995500 -1.84326800

C 4.74801400 -1.00529000 -1.35933800

C 4.72649400 -2.37363200 -1.55977700

C 3.66401700 -2.96665200 -2.22244100

C 2.63378800 -2.17669000 -2.70404700

C 2.66243500 -0.80385900 -2.53186900

C 2.63223500 1.92457200 -1.20426000

C 1.35408400 1.47953800 -0.91813800

C 0.46248900 2.43675600 -0.45997100

N -0.87660300 -0.00422800 -0.18767200

C -1.64206300 -1.09097900 -0.12426500

N -0.89784400 -2.31307300 -0.14217000

C -1.35705700 -3.52234300 -0.55270700

C -2.60463400 -3.87317900 -1.08793900

C -2.78445500 -5.18322900 -1.44268600

C -1.77091000 -6.14865400 -1.27720200

C -0.54526300 -5.81390300 -0.75501600

C -0.32105800 -4.49023000 -0.38801200

C 0.79612500 -3.80392700 0.15774400

C 2.06750800 -4.22628300 0.54770200

C 2.93390900 -3.29173400 1.07523000

C 4.29453200 -3.33801000 1.54001500

C 5.23693600 -4.34856600 1.65840700

C 6.48529400 -4.03716300 2.16309700

C 6.79359700 -2.73699600 2.56414900

C 5.87148700 -1.71401200 2.45935300

C 4.62969800 -2.03002400 1.92939800

N 3.53387500 -1.20562500 1.72209500

C 3.48127400 0.18354500 1.95915000

C 2.38418800 0.72457200 2.61431300

C 2.29456800 2.09522200 2.77638300

C 3.30828700 2.91797400 2.31723800

C 4.41919500 2.36933700 1.69576700

C 4.50574900 1.00347300 1.50564700

C 2.51675000 -1.94810800 1.20171000

C 1.25191800 -1.49200200 0.81530400

C 0.41323400 -2.45110100 0.29444200

N -2.91910100 -1.19325400 -0.04287800

C -5.73143400 -1.19580800 0.12751100

C -7.10610900 -1.18995600 0.15925800

C -10.03347100 -1.05711300 -0.44888900

C -11.41586500 -1.06090400 -0.41067100

H -13.18858000 -0.00860400 0.19906900

H -11.91478000 1.87588400 1.18360500

H -9.45554600 1.86323600 1.14323200

H -7.65417300 2.12514400 -0.18791400

H -5.19891500 2.12944100 -0.24509600

H -3.42629900 3.22225900 0.75809300

H -3.79720100 5.59907300 1.36001700

H -1.98107100 7.25491500 1.16846600

H 0.26630400 6.55414200 0.39028400

H 2.40744500 5.25246800 -0.48847100

H 5.07239300 5.36242400 -1.17161300

H 7.39731300 4.86148400 -1.85985400

H 8.03743700 2.57464300 -2.51730700

H 6.39550600 0.73436000 -2.48074800

H 5.57229200 -0.55022400 -0.81891900

H 5.54020600 -2.98071300 -1.17215700

H 3.64282800 -4.04251600 -2.37047900

H 1.80320500 -2.62877900 -3.23863500

H 1.87710800 -0.18074600 -2.94756200

H 1.03988600 0.45443300 -1.04641300

H -3.38002500 -3.12528900 -1.18532700

H -3.73838000 -5.49007200 -1.85982800

H -1.96875600 -7.17544900 -1.56939900

H 0.23106800 -6.56325000 -0.63153200

H 2.36414200 -5.26598300 0.43432800

H 4.99877500 -5.36727100 1.36508500

H 7.23498200 -4.81623400 2.26132400

H 7.77600000 -2.52448900 2.97443600

H 6.10375500 -0.70749700 2.79178400

H 1.61000600 0.06976600 3.00320500

H 1.42517700 2.52127000 3.26707300

H 3.22845800 3.99371300 2.43708100

H 5.20956700 3.00987400 1.31596500

H 5.34980800 0.57844700 0.97195000

H 0.94037800 -0.45855500 0.89148700

H -5.18232900 -2.12545800 0.24162900

H -7.63807900 -2.12806000 0.30244700

H -9.50530100 -1.87125000 -0.93897600

H -11.96364700 -1.88997700 -0.85102800

PPZ-3TPT_S1_PBE0_TDA

C -9.63970100 -0.29022400 -0.10939000

C -8.76048500 0.73893000 0.20219300

C -7.39316300 0.59243000 0.00591200

C -6.86751200 -0.60432400 -0.50826000

C -5.46028400 -0.83623200 -0.78673700

N -5.04814300 -1.69855700 -1.69467900

N -3.72126200 -1.65521000 -1.78385500

C -3.25432200 -0.75824600 -0.90267700

C -1.86214300 -0.54775600 -0.68955600

C -0.96083900 -1.15735100 -1.62146300

C 0.39800400 -1.05074300 -1.49382000

C 0.94912300 -0.32893900 -0.41390500

N 2.38186400 -0.22827000 -0.26896200

C 3.05830500 0.81421900 -0.85857500

C 2.37217100 1.78626900 -1.60267800

C 3.05774600 2.82629000 -2.18876600

C 4.44618900 2.93015900 -2.05054400

C 5.14462900 1.98781600 -1.32507200

C 4.46750000 0.92092400 -0.72221000

N 5.14331600 -0.03926200 0.00839900

C 6.56856100 0.05886800 0.15224700

C 7.09925300 0.76484900 1.22449500

C 8.47903500 0.85699800 1.35963900

C 9.31313100 0.24737600 0.42915800

C 8.77106200 -0.45680500 -0.64017900

C 7.39248300 -0.55412100 -0.78334600

C 4.47316700 -1.09584400 0.59738900

C 5.15605700 -2.07234000 1.33242100

C 4.46315800 -3.11741900 1.90666500

C 3.07477400 -3.20883000 1.75850100

C 2.38337400 -2.25849000 1.04163400

C 3.06378700 -1.18405700 0.44795700

C 0.09972700 0.25989000 0.53124700

C -1.26778900 0.15827600 0.40299900

N -4.34926500 -0.19358000 -0.24271200

C -4.34643900 0.81952700 0.73522200

C -4.90427800 0.58346400 1.99381300

C -4.91833600 1.59129900 2.94693700

C -4.36993500 2.83902100 2.66274100

C -3.81100200 3.07260800 1.40894800

C -3.80232600 2.07370300 0.44641900

C -7.77096900 -1.63362900 -0.83214400

C -9.13076300 -1.47686100 -0.63689600

H -10.70708500 -0.17046400 0.04983500

H -9.14014800 1.67727000 0.59729200

H -6.73988600 1.42568500 0.23291000

H -1.39124500 -1.71839500 -2.44239600

H 1.05110700 -1.52119000 -2.22413700

H 1.29958000 1.68949100 -1.69956800

H 2.51201100 3.56742700 -2.76153300

H 4.98148000 3.75145100 -2.51369000

H 6.21901400 2.06547600 -1.21749300

H 6.43357000 1.23429000 1.94140500

H 8.90166200 1.40639300 2.19415200

H 10.39014700 0.32129900 0.53786000

H 9.42152500 -0.93266800 -1.36633600

H 6.95247800 -1.09977900 -1.61168200

H 6.23032100 -2.00007900 1.44478400

H 5.00269200 -3.86822000 2.47302300

H 2.53324700 -4.03274300 2.20908300

H 1.31074400 -2.31480300 0.91694300

H 0.51954100 0.79248500 1.38092200

H -1.88411500 0.60306600 1.17204800

H -5.32852500 -0.39151500 2.20832400

H -5.35731800 1.39875800 3.92127600

H -4.38141600 3.62479800 3.41156900

H -3.38875800 4.04523800 1.17411000

H -3.37704200 2.24841700 -0.53628400

H -7.37110900 -2.55138300 -1.24828200

H -9.80376600 -2.29038800 -0.89336300

PPZ-3TPT_S1_LC-PBE_TDA

C -9.43309600 0.04862200 0.04882900

C -8.49284500 1.05142700 -0.01714200

C -7.16881800 0.74807200 -0.23906900

C -6.77714800 -0.56819100 -0.39257700

C -5.39852900 -0.96392200 -0.66484000

N -5.07282200 -1.99838500 -1.36876100

N -3.73552200 -2.07196900 -1.41442200

C -3.25206600 -1.08528900 -0.73456300

C -1.82161600 -0.84676800 -0.56500200

C -0.98400100 -1.27926900 -1.57744100

C 0.37576800 -1.11647500 -1.47057400

C 0.89612200 -0.52181000 -0.34573000

N 2.29877900 -0.34471600 -0.23258700

C 2.84850800 0.81234100 -0.69109000

C 2.08779800 1.83232600 -1.27962300

C 2.66807800 2.97248600 -1.72457000

C 4.06488700 3.15451900 -1.60151000

C 4.83088800 2.18885500 -1.03999400

C 4.26522000 0.99539700 -0.56943600

N 5.00472200 0.00371100 -0.00347700

C 6.40787600 0.18306200 0.11293300

C 6.92024300 0.76498600 1.24725200

C 8.28202400 0.93741000 1.35717500

C 9.11285900 0.52841100 0.33714400

C 8.58690500 -0.05380100 -0.79536300

C 7.22600500 -0.22892700 -0.91117600

C 4.45464700 -1.15312000 0.45351500

C 5.21672100 -2.17554500 1.03721100

C 4.63596300 -3.31495900 1.48241100

C 3.23868200 -3.49473800 1.36461100

C 2.47182400 -2.52850600 0.80595200

C 3.03829200 -1.33556500 0.33432500

C 0.08033100 -0.10081700 0.67502500

C -1.28155100 -0.26552900 0.56702400

N -4.27102800 -0.33808300 -0.22985500

C -4.17764800 0.82628400 0.56692600

C -4.64773900 0.80241100 1.85987300

C -4.55408700 1.93746400 2.63168200

C -3.98548900 3.08065200 2.11423000

C -3.51466400 3.09312900 0.81958500

C -3.61457300 1.96517200 0.03828600

C -7.72738600 -1.57135600 -0.34016100

C -9.04782600 -1.26370600 -0.11879900

H -10.47627100 0.29191100 0.22470300

H -8.79297800 2.08801400 0.09826200

H -6.44252400 1.55079300 -0.30741100

H -1.42399000 -1.75754100 -2.44566900

H 1.04607100 -1.44718200 -2.25708000

H 1.01632100 1.69501900 -1.37751900

H 2.05779300 3.74558400 -2.17842500

H 4.52733000 4.06811700 -1.95851000

H 5.90227200 2.33133600 -0.94890600

H 6.24268200 1.07738600 2.03527500

H 8.69793900 1.39555300 2.24856000

H 10.18589900 0.66517900 0.42612300

H 9.24267000 -0.37562000 -1.59775000

H 6.78482400 -0.68469100 -1.79169400

H 6.28915700 -2.04174500 1.12865700

H 5.24740400 -4.09104000 1.92943300

H 2.77657400 -4.40904200 1.72013700

H 1.40046800 -2.67197400 0.71569400

H 0.52252000 0.35079900 1.55687300

H -1.91979900 0.05181200 1.38398500

H -5.09034700 -0.10838000 2.25018500

H -4.92694100 1.92688900 3.65054400

H -3.90819100 3.97272900 2.72727400

H -3.06573600 3.99244300 0.41107100

H -3.25306600 1.95636500 -0.98504300

H -7.40636500 -2.59676900 -0.48943600

H -9.78599400 -2.05824100 -0.07758600

PPZ-3TPT_S1_LC-PBE0*_TDA

C -9.55515000 -0.29640900 0.04371200

C -8.67166400 0.74532800 0.27424600

C -7.31707500 0.59162900 0.02207900

C -6.82214500 -0.62148500 -0.46052500

C -5.42547300 -0.86126200 -0.78926100

N -5.04024500 -1.72555500 -1.68838100

N -3.70256600 -1.69551800 -1.78397900

C -3.23511100 -0.79493300 -0.92336900

C -1.84974000 -0.52986100 -0.73369000

C -0.95086000 -1.08434300 -1.69695000

C 0.39833700 -0.92212800 -1.59910600

C 0.93979700 -0.18396000 -0.51772100

N 2.35486000 -0.13567800 -0.33335700

C 3.08102100 0.90351200 -0.85665600

C 2.44426200 1.92131600 -1.57430800

C 3.17625400 2.96010900 -2.09606000

C 4.55913000 3.01026600 -1.91437900

C 5.20844700 2.01963800 -1.21223400

C 4.48187400 0.95561500 -0.67652900

N 5.09994900 -0.05247500 0.02814200

C 6.51456600 -0.00913600 0.21437500

C 7.03348000 0.62864300 1.32903700

C 8.40752600 0.66804600 1.50592800

C 9.24531500 0.07380700 0.57375700

C 8.71396700 -0.56211300 -0.53858700

C 7.34112200 -0.60619200 -0.72319700

C 4.38371100 -1.10571900 0.55024600

C 5.01053900 -2.13133900 1.25853700

C 4.26322000 -3.17148700 1.76410600

C 2.88099500 -3.20994800 1.57400700

C 2.24547300 -2.20840100 0.88095000

C 2.98333500 -1.14006400 0.35862300

C 0.08332200 0.31482100 0.48589600

C -1.27095400 0.15257900 0.37832200

N -4.31359200 -0.22813400 -0.26479700

C -4.29783300 0.80693700 0.68652100

C -4.81322600 0.58963300 1.95794500

C -4.81893800 1.62070400 2.88273700

C -4.30075300 2.86327200 2.54755400

C -3.77925300 3.07337300 1.27829400

C -3.78105800 2.05092100 0.34497900

C -7.72358500 -1.66242000 -0.70228800

C -9.07280300 -1.49989500 -0.45508200

H -10.61506800 -0.17072600 0.24427200

H -9.03828200 1.69745200 0.64731400

H -6.64921200 1.43082600 0.18492800

H -1.38356700 -1.64524300 -2.51879700

H 1.05912600 -1.35087900 -2.34830100

H 1.37025400 1.84875300 -1.69608100

H 2.67261800 3.74276500 -2.65234500

H 5.13323300 3.83253000 -2.32775100

H 6.28265700 2.05489300 -1.07016900

H 6.35889800 1.08666900 2.04591000

H 8.82441800 1.16496200 2.37576300

H 10.32079900 0.10635600 0.71535700

H 9.37034200 -1.02683300 -1.26696500

H 6.90333000 -1.09869100 -1.58608200

H 6.08450400 -2.09924000 1.40322100

H 4.75931800 -3.96498600 2.31258300

H 2.30002800 -4.03387200 1.97282200

H 1.17471900 -2.21337800 0.71691400

H 0.50190800 0.81975200 1.35294400

H -1.89611200 0.53191600 1.17850400

H -5.21549600 -0.38829300 2.20302000

H -5.22872700 1.45019900 3.87362500

H -4.30368200 3.66884800 3.27546800

H -3.37348700 4.04413800 1.01003100

H -3.37493800 2.19334900 -0.65166000

H -7.33069000 -2.59226500 -1.10054200

H -9.75614600 -2.32182400 -0.64883700

PPZ-DPO_S1_PBE0_TDA

C -9.68275300 -0.00021500 1.76709700

C -8.44552500 -0.00030800 2.40231400

C -7.27180900 -0.00023100 1.66294000

C -7.31737600 -0.00005600 0.26060700

C -6.11535900 0.00004100 -0.52865100

N -5.99941500 0.00027300 -1.82786500

N -4.68631300 0.00017800 -2.12999100

C -4.02416200 0.00015500 -0.97953300

C -2.63251500 0.00012900 -0.76716300

C -2.05783400 0.00007000 0.53910400

C -0.69446300 0.00003900 0.71304000

C 0.15842500 0.00006400 -0.40190000

N 1.59095900 0.00003400 -0.21677600

C 2.26877700 1.19407900 -0.13228200

C 1.58655800 2.41707700 -0.22360100

C 2.27484800 3.60624700 -0.13712300

C 3.66234100 3.61372600 0.04329100

C 4.35702300 2.42597800 0.13559800

C 3.67706900 1.20510300 0.04932200

N 4.34953600 -0.00002400 0.13727600

C 5.77391800 -0.00005400 0.31958000

C 6.29711000 -0.00013200 1.60647000

C 7.67593000 -0.00016000 1.77788200

C 8.51638300 -0.00011000 0.67027500

C 7.98169500 -0.00003200 -0.61319900

C 6.60413300 -0.00000300 -0.79426200

C 3.67703300 -1.20512200 0.04920500

C 4.35695200 -2.42602600 0.13535900

C 3.66223400 -3.61374400 0.04293800

C 2.27474000 -3.60620600 -0.13747000

C 1.58648600 -2.41700800 -0.22383100

C 2.26874100 -1.19403900 -0.13239600

C -0.38145600 0.00012100 -1.70307300

C -1.74018100 0.00015200 -1.88273300

O -4.89318200 0.00004400 0.08567500

C -8.57042800 0.00004500 -0.37511800

C -9.73354000 -0.00003800 0.37250600

H -10.59898900 -0.00027700 2.34947300

H -8.39320100 -0.00044500 3.48759300

H -6.30932200 -0.00030500 2.16307800

H -2.71065000 0.00004900 1.40464800

H -0.27540100 -0.00000600 1.71587000

H 0.51468300 2.39506300 -0.36341800

H 1.73141900 4.54136800 -0.21050500

H 4.19944900 4.55311100 0.11106200

H 5.43057700 2.42876800 0.27468600

H 5.62663300 -0.00017000 2.45966000

H 8.09280600 -0.00022100 2.77940900

H 9.59262000 -0.00013200 0.80789900

H 8.63715900 0.00000800 -1.47761000

H 6.17000800 0.00005800 -1.78875600

H 5.43050500 -2.42886100 0.27444300

H 4.19931400 -4.55315100 0.11061500

H 1.73128300 -4.54130400 -0.21094100

H 0.51461100 -2.39494800 -0.36364200

H 0.28090600 0.00014100 -2.56462800

H -2.16480900 0.00019500 -2.88017600

H -8.60114900 0.00018600 -1.45931800

H -10.69434100 0.00003900 -0.13452000

PPZ-DPO_S1_LC-PBE_TDA

C 9.51962500 -0.00004900 1.78579700

C 8.29125900 -0.00000900 2.40742600

C 7.13877400 0.00002000 1.65643000

C 7.22020700 0.00000900 0.27926000

C 6.02424300 0.00003200 -0.53917000

N 5.93493400 -0.00004500 -1.81243800

N 4.60994200 0.00006100 -2.11219700

C 3.98254100 0.00001900 -1.00187400

C 2.54973400 0.00002900 -0.78070800

C 2.03001100 0.00001400 0.49749700

C 0.66644900 0.00001900 0.67968500

C -0.15885900 0.00003200 -0.41809500

N -1.56342200 0.00002000 -0.22873100

C -2.20911600 -1.19449200 -0.14083900

C -1.54361700 -2.42518500 -0.23237700

C -2.21927700 -3.59529600 -0.14102600

C -3.62016900 -3.59481100 0.04959100

C -4.29522600 -2.42449900 0.14136200

C -3.62921100 -1.19364200 0.05131800

N -4.27556500 -0.00000600 0.13822700

C -5.68286200 -0.00002000 0.32477300

C -6.19268700 -0.00007800 1.60066700

C -7.55823100 -0.00009500 1.77799300

C -8.39517600 -0.00005200 0.68367500

C -7.87161400 0.00000700 -0.59070700

C -6.50700500 0.00002200 -0.77462000

C -3.62922600 1.19364200 0.05137300

C -4.29526100 2.42448700 0.14145600

C -3.62022000 3.59481200 0.04973100

C -2.21932700 3.59532400 -0.14087900

C -1.54365100 2.42522700 -0.23227500

C -2.20913200 1.19452000 -0.14078800

C 0.35003700 0.00005000 -1.69627000

C 1.71094100 0.00005300 -1.87895300

O 4.81837500 -0.00000200 0.04273400

C 8.45185900 -0.00003400 -0.34651200

C 9.59825700 -0.00006000 0.40908100

H 10.42764000 -0.00007200 2.38049600

H 8.22899300 -0.00000200 3.49074600

H 6.16758000 0.00005000 2.14005500

H 2.69652500 -0.00000200 1.35308300

H 0.22956400 0.00000500 1.67266000

H -0.46904100 -2.43140400 -0.38020100

H -1.68151300 -4.53396200 -0.21585600

H -4.15852600 -4.53333000 0.12200400

H -5.37002300 -2.42828100 0.28678000

H -5.51044500 -0.00011200 2.44459800

H -7.97225600 -0.00014200 2.78098700

H -9.47115700 -0.00006400 0.82603100

H -8.53218800 0.00004200 -1.45154100

H -6.06774000 0.00006800 -1.76695600

H -5.37005800 2.42824700 0.28687100

H -4.15859200 4.53332000 0.12217300

H -1.68157800 4.53400200 -0.21567200

H -0.46907500 2.43146700 -0.38009900

H -0.33224500 0.00006100 -2.53974400

H 2.14615600 0.00006800 -2.87268200

H 8.48884800 -0.00004500 -1.43099200

H 10.56648900 -0.00009200 -0.08077300

PPZ-DPO_S1_LC-PBE0*_TDA

C 9.61591100 -0.00001900 1.78101900

C 8.37923300 -0.00001800 2.40802700

C 7.21375900 -0.00001000 1.65914100

C 7.27731700 -0.00000300 0.26620700

C 6.07974600 0.00000400 -0.53568300

N 5.97938900 0.00000800 -1.82388700

N 4.66012400 0.00001600 -2.13527300

C 4.00238900 0.00001000 -0.99080700

C 2.61491500 0.00001200 -0.77277200

C 2.04934200 0.00000500 0.53456900

C 0.69380700 0.00000800 0.71361200

C -0.16113800 0.00002000 -0.40586000

N -1.57891000 0.00001200 -0.21986300

C -2.25460800 -1.19057200 -0.13408300

C -1.56767100 -2.40603300 -0.22766300

C -2.25268400 -3.59387600 -0.14020700

C -3.63672900 -3.60072100 0.04280400

C -4.33471600 -2.41759300 0.13712900

C -3.65675700 -1.20078800 0.04978700

N -4.32451300 -0.00000400 0.13834000

C -5.74091900 -0.00001200 0.32223700

C -6.25880600 -0.00006000 1.60717800

C -7.63411700 -0.00006800 1.78060400

C -8.47383400 -0.00002700 0.67628700

C -7.94326500 0.00002200 -0.60559100

C -6.56919900 0.00002900 -0.78832200

C -3.65676800 1.20078900 0.04980400

C -4.33473900 2.41758600 0.13717200

C -3.63676300 3.60072200 0.04286200

C -2.25271900 3.59389300 -0.14015900

C -1.56769500 2.40605800 -0.22763600

C -2.25462000 1.19058900 -0.13406900

C 0.37507500 0.00002400 -1.71193300

C 1.72778400 0.00002100 -1.88973900

O 4.86417700 0.00000200 0.07005100

C 8.52434800 -0.00000500 -0.36279200

C 9.68099800 -0.00001300 0.39194300

H 10.52765900 -0.00002600 2.37062100

H 8.32010200 -0.00002200 3.49260800

H 6.24366900 -0.00000800 2.14565000

H 2.70933500 -0.00000200 1.39583100

H 0.27267500 0.00000300 1.71563500

H -0.49472500 -2.37098300 -0.37011500

H -1.70983600 -4.52928200 -0.21496900

H -4.17215000 -4.54134800 0.11137500

H -5.40938700 -2.41846800 0.27862200

H -5.58328700 -0.00009100 2.45698100

H -8.05026500 -0.00010600 2.78253700

H -9.54996200 -0.00003300 0.81569800

H -8.60099100 0.00005400 -1.46839900

H -6.13251300 0.00006700 -1.78220800

H -5.40940800 2.41844800 0.27867600

H -4.17219200 4.54134200 0.11145500

H -1.70988100 4.52930500 -0.21491000

H -0.49475000 2.37102000 -0.37009300

H -0.29185300 0.00003100 -2.57014100

H 2.15928400 0.00002600 -2.88522100

H 8.55492900 0.00000000 -1.44772600

H 10.64626100 -0.00001400 -0.10592300

PXZ-OXD_S1_PBE0_TDA

C 8.38152700 0.00004300 -1.53955300

C 7.17028100 0.00000000 -2.22244900

C 5.96902500 -0.00000600 -1.52821200

C 5.96199300 0.00002700 -0.12622700

C 4.72922200 -0.00001700 0.61784100

N 4.56485900 -0.00010300 1.90996600

N 3.23912800 0.00000000 2.16229400

C 2.62347900 -0.00014100 0.98715400

C 1.24159200 -0.00019400 0.72030300

C 0.71767000 -0.00028400 -0.60801300

C -0.63647600 -0.00026800 -0.83562400

C -1.53051600 -0.00014200 0.24913500

N -2.95403000 -0.00003000 0.00906000

C -3.64051200 -1.18491200 -0.10384900

C -2.99911900 -2.42993000 0.00882300

C -3.72994600 -3.58965500 -0.11170600

C -5.11223500 -3.55139400 -0.34706800

C -5.76441300 -2.33945800 -0.46133200

C -5.03328200 -1.16670900 -0.33993600

O -5.70649800 0.00019300 -0.45688200

C -5.03309700 1.16698700 -0.33991900

C -5.76404300 2.33985200 -0.46130200

C -5.11167400 3.55168400 -0.34701800

C -3.72938000 3.58972300 -0.11165100

C -2.99873500 2.42988100 0.00885700

C -3.64032500 1.18496500 -0.10383000

C -1.04294400 -0.00005200 1.57252000

C 0.30715100 -0.00009300 1.80213500

O 3.53280100 -0.00013700 -0.04305000

C 7.18817600 0.00006300 0.55783700

C 8.37958700 0.00007700 -0.14453300

H 9.31944200 0.00004500 -2.08632600

H 7.15997100 -0.00003000 -3.30881400

H 5.02632900 -0.00004300 -2.06469900

H 1.40335400 -0.00035200 -1.44773400

H -1.01642600 -0.00032800 -1.85381600

H -1.93301400 -2.44292800 0.19088100

H -3.22607300 -4.54514300 -0.02237400

H -5.67336300 -4.47419000 -0.43958400

H -6.83076300 -2.27140000 -0.64251200

H -6.83040300 2.27196500 -0.64248400

H -5.67265500 4.47456900 -0.43952500

H -3.22535600 4.54513000 -0.02230800

H -1.93262800 2.44271000 0.19091000

H -1.73741000 0.00004800 2.40831400

H 0.69375900 -0.00002800 2.81489300

H 7.17682700 0.00007800 1.64239700

H 9.32026200 0.00010600 0.39869000

PXZ-OXD_S1_LC-PBE_TDA

C 8.24880500 0.00006200 -1.55084400

C 7.04710800 0.00006500 -2.22264100

C 5.86471900 0.00003500 -1.51976500

C 5.88956100 0.00000000 -0.14038900

C 4.66120400 -0.00003300 0.62832000

N 4.51975500 -0.00004000 1.89707800

N 3.18388000 -0.00012100 2.14235800

C 2.60275000 -0.00002600 1.00743500

C 1.17967400 -0.00001600 0.72966400

C 0.71110500 0.00007000 -0.56805900

C -0.64432900 0.00007700 -0.80428200

C -1.50987000 -0.00000100 0.26181700

N -2.90603100 0.00000200 0.01734600

C -3.56046900 -1.18521400 -0.09840700

C -2.94053900 -2.43941200 0.01253800

C -3.66034600 -3.57921200 -0.11387100

C -5.05559100 -3.53346400 -0.36071900

C -5.68419100 -2.33745600 -0.47236000

C -4.96703800 -1.15369400 -0.34639300

O -5.61074700 0.00000800 -0.46020800

C -4.96704900 1.15370700 -0.34630300

C -5.68421500 2.33747200 -0.47216600

C -5.05563000 3.53347700 -0.36040900

C -3.66038700 3.57921800 -0.11354200

C -2.94057000 2.43941500 0.01277200

C -3.56048200 1.18522100 -0.09830300

C -1.05391800 -0.00009200 1.55964200

C 0.29904500 -0.00009800 1.79465900

O 3.47984800 0.00000800 -0.00233100

C 7.09433500 -0.00000100 0.53570800

C 8.27083300 0.00002900 -0.17208600

H 9.18050800 0.00008500 -2.10767000

H 7.02960200 0.00009100 -3.30758800

H 4.91439400 0.00003600 -2.04321400

H 1.41080100 0.00013100 -1.39664900

H -1.04142800 0.00014700 -1.81385800

H -1.87337100 -2.48484000 0.20299300

H -3.16214200 -4.53809300 -0.02378100

H -5.61832300 -4.45487200 -0.45903400

H -6.75065500 -2.26556900 -0.66026400

H -6.75067600 2.26558900 -0.66008400

H -5.61837200 4.45488700 -0.45864200

H -3.16219800 4.53809700 -0.02334900

H -1.87340500 2.48483700 0.20324800

H -1.76822900 -0.00015500 2.37621500

H 0.69537900 -0.00016600 2.80449400

H 7.08688200 -0.00002600 1.62079000

H 9.21809300 0.00002700 0.35713800

PXZ-OXD_S1_LC-PBE0*_TDA

C 8.32526200 0.00008500 -1.54886000

C 7.11486100 0.00013800 -2.22518300

C 5.92044500 0.00009900 -1.52331300

C 5.92904900 0.00000500 -0.12929900

C 4.70014000 -0.00004300 0.62512700

N 4.54905300 -0.00014400 1.90785400

N 3.21711900 -0.00015800 2.16605600

C 2.60734300 -0.00007800 0.99689100

C 1.22883500 -0.00006400 0.72393400

C 0.71533500 0.00004200 -0.60455000

C -0.63113100 0.00004300 -0.83820800

C -1.52781700 -0.00004800 0.25038200

N -2.93587900 -0.00000900 0.00967700

C -3.62155200 -1.18091100 -0.10496100

C -2.97234000 -2.41655200 0.01055100

C -3.69548900 -3.57801600 -0.11055800

C -5.07301200 -3.54194900 -0.34848800

C -5.73098600 -2.33553600 -0.46518700

C -5.00845800 -1.16227100 -0.34339600

O -5.68134300 0.00006700 -0.46161600

C -5.00840500 1.16236900 -0.34333500

C -5.73087900 2.33567100 -0.46507900

C -5.07285800 3.54204900 -0.34829800

C -3.69533800 3.57804100 -0.11033600

C -2.97223900 2.41654100 0.01071200

C -3.62149900 1.18093000 -0.10487400

C -1.04470300 -0.00013400 1.57861300

C 0.29977300 -0.00014900 1.80646400

O 3.50988300 0.00000500 -0.02831500

C 7.14930200 -0.00004700 0.54950800

C 8.33532100 -0.00000800 -0.15846500

H 9.25978300 0.00011400 -2.10159200

H 7.09932100 0.00020900 -3.31117700

H 4.97064600 0.00014200 -2.04830600

H 1.40858300 0.00009500 -1.43924300

H -1.01247900 0.00016200 -1.85586800

H -1.90500000 -2.41318600 0.19563000

H -3.18912500 -4.53208800 -0.01951900

H -5.63139200 -4.46667500 -0.44191600

H -6.79758800 -2.27317500 -0.64887000

H -6.79748100 2.27336300 -0.64878400

H -5.63119600 4.46680400 -0.44169000

H -3.18893200 4.53208500 -0.01924300

H -1.90490100 2.41312800 0.19580200

H -1.74386600 -0.00022900 2.41060200

H 0.69297100 -0.00022100 2.81767100

H 7.13655600 -0.00012200 1.63477000

H 9.27995900 -0.00005000 0.37732900

PXZ-TAZ_S1_PBE0_TDA

C 8.35425300 -0.33881700 -0.26197700

C 7.82415000 -1.46953400 -0.88187900

C 6.45931100 -1.59435500 -1.06779700

C 5.57505100 -0.58733800 -0.64146000

C 4.15924600 -0.77898200 -0.91523100

N 3.72021100 -1.55094000 -1.88677800

N 2.38949200 -1.49383900 -1.93709700

C 1.95259100 -0.67814900 -0.96718000

C 0.56899200 -0.47032200 -0.70439400

C 0.00838400 0.14786700 0.45845500

C -1.35222600 0.25564400 0.62827500

C -2.23052000 -0.24143400 -0.34760000

N -3.65675100 -0.14324200 -0.15796200

C -4.35123000 0.94331100 -0.63291300

C -3.71198900 1.99187700 -1.31519200

C -4.44872300 3.06014400 -1.77314400

C -5.83491900 3.12177000 -1.56863000

C -6.48501900 2.10249700 -0.90082900

C -5.74788900 1.02210000 -0.43820300

O -6.41950600 0.04299400 0.20942300

C -5.73930600 -1.03197100 0.66875600

C -6.46767300 -2.01703400 1.31956500

C -5.80833500 -3.13106100 1.80099300

C -4.42187800 -3.25938700 1.63137400

C -3.69355700 -2.28507200 0.98810200

C -4.34228500 -1.14233800 0.49040700

C -1.71457200 -0.87932900 -1.49830700

C -0.36149700 -0.99030000 -1.66405600

N 3.06779600 -0.18342900 -0.28742400

C 3.09816500 0.73693800 0.78010600

C 2.59711000 2.02838100 0.60623300

C 2.63858500 2.93546700 1.65541700

C 3.18849100 2.56946500 2.88081900

C 3.69399800 1.28398200 3.05035100

C 3.64622900 0.36771800 2.00922000

C 6.12119200 0.55411700 -0.03411800

C 7.49299000 0.66884000 0.15252400

H 9.42531000 -0.24451800 -0.11056200

H 8.48366200 -2.26440700 -1.21881600

H 6.04179900 -2.46810900 -1.55518400

H 0.64804200 0.51930300 1.24735000

H -1.74517800 0.71867100 1.52984100

H -2.64261600 1.92633000 -1.46439200

H -3.94691600 3.86352400 -2.30006500

H -6.40118500 3.97022300 -1.93538300

H -7.55445800 2.11603000 -0.72557200

H -7.53754500 -1.88499400 1.43147400

H -6.36753700 -3.90755000 2.31030000

H -3.91278000 -4.13798800 2.01043900

H -2.62423000 -2.36862300 0.84754600

H -2.38952400 -1.27987100 -2.25005100

H 0.04331700 -1.48566900 -2.53849400

H 2.17846600 2.30456500 -0.35585700

H 2.24936100 3.93899200 1.51159800

H 3.22613100 3.28327100 3.69777600

H 4.12601600 0.98997400 4.00210100

H 4.03709800 -0.63663300 2.13220900

H 5.48074900 1.37077800 0.27539000

H 7.89013900 1.56474600 0.62168400

PXZ-TAZ_S1_LC-PBE_TDA

C 8.19196900 -0.15334000 -0.05662300

C 7.75936900 -1.44011500 -0.29276800

C 6.42586500 -1.69013800 -0.50819700

C 5.51002600 -0.65437500 -0.48584400

C 4.11463400 -0.98777300 -0.75582600

N 3.74251300 -1.96998500 -1.51021000

N 2.40335400 -1.99679800 -1.53539600

C 1.96478100 -1.03451900 -0.79307200

C 0.54516700 -0.76364800 -0.58474500

C 0.04215000 -0.21685800 0.58100600

C -1.31275700 -0.02189000 0.72308700

C -2.15616800 -0.37973200 -0.29904800

N -3.55148700 -0.17390800 -0.14996500

C -4.09626100 1.00531500 -0.54936200

C -3.36239600 2.05483200 -1.12261300

C -3.97506900 3.20326100 -1.49692900

C -5.37121700 3.37278500 -1.31854900

C -6.10889000 2.37672400 -0.76810800

C -5.50321100 1.18904500 -0.37686100

O -6.25210200 0.23386600 0.15790600

C -5.71542500 -0.91546600 0.54412900

C -6.53968600 -1.88742700 1.09844200

C -6.02212400 -3.07368800 1.50275800

C -4.63475700 -3.32608400 1.35993700

C -3.81149800 -2.39450000 0.82332700

C -4.31485300 -1.15605900 0.39580600

C -1.67486900 -0.93906900 -1.45876400

C -0.32187200 -1.13110300 -1.59827300

N 3.01625300 -0.35056100 -0.26642200

C 2.97589800 0.76938900 0.59611800

C 2.45307000 1.95747900 0.13928200

C 2.40466000 3.04195300 0.98448800

C 2.88627200 2.93710200 2.27098600

C 3.41454100 1.74516600 2.71616000

C 3.45689500 0.65340500 1.87999600

C 5.94886000 0.63693900 -0.26346300

C 7.28584700 0.88246800 -0.04793800

H 9.24550800 0.04436200 0.11446000

H 8.47039800 -2.25984100 -0.31048300

H 6.06772800 -2.69391300 -0.71061400

H 0.70329200 0.04923800 1.39788400

H -1.72592300 0.40307600 1.63185400

H -2.29382400 1.93178300 -1.26430200

H -3.38941000 4.00095900 -1.94043500

H -5.84836700 4.29732300 -1.62351100

H -7.17953500 2.47211500 -0.61813100

H -7.59701200 -1.66126100 1.19182800

H -6.66863000 -3.82974700 1.93389300

H -4.22536800 -4.27711600 1.68209800

H -2.75109200 -2.59725400 0.71587400

H -2.36758700 -1.21948600 -2.24539200

H 0.08970600 -1.58343000 -2.49384800

H 2.08333800 2.02145300 -0.87916900

H 1.98856500 3.98021500 0.63274300

H 2.84982300 3.79499800 2.93450400

H 3.79629100 1.66213900 3.72835900

H 3.86772700 -0.29441500 2.21262200

H 5.24978600 1.46622700 -0.27249500

H 7.62325900 1.89993700 0.12172000

PXZ-TAZ_S1_LC-PBE0*_TDA

C 8.27106600 -0.34221900 -0.11054900

C 7.76170600 -1.52291600 -0.63536200

C 6.40628300 -1.65578600 -0.86678300

C 5.52789800 -0.60699800 -0.58190900

C 4.12131900 -0.81394300 -0.89452300

N 3.70439400 -1.64905200 -1.80603700

N 2.36332700 -1.59739200 -1.86759200

C 1.93236800 -0.71290700 -0.97632400

C 0.55497800 -0.42111100 -0.75219100

C 0.01472100 0.22147400 0.39970700

C -1.33098200 0.41255300 0.54338700

C -2.22078300 0.00024700 -0.47914600

N -3.61809600 -0.07059800 -0.18966000

C -4.46984300 0.92920900 -0.58043600

C -4.00497900 2.05086900 -1.27662400

C -4.88758400 3.03344400 -1.65326900

C -6.24698900 2.92624900 -1.34773300

C -6.72491700 1.82904500 -0.66254100

C -5.84075400 0.83585400 -0.28201000

O -6.34218400 -0.22285900 0.38487200

C -5.50777700 -1.21283500 0.76147700

C -6.05449400 -2.28933600 1.43514300

C -5.22756000 -3.31945300 1.83173100

C -3.85787300 -3.27609700 1.55689800

C -3.30792900 -2.20905600 0.88951500

C -4.13100600 -1.15214200 0.47893000

C -1.71540600 -0.72798700 -1.59028700

C -0.37345100 -0.91715800 -1.71841600

N 3.03223800 -0.18017000 -0.32911600

C 3.05011000 0.82894500 0.65194900

C 2.57413400 2.09679200 0.34384200

C 2.60205700 3.09181400 1.30613500

C 3.11296600 2.82826300 2.56950400

C 3.59052800 1.56121100 2.87062100

C 3.55474800 0.55765400 1.91640800

C 6.04911200 0.58335800 -0.07287700

C 7.40958400 0.70743300 0.16301400

H 9.33585000 -0.23983000 0.07702300

H 8.42826300 -2.34996400 -0.86252500

H 5.99153300 -2.56719300 -1.28505400

H 0.66572600 0.55252300 1.20133300

H -1.71994700 0.88229900 1.44316300

H -2.94438200 2.09586300 -1.49493800

H -4.52313400 3.89969600 -2.19393400

H -6.93462900 3.70840900 -1.65033400

H -7.77352100 1.71548200 -0.41113100

H -7.12080500 -2.29395400 1.63183500

H -5.64777600 -4.16861400 2.35949500

H -3.21841700 -4.09311600 1.87116200

H -2.25014900 -2.15140600 0.66149100

H -2.39919900 -1.11579500 -2.34112400

H 0.03102300 -1.45357500 -2.57062900

H 2.17558200 2.28010800 -0.64917400

H 2.22793500 4.08228600 1.06560800

H 3.13946600 3.61221800 3.32019200

H 3.99240100 1.35021400 3.85688800

H 3.92525000 -0.43932800 2.13330400

H 5.39762600 1.42817800 0.12424600

H 7.79821900 1.64204000 0.55726800

PXZ-TRZ_S1_PBE0_TDA

C -5.92217200 -4.82167200 -0.00024700

C -6.60211800 -3.60937100 -0.00030500

C -5.89770100 -2.41236200 -0.00027400

C -4.50179700 -2.41179100 -0.00019200

C -3.75187900 -1.13079300 -0.00017100

N -4.48448600 0.00000300 -0.00011000

C -3.75187500 1.13079800 -0.00007500

C -4.50178700 2.41179900 0.00011100

C -5.89769200 2.41237600 0.00020100

C -6.60210200 3.60939000 0.00036000

C -5.92215000 4.82168700 0.00043500

C -4.53003000 4.82892500 0.00034800

C -3.82603100 3.63396100 0.00019000

N -2.43711000 1.19558200 -0.00000100

C -1.78552700 0.00000000 -0.00001000

C -0.35478100 -0.00000100 -0.00001000

C 0.38446300 1.21439900 0.00006900

C 1.75982900 1.21689600 0.00005800

C 2.45580800 -0.00000200 -0.00001600

N 3.90106500 0.00000200 -0.00000100

C 4.59437600 0.00016700 -1.18592200

C 3.94279200 0.00031200 -2.43064500

C 4.68383700 0.00047600 -3.59017600

C 6.08601300 0.00049600 -3.55154500

C 6.74809300 0.00035600 -2.33966600

C 6.00689200 0.00019500 -1.16703200

O 6.68971000 0.00007900 0.00002700

C 6.00686800 -0.00014400 1.16707300

C 6.74804400 -0.00032200 2.33972200

C 6.08594000 -0.00054000 3.55158700

C 4.68376300 -0.00057500 3.59019000

C 3.94274200 -0.00039200 2.43064400

C 4.59435100 -0.00017600 1.18593400

C 1.75982900 -1.21689800 -0.00009000

C 0.38446200 -1.21440100 -0.00009900

N -2.43711400 -1.19558100 -0.00012700

C -3.82604700 -3.63395700 -0.00013500

C -4.53005200 -4.82891800 -0.00016000

H -6.47344000 -5.75778800 -0.00026900

H -7.68840600 -3.59567100 -0.00037400

H -6.41171400 -1.45836100 -0.00031800

H -6.41170900 1.45837800 0.00013900

H -7.68839000 3.59569500 0.00042700

H -6.47341300 5.75780600 0.00055900

H -3.99213600 5.77290700 0.00040300

H -2.74202800 3.62184600 0.00011600

H -0.16589700 2.14740800 0.00014000

H 2.30574000 2.15669000 0.00012000

H 2.86124800 0.00029200 -2.44633500

H 4.17217600 0.00058400 -4.54561300

H 6.65474500 0.00062100 -4.47424400

H 7.82968000 0.00037200 -2.27156400

H 7.82963300 -0.00028700 2.27164200

H 6.65465300 -0.00068400 4.47429800

H 4.17208200 -0.00074300 4.54561600

H 2.86119800 -0.00041100 2.44631200

H 2.30573900 -2.15669200 -0.00015100

H -0.16589700 -2.14741000 -0.00017100

H -2.74204400 -3.62184700 -0.00007100

H -3.99216300 -5.77290200 -0.00011400

PXZ-TRZ_S1_LC-PBE_TDA

C 5.84306900 -4.65432000 -1.07567200

C 6.52558000 -3.49206800 -0.79142900

C 5.83290800 -2.33384600 -0.52501600

C 4.45311100 -2.33058300 -0.54058900

C 3.71014600 -1.08732800 -0.25383900

N 4.42341000 -0.00001100 -0.00008900

C 3.71017000 1.08730600 0.25372600

C 4.45316200 2.33053300 0.54052400

C 5.83295800 2.33379200 0.52484300

C 6.52565600 3.49198800 0.79130500

C 5.84317100 4.65421600 1.07571000

C 4.46500500 4.65474000 1.09263600

C 3.77285700 3.49727400 0.82576400

N 2.40435200 1.14284700 0.27045000

C 1.75744900 -0.00002700 0.00010600

C 0.33894800 -0.00003400 0.00019900

C -0.38563100 1.17856600 0.27550700

C -1.73805900 1.19373900 0.27446200

C -2.44795600 -0.00000500 0.00017800

N -3.83518500 0.00003000 0.00006600

C -4.54077400 -0.74446100 0.92073300

C -3.89032900 -1.48737700 1.90100800

C -4.62079900 -2.22525900 2.79140600

C -6.00330300 -2.23189200 2.72966100

C -6.66036900 -1.48625800 1.78291300

C -5.93212500 -0.74501000 0.88564500

O -6.61205700 0.00005800 -0.00022000

C -5.93192800 0.74512500 -0.88593400

C -6.65996900 1.48638900 -1.78335400

C -6.00269100 2.23200900 -2.72996500

C -4.62017500 2.22534700 -2.79142300

C -3.88990600 1.48744800 -1.90087300

C -4.54057100 0.74454900 -0.92073400

C -1.73808000 -1.19377000 -0.27406200

C -0.38565200 -1.17862400 -0.27509700

N 2.40432600 -1.14288900 -0.27035100

C 3.77277900 -3.49734800 -0.82566500

C 4.46490200 -4.65484100 -1.09248700

H 6.38993700 -5.56857600 -1.28631600

H 7.61114100 -3.48827800 -0.77738800

H 6.34746100 -1.40674400 -0.29889500

H 6.34748900 1.40670800 0.29859900

H 7.61121500 3.48819500 0.77717800

H 6.39006000 5.56845000 1.28639600

H 3.92442600 5.56919700 1.31694000

H 2.68891100 3.47186000 0.83305300

H 0.17687100 2.07782900 0.50055800

H -2.28667300 2.09819600 0.52005400

H -2.80869400 -1.45787600 1.94430700

H -4.10815500 -2.80104000 3.55351900

H -6.57585500 -2.81791200 3.44038900

H -7.74212900 -1.45015300 1.72397400

H -7.74174300 1.45030200 -1.72464500

H -6.57508300 2.81803900 -3.44081500

H -4.10735900 2.80111400 -3.55343100

H -2.80826200 1.45792200 -1.94394700

H -2.28670900 -2.09820500 -0.51970500

H 0.17683300 -2.07788400 -0.50020300

H 2.68883300 -3.47193100 -0.83286700

H 3.92430100 -5.56931600 -1.31666200

PXZ-TRZ_S1_LC-PBE0*_TDA

C -5.88287300 4.79806500 0.00001200

C -6.56555900 3.59100300 0.00026600

C -5.86503200 2.39633400 0.00019800

C -4.47489100 2.39927100 -0.00011500

C -3.72792200 1.12300900 -0.00015500

N -4.45629900 -0.00000300 -0.00007800

C -3.72792000 -1.12301300 -0.00016200

C -4.47488500 -2.39927700 -0.00003300

C -5.86502600 -2.39634400 -0.00004100

C -6.56554900 -3.59101400 0.00012600

C -5.88286100 -4.79807400 0.00031200

C -4.49488800 -4.80540300 0.00031300

C -3.79446100 -3.61212000 0.00013400

N -2.41604900 -1.19009700 -0.00031600

C -1.76503800 0.00000100 -0.00033000

C -0.34699800 0.00000200 -0.00039300

C 0.38599400 -1.21733700 -0.00048700

C 1.75250500 -1.22281800 -0.00037800

C 2.44845800 0.00000300 -0.00032000

N 3.87478900 0.00000200 -0.00005500

C 4.56783400 0.00006700 1.18172500

C 3.90638100 0.00017200 2.41546100

C 4.63832300 0.00024500 3.57729100

C 6.03534800 0.00023800 3.54098000

C 6.70478200 0.00015100 2.33559300

C 5.97375400 0.00006700 1.16212400

O 6.65656000 -0.00005700 0.00043300

C 5.97416600 -0.00010000 -1.16149800

C 6.70560400 -0.00016500 -2.33471300

C 6.03659400 -0.00020700 -3.54033600

C 4.63958200 -0.00018900 -3.57713800

C 3.90723900 -0.00012800 -2.41556200

C 4.56825600 -0.00006400 -1.18159300

C 1.75250300 1.22282500 -0.00022200

C 0.38599300 1.21734200 -0.00027600

N -2.41605200 1.19009700 -0.00029100

C -3.79447000 3.61211700 -0.00037400

C -4.49490100 4.80539700 -0.00031300

H -6.43273900 5.73519700 0.00006400

H -7.65190000 3.58102100 0.00052400

H -6.37412300 1.43815100 0.00039500

H -6.37412000 -1.43816200 -0.00017800

H -7.65189100 -3.58103500 0.00011600

H -6.43272400 -5.73520800 0.00045700

H -3.95686000 -5.74930800 0.00045900

H -2.70944900 -3.58874800 0.00013700

H -0.17366000 -2.14647100 -0.00061400

H 2.30297900 -2.16033700 -0.00041700

H 2.82271000 0.00020400 2.41236400

H 4.12332400 0.00031400 4.53122800

H 6.60122400 0.00030800 4.46612300

H 7.78735300 0.00012900 2.27442600

H 7.78815400 -0.00016900 -2.27316900

H 6.60279500 -0.00026000 -4.46528000

H 4.12491700 -0.00022900 -4.53125600

H 2.82356700 -0.00014200 -2.41282500

H 2.30297900 2.16034400 -0.00010200

H -0.17366300 2.14647500 -0.00017300

H -2.70945800 3.58874700 -0.00061900

H -3.95687500 5.74930400 -0.00051800

CC2TA_T1_PBE0_TDA

C -1.11539000 2.46954200 0.05920200

N -0.55194300 3.59463500 -0.24825400

C 0.81481500 3.66905200 -0.11198600

N 1.53497400 2.57340100 0.34100700

C 0.82353000 1.52583100 0.57383200

N -0.49179700 1.33347800 0.47783300

C 1.49597100 4.86955700 -0.44037300

C 0.80805500 5.97604400 -1.00410900

C 1.47573100 7.14106900 -1.32146800

C 2.84889800 7.27095200 -1.09553200

C 3.54068200 6.19173800 -0.53929700

C 2.89032300 5.01727600 -0.21940600

N 1.55229300 0.34805900 1.00447800

N -2.51352100 2.38380900 -0.02450500

C -3.26801000 1.20869000 -0.12437400

C -4.63562000 1.55236500 -0.17206300

C -4.71759300 2.98734000 -0.09391200

C -3.39792100 3.47369100 -0.01278800

C 2.80059100 0.01387600 0.59820000

C 3.18408100 -1.21348200 1.22462100

C 2.08404300 -1.60286700 2.07000100

C 1.08620000 -0.62261300 1.90169300

C -2.87450100 -0.12665700 -0.21783200

C -3.85890100 -1.09590400 -0.32791400

C -5.21780000 -0.76044000 -0.37006200

C -5.61034700 0.56770100 -0.30171200

C -5.79809100 3.86691000 -0.07450700

C -5.55362900 5.22484900 0.03388000

C -4.24063000 5.69682100 0.12630400

C -3.14917200 4.84064700 0.10318100

C 1.87835500 -2.67525700 2.93105800

C 0.67523900 -2.75430200 3.61919700

C -0.30381300 -1.77398300 3.44615000

C -0.11992300 -0.69422600 2.59032800

C 3.66472800 0.66797900 -0.30097200

C 4.89393700 0.10762100 -0.54069100

C 5.28194300 -1.10450500 0.07834000

C 4.41503100 -1.77021400 0.96012200

N -6.19145000 -1.78521400 -0.48244800

N 6.53596100 -1.64383200 -0.19041100

C -6.31541200 -2.67070100 -1.54101000

C -7.38729600 -3.55413700 -1.28208600

C -7.93371100 -3.17572700 -0.00280200

C -7.16644800 -2.08185700 0.45689100

C 7.73893400 -0.93327400 -0.23728600

C 8.78634000 -1.83038900 -0.52707500

C 8.19047400 -3.14422600 -0.66374000

C 6.80580100 -2.98981800 -0.45157200

C -5.57463500 -2.74473300 -2.71806800

C -5.91664100 -3.72933700 -3.63227200

C -6.97017400 -4.61955100 -3.38883100

C -7.70909100 -4.53500600 -2.21978300

C -8.97816900 -3.65973200 0.78451100

C -9.23854700 -3.05788300 2.00505900

C -8.46190400 -1.97994500 2.44828900

C -7.41820000 -1.47773300 1.68622400

C 8.70182600 -4.39411800 -0.97681100

C 7.82506700 -5.47065500 -1.08183300

C 6.45177900 -5.29629000 -0.90460800

C 5.91753700 -4.05158400 -0.59919000

C 7.97733300 0.41001600 0.03965800

C 9.28984200 0.85578800 -0.03761600

C 10.33305300 -0.01477600 -0.35669100

C 10.09093200 -1.36570700 -0.59020500

H -0.25366700 5.88105300 -1.20095600

H 0.92107600 7.96802300 -1.75725500

H 3.36636100 8.19136200 -1.34499800

H 4.60743000 6.27840800 -0.34814200

H 3.43347000 4.19522100 0.23271600

H -1.82939100 -0.39512900 -0.18366100

H -3.57763200 -2.14300500 -0.37677000

H -6.66326200 0.82494800 -0.36065800

H -6.81473700 3.49018700 -0.13946600

H -6.38172500 5.92653900 0.04866000

H -4.06260400 6.76443500 0.21411300

H -2.13751600 5.21298400 0.16118000

H 2.64309400 -3.43390500 3.06888700

H 0.49391800 -3.58168500 4.29701300

H -1.23952500 -1.85397500 3.98963700

H -0.88660700 0.05177000 2.43893800

H 3.35687800 1.58734400 -0.77677100

H 5.55791800 0.56450300 -1.26511000

H 4.74451700 -2.66995500 1.46760300

H -4.76405000 -2.05098600 -2.91393500

H -5.35637000 -3.80709600 -4.55927300

H -7.21094400 -5.37844700 -4.12669600

H -8.53096600 -5.22161600 -2.03779100

H -9.57905100 -4.49898000 0.44591300

H -10.05026700 -3.42375200 2.62597800

H -8.67982600 -1.52568300 3.41026900

H -6.81642400 -0.64517500 2.03470500

H 9.76399200 -4.53214100 -1.15009000

H 8.21136800 -6.45454200 -1.32476000

H 5.78453300 -6.14317000 -1.02124300

H 4.84680900 -3.91600600 -0.50736700

H 7.17948900 1.08459500 0.32446100

H 9.50518000 1.89889200 0.16541700

H 11.34810500 0.36397900 -0.40597800

H 10.91174900 -2.04170700 -0.80606700

CC2TA_T1_LC-PBE_TDA

C -1.12953500 2.37354900 0.00022500

N -0.53373000 3.51856600 -0.27615900

C 0.78090100 3.49769200 -0.21709000

N 1.50072600 2.44214200 0.10801500

C 0.80977400 1.35435700 0.38293000

N -0.50153000 1.26270300 0.33550600

C 1.49976700 4.73821600 -0.53643100

C 0.84415700 5.77371500 -1.17235800

C 1.52020600 6.93089000 -1.47769900

C 2.84979400 7.06118400 -1.14010600

C 3.50521600 6.03108100 -0.50156100

C 2.83365300 4.86899700 -0.20448900

N 1.51105600 0.23544300 0.74214500

N -2.48800600 2.33362900 -0.06972800

C -3.27273800 1.17057200 -0.10208600

C -4.64625500 1.58695200 -0.14121400

C -4.68556300 2.95711400 -0.14938600

C -3.34512600 3.45184100 -0.11053500

C 2.86049800 -0.00922600 0.46327200

C 3.18547100 -1.27220800 0.93876900

C 2.00565000 -1.81857900 1.55446300

C 0.99542900 -0.87719600 1.41913600

C -2.93916300 -0.13977200 -0.15924600

C -3.98215300 -1.08868200 -0.21395400

C -5.33355700 -0.69965200 -0.24456500

C -5.68246600 0.61186700 -0.21309500

C -5.77650300 3.87329200 -0.18146500

C -5.50614900 5.20780500 -0.14984300

C -4.19414700 5.66772300 -0.09144500

C -3.09007000 4.77887300 -0.07512000

C 1.77463200 -3.00926000 2.21117200

C 0.53127100 -3.24173400 2.73985700

C -0.46340700 -2.28507200 2.62417400

C -0.25174700 -1.09493700 1.97068600

C 3.79741800 0.75386400 -0.20569300

C 5.05277500 0.22637300 -0.37765600

C 5.39031000 -1.02381100 0.11738400

C 4.45658500 -1.77943900 0.78166600

N -6.31713900 -1.69531200 -0.28992700

N 6.69073000 -1.51862400 -0.07181000

C -6.38320400 -2.71355500 -1.21626100

C -7.48279700 -3.51784300 -0.93197800

C -8.11725100 -2.95337300 0.22834400

C -7.36977300 -1.83583700 0.58953900

C 7.85061100 -0.91342700 0.35518700

C 8.93877200 -1.69843000 -0.01686300

C 8.40441800 -2.84221100 -0.70479400

C 7.02044300 -2.68939900 -0.71626100

C -5.56780200 -2.95920000 -2.30459200

C -5.86512900 -4.04466700 -3.08849500

C -6.94915000 -4.86652100 -2.80940300

C -7.76306800 -4.60505900 -1.73752200

C -9.22438600 -3.30884200 0.97495600

C -9.56255100 -2.55160100 2.06655200

C -8.79824300 -1.44797000 2.42183900

C -7.69636900 -1.07449500 1.69546500

C 8.97550000 -3.94876300 -1.30467700

C 8.16232900 -4.87505500 -1.90476400

C 6.78417600 -4.70119900 -1.91687300

C 6.19381000 -3.61242100 -1.32818300

C 8.01208900 0.26111700 1.06514100

C 9.29133300 0.64057400 1.38148100

C 10.38899800 -0.12457800 1.00813200

C 10.21852000 -1.29424100 0.31366400

H -0.20040500 5.64968700 -1.43746400

H 1.00600900 7.73989800 -1.98641700

H 3.38216300 7.97679900 -1.37852300

H 4.55125400 6.13550900 -0.23220900

H 3.33126200 4.04874200 0.30190600

H -1.91036200 -0.46642500 -0.14955500

H -3.74466800 -2.14636100 -0.20487300

H -6.72381900 0.90834900 -0.26924800

H -6.79475800 3.50317000 -0.22524100

H -6.32008600 5.92522600 -0.16927000

H -4.00111500 6.73411900 -0.05960600

H -2.07936400 5.15516900 -0.03551000

H 2.56759500 -3.74382500 2.31299100

H 0.32660500 -4.17159300 3.26002500

H -1.43871900 -2.47307200 3.06189500

H -1.03658800 -0.35628100 1.89578900

H 3.54723200 1.73180600 -0.58967500

H 5.80523300 0.79070700 -0.91946800

H 4.72852500 -2.75468700 1.17347200

H -4.72969200 -2.31115600 -2.53809200

H -5.24082800 -4.26158400 -3.94965600

H -7.15503800 -5.71679900 -3.45116100

H -8.61806000 -5.24037700 -1.52635600

H -9.81531500 -4.17810100 0.70213700

H -10.42953400 -2.81669300 2.66260700

H -9.07631700 -0.86746900 3.29603300

H -7.09733500 -0.21782600 1.98499100

H 10.05345000 -4.08048400 -1.30273700

H 8.59533500 -5.74945700 -2.37930000

H 6.15905700 -5.44172400 -2.40616800

H 5.11780400 -3.47495100 -1.35060200

H 7.15670600 0.85547900 1.36888400

H 9.44824100 1.55821900 1.93996100

H 11.38732900 0.20649800 1.27458700

H 11.07623700 -1.89668500 0.02957600

CC2TA_T1_LC-PBE0*_TDA

C -1.15078200 2.37751600 -0.00442900

N -0.55452300 3.53809500 -0.26451100

C 0.76932500 3.51171100 -0.19884600

N 1.49882300 2.44332500 0.11924900

C 0.79382800 1.34812500 0.37543800

N -0.52547700 1.24597200 0.32014500

C 1.49084200 4.75524400 -0.49854300

C 0.82353300 5.82879200 -1.08232300

C 1.50731000 6.99658600 -1.36949000

C 2.85833300 7.10261300 -1.06915700

C 3.52622100 6.03599600 -0.48448700

C 2.84714100 4.86354500 -0.20325000

N 1.49911700 0.21837600 0.72568200

N -2.51682800 2.33842100 -0.08244500

C -3.29983200 1.18238900 -0.11594700

C -4.69821900 1.60534600 -0.16342800

C -4.72633000 2.98527900 -0.18472300

C -3.37956900 3.46870500 -0.13450000

C 2.85723400 -0.01385900 0.45860600

C 3.18370600 -1.30076000 0.91190300

C 1.99652600 -1.86622600 1.49693400

C 0.97466400 -0.91395700 1.36983400

C -2.95941200 -0.14304200 -0.16861900

C -3.99291200 -1.08908300 -0.24317000

C -5.37412200 -0.68759700 -0.27455800

C -5.72844900 0.62742900 -0.22914200

C -5.80361300 3.91617600 -0.22991400

C -5.51760500 5.26149600 -0.20013200

C -4.19867600 5.70914900 -0.13019700

C -3.10447700 4.80330300 -0.10014500

C 1.75405500 -3.08009000 2.12517700

C 0.49297800 -3.32753200 2.63309700

C -0.50741800 -2.36352200 2.52726900

C -0.28720200 -1.14720000 1.90147600

C 3.80539300 0.77573600 -0.17954300

C 5.07582200 0.25481600 -0.34792700

C 5.41519300 -1.01474900 0.12351700

C 4.46818600 -1.79950700 0.75981900

N -6.33821500 -1.69427800 -0.30272800

N 6.72267500 -1.49820000 -0.05796300

C -6.30647100 -2.81292100 -1.12433900

C -7.42478400 -3.61760100 -0.84543500

C -8.17193800 -2.94746400 0.18773900

C -7.47596200 -1.76407700 0.49278100

C 7.88313800 -0.84736900 0.32070800

C 8.98989900 -1.64478000 -0.02648000

C 8.46497900 -2.83480700 -0.64180200

C 7.06334100 -2.70489600 -0.64284100

C -5.40644800 -3.13859600 -2.13082700

C -5.62025400 -4.31850400 -2.82223500

C -6.70947600 -5.14255800 -2.53800700

C -7.62130500 -4.79300700 -1.55802400

C -9.33632400 -3.25967100 0.87711000

C -9.78447200 -2.39698600 1.86079300

C -9.07101700 -1.23773400 2.16649700

C -7.90972800 -0.90322100 1.49182100

C 9.04893500 -3.96969900 -1.19312300

C 8.23586300 -4.94642900 -1.73668700

C 6.84762300 -4.79416300 -1.74198700

C 6.24069000 -3.67591200 -1.19925400

C 8.03577300 0.37018200 0.97144000

C 9.32409300 0.78752900 1.25319900

C 10.43451100 0.01641500 0.90268800

C 10.27411900 -1.20091300 0.26789400

H -0.22993700 5.72261500 -1.32133600

H 0.98626200 7.82856000 -1.83289000

H 3.39331300 8.02067600 -1.29294200

H 4.58185800 6.12015800 -0.24589700

H 3.34956900 4.02129000 0.26183900

H -1.92754800 -0.46181500 -0.14270700

H -3.76031400 -2.14662700 -0.19879200

H -6.77006500 0.92289200 -0.29451500

H -6.82708300 3.55966200 -0.28375900

H -6.32578900 5.98563800 -0.23089400

H -3.99155400 6.77325200 -0.09880400

H -2.08661000 5.16159800 -0.05020600

H 2.54658300 -3.81656600 2.22148100

H 0.28217700 -4.27067200 3.12687900

H -1.48874200 -2.56225500 2.94713000

H -1.06988500 -0.40434700 1.83562500

H 3.55220800 1.76206400 -0.54178500

H 5.83071900 0.83618700 -0.86837700

H 4.74050400 -2.78126900 1.13602900

H -4.57710600 -2.48511200 -2.38066800

H -4.92967100 -4.59983500 -3.61115700

H -6.84886100 -6.05851800 -3.10315300

H -8.48214000 -5.42334000 -1.35439800

H -9.88201900 -4.17117300 0.65078800

H -10.69445400 -2.62518800 2.40637100

H -9.43146200 -0.58088900 2.95214700

H -7.35226800 -0.00839000 1.74659000

H 10.12926200 -4.08419500 -1.19972600

H 8.67764900 -5.83849500 -2.16920500

H 6.22827900 -5.56837600 -2.18477600

H 5.16207800 -3.55576400 -1.21889900

H 7.17513900 0.96610800 1.25841500

H 9.47277000 1.73504900 1.76230000

H 11.43115800 0.37628500 1.13769500

H 11.13826900 -1.80507300 0.00619300

DMOC-DPS_T1_PBE0_TDA

C -5.07004800 6.04956600 1.55320800

O -3.93120500 5.21138800 1.49964100

C -4.07970200 3.92374000 1.17917300

C -2.88195600 3.17379800 1.15069300

C -2.88649900 1.83742300 0.83818900

C -4.12184200 1.24165500 0.54936700

C -5.33119300 1.98222600 0.57095600

C -5.31908400 3.32607800 0.88456100

C -6.37921100 1.04044600 0.21284900

C -7.74142700 1.13074600 0.06078000

C -8.46205900 -0.03048200 -0.29885400

O -9.78168900 0.15005100 -0.42776800

C -10.59357500 -0.95366500 -0.78377500

C -7.80680200 -1.25579200 -0.49343600

C -6.43273400 -1.35161000 -0.33833200

C -5.72935700 -0.20308500 0.01074300

N -4.37952900 -0.05628000 0.21551600

C -3.40449600 -1.09955100 0.09997700

C -2.88743200 -1.70789900 1.26663800

C -1.98201700 -2.73044400 1.15856100

C -1.56799000 -3.19067000 -0.12451500

S -0.23646700 -4.28399700 -0.25892600

O -0.29303000 -4.91470000 -1.57923000

O -0.16872500 -5.07606000 0.97071900

C 1.21473000 -3.25513900 -0.26432100

C 1.78844500 -2.85877900 0.94658300

C 2.81825500 -1.93351300 0.95118100

C 3.29574500 -1.40579200 -0.25087300

N 4.34825600 -0.46153700 -0.24465600

C 4.29748100 0.81393400 -0.78644900

C 3.24864300 1.46590200 -1.43485600

C 3.45628800 2.75945600 -1.87057700

C 4.68402900 3.41598700 -1.66963200

O 4.74254800 4.68726600 -2.15772400

C 5.94804500 5.38684700 -1.99864100

C 5.72440900 2.77544900 -1.01498700

C 5.52826200 1.46345100 -0.56712300

C 6.36340000 0.53366600 0.14482300

C 7.67849800 0.60145100 0.62122400

C 8.20860600 -0.50635500 1.26351200

O 9.46845300 -0.57391000 1.77642000

C 10.28651700 0.55704800 1.63795300

C 7.44045300 -1.67461200 1.42219100

C 6.14356200 -1.75581400 0.95740100

C 5.60209100 -0.63765800 0.32283800

C 2.73350900 -1.82104000 -1.46033400

C 1.68986200 -2.73076900 -1.46900500

C -2.09817000 -2.56940700 -1.29225500

C -3.00724400 -1.55081300 -1.17971700

H -5.56178000 6.10946200 0.57767400

H -5.78024400 5.70199400 2.30944800

H -4.69805600 7.03259700 1.83129600

H -1.95980200 3.69346100 1.38253300

H -1.97571400 1.25228100 0.81049000

H -6.23735100 3.90041600 0.90006100

H -8.29370800 2.05279200 0.20593400

H -11.60977000 -0.56913800 -0.82789500

H -10.53486500 -1.74466400 -0.03045900

H -10.31057600 -1.35088300 -1.76291900

H -8.36693400 -2.14124800 -0.76478700

H -5.91170500 -2.29056700 -0.48075200

H -3.23085500 -1.38309300 2.24459000

H -1.60105800 -3.22556800 2.04426200

H 1.42928700 -3.29455900 1.87224700

H 3.26224600 -1.61062200 1.88766500

H 2.29490200 0.97442100 -1.59395300

H 2.67172600 3.30459400 -2.38474500

H 5.79570100 6.36316400 -2.45752200

H 6.78074600 4.87991100 -2.50197200

H 6.20165100 5.52408000 -0.93988800

H 6.67499000 3.26743500 -0.84317200

H 8.25855900 1.50606000 0.47852900

H 11.23732600 0.30305100 2.10509800

H 10.46012700 0.80569500 0.58344700

H 9.85968500 1.43134100 2.14543700

H 7.90403500 -2.51884500 1.92130300

H 5.56975300 -2.66826300 1.07730700

H 3.13767300 -1.43936600 -2.39270000

H 1.26241800 -3.07735400 -2.40329600

H -1.79971400 -2.93778700 -2.26694300

H -3.42509000 -1.09027600 -2.07030900

DMOC-DPS_T1_LC-PBE_TDA

C -6.67657100 5.82597100 0.05531900

O -5.47002700 5.27330300 -0.37561300

C -5.31984800 3.95451800 -0.28594900

C -4.04667200 3.50189500 -0.75120900

C -3.72888000 2.16102500 -0.73736600

C -4.67362600 1.28559100 -0.26724600

C -5.95351900 1.73183100 0.23303600

C -6.26469300 3.10620900 0.20021700

C -6.64249700 0.60509400 0.67576600

C -7.91553000 0.41570700 1.23090900

C -8.33236000 -0.90169100 1.53479400

O -9.56295400 -0.97287900 2.06363400

C -10.05217600 -2.23541600 2.38529600

C -7.53046800 -1.97120900 1.29432400

C -6.23062600 -1.78056200 0.72744600

C -5.82355500 -0.51336800 0.45309400

N -4.63193400 -0.06684400 -0.11854600

C -3.55740000 -0.88666100 -0.47668700

C -3.04262500 -1.78115900 0.44081900

C -1.99041900 -2.58971000 0.08839600

C -1.45792800 -2.48520100 -1.17546800

S -0.10230600 -3.50793600 -1.62193400

O -0.10897600 -3.62120500 -3.05717200

O -0.13457000 -4.65832500 -0.75665500

C 1.30517300 -2.56083500 -1.17875100

C 1.83663900 -2.69441900 0.08294700

C 2.92896500 -1.94095600 0.43398700

C 3.49383800 -1.07312700 -0.48220300

N 4.60747300 -0.31541600 -0.12826200

C 4.76067500 1.04432300 -0.32487400

C 3.87622100 1.97141400 -0.85233100

C 4.27069400 3.27611500 -0.90604100

C 5.52197300 3.68205700 -0.43961700

O 5.77867700 4.99778700 -0.56103300

C 7.00940800 5.45646500 -0.10483800

C 6.38830900 2.77042400 0.10332600

C 5.99580800 1.43976700 0.16228000

C 6.63298500 0.26300600 0.68129000

C 7.87444900 0.04904900 1.26558300

C 8.20940900 -1.22669600 1.63502600

O 9.37504700 -1.57050600 2.21352300

C 10.29740600 -0.55754300 2.45063900

C 7.32409100 -2.28231200 1.41084200

C 6.10275200 -2.08220400 0.83749100

C 5.75240400 -0.78836900 0.48583700

C 2.96127400 -0.96465600 -1.75385300

C 1.85964900 -1.70516100 -2.10256100

C -1.96827600 -1.60409700 -2.10039400

C -3.02662700 -0.80338500 -1.74890400

H -7.51761100 5.43381700 -0.52440100

H -6.84317100 5.62562900 1.11802100

H -6.59741600 6.89924800 -0.10236300

H -3.35297500 4.25390800 -1.10495900

H -2.75782200 1.81883800 -1.07706800

H -7.22414100 3.45384600 0.56027600

H -8.59577100 1.23401100 1.43280300

H -11.04932400 -2.08711700 2.79472800

H -9.42295900 -2.72599700 3.13559500

H -10.11738700 -2.87409500 1.49801200

H -7.85174600 -2.97955200 1.51963400

H -5.60911400 -2.64131100 0.50859700

H -3.47080200 -1.82756300 1.43631900

H -1.58488000 -3.31679100 0.78426600

H 1.39515300 -3.39873600 0.78045900

H 3.34713400 -2.00652100 1.43270100

H 2.89122300 1.68194700 -1.20215800

H 3.61676200 4.04136400 -1.30992700

H 7.02937100 6.52841500 -0.29091500

H 7.83842000 4.98456400 -0.64406400

H 7.13117700 5.27822500 0.96945900

H 7.35776600 3.06538100 0.48766600

H 8.55320000 0.88105500 1.41262200

H 11.16000700 -1.02689300 2.91938500

H 10.61490900 -0.07738900 1.51824100

H 9.89266300 0.20330000 3.12743900

H 7.64697400 -3.27520800 1.70450900

H 5.44071600 -2.92120400 0.65250500

H 3.44263400 -0.31571100 -2.47764800

H 1.44900900 -1.65904800 -3.10599200

H -1.55717500 -1.58125900 -3.10435800

H -3.47123600 -0.12211300 -2.46649100

DMOC-DPS_T1_LC-PBE0*_TDA

C -6.68102700 5.84843200 0.12125500

O -5.47417300 5.31020600 -0.34843600

C -5.30983700 3.98684600 -0.27451500

C -4.05911500 3.53711000 -0.76768400

C -3.75172200 2.18422100 -0.75858100

C -4.70514000 1.31314400 -0.26399300

C -5.96790500 1.75585600 0.26544700

C -6.26722800 3.12799000 0.24299000

C -6.65462400 0.61436800 0.72549500

C -7.91497700 0.40553900 1.30857700

C -8.30528800 -0.93156200 1.61094200

O -9.52284900 -1.01836000 2.16726600

C -9.98697500 -2.29816700 2.49184500

C -7.49738500 -2.00873400 1.34436300

C -6.21739400 -1.79727800 0.74929100

C -5.83318000 -0.50259400 0.47767800

N -4.66004000 -0.04691200 -0.11836200

C -3.58736700 -0.86237600 -0.50377400

C -3.07100800 -1.78866300 0.39493100

C -2.01492600 -2.59380800 0.01430900

C -1.48565200 -2.45445800 -1.25700000

S -0.12486100 -3.47672900 -1.73990900

O -0.13538000 -3.54196100 -3.18901700

O -0.16255500 -4.65877600 -0.89972500

C 1.29263600 -2.54035100 -1.26516600

C 1.82675700 -2.71610700 0.00002900

C 2.92727900 -1.97217100 0.37648600

C 3.49721500 -1.06907600 -0.51788000

N 4.61155700 -0.32426200 -0.14065400

C 4.79233400 1.03571100 -0.35572500

C 3.93342400 1.97184400 -0.92072000

C 4.35255700 3.28095300 -0.97872900

C 5.60098700 3.67486400 -0.47928800

O 5.88332900 4.98878600 -0.61023600

C 7.11649900 5.42005200 -0.11639200

C 6.44510400 2.75050300 0.10658800

C 6.03023000 1.42151800 0.17053000

C 6.63171900 0.24161300 0.72881000

C 7.85633000 0.01443600 1.35430100

C 8.16148900 -1.27330300 1.75268600

O 9.30748400 -1.62745500 2.37267600

C 10.23051100 -0.60886600 2.61963000

C 7.26448200 -2.32278300 1.51324900

C 6.05327800 -2.10957000 0.89668400

C 5.73231000 -0.80993700 0.52040900

C 2.95931200 -0.91828600 -1.79423000

C 1.84981200 -1.64996800 -2.16766800

C -1.99846600 -1.54222600 -2.16314700

C -3.05927800 -0.74273300 -1.78410700

H -7.53450700 5.45249100 -0.44000100

H -6.81826800 5.63837500 1.18769900

H -6.61182800 6.92410000 -0.03178300

H -3.36572400 4.28211500 -1.13847700

H -2.78981800 1.82900900 -1.11386000

H -7.21293000 3.48946800 0.62849000

H -8.60587600 1.20972700 1.53323800

H -10.97598100 -2.16378000 2.92729200

H -9.32798400 -2.78435700 3.22098400

H -10.06230100 -2.92955500 1.59859800

H -7.81005800 -3.02042600 1.56995900

H -5.58851400 -2.64389200 0.49518200

H -3.49702600 -1.85783700 1.39085500

H -1.60627100 -3.34138100 0.68741800

H 1.38193500 -3.44145700 0.67472900

H 3.34279300 -2.06729400 1.37471600

H 2.95248900 1.69360800 -1.29290000

H 3.72004100 4.04970800 -1.41113300

H 7.16816700 6.49017000 -0.31262000

H 7.95059400 4.91917400 -0.62466900

H 7.19982500 5.24737200 0.96443000

H 7.40676000 3.03882400 0.51841800

H 8.54673500 0.83702500 1.50987000

H 11.07598500 -1.07644400 3.12248900

H 10.57729000 -0.14521900 1.68704100

H 9.80789100 0.16764600 3.27031900

H 7.56177100 -3.31842100 1.82683200

H 5.38587200 -2.94231400 0.69860200

H 3.44400700 -0.25070500 -2.49972700

H 1.43801200 -1.57358600 -3.16961500

H -1.58931100 -1.49621200 -3.16789100

H -3.50809800 -0.04147700 -2.48059600

PIC-TRZ_T1_PBE0_TDA

C -12.14144500 -0.03826600 -0.02404200

C -11.42566400 -1.12636400 -0.51276700

C -10.03901500 -1.11706300 -0.50770200

C -9.31593900 -0.02127900 -0.00855200

C -7.85089500 -0.01277500 -0.00053300

C -7.10384600 -1.20158900 0.13039800

C -5.72947200 -1.20316400 0.14045900

C -4.98994500 0.00341500 0.01446200

C -3.57554900 0.00776000 0.02228900

N -2.88846600 -1.18304100 0.05737700

C -1.59459300 -1.10929300 0.07118500

N -0.85836100 -2.30549400 0.04901700

C -1.32140500 -3.53643300 -0.44398900

C -2.55591000 -3.88297600 -0.98819100

C -2.72198400 -5.18586400 -1.43868100

C -1.69748500 -6.13201900 -1.35227900

C -0.46984600 -5.78181300 -0.81394100

C -0.27754500 -4.47870000 -0.36168100

C 0.85996300 -3.80841500 0.21718700

C 2.13568500 -4.24995600 0.53894200

C 3.01897300 -3.34921200 1.12182400

C 4.39563200 -3.43953400 1.54388500

C 5.33532900 -4.46882100 1.54359700

C 6.60265500 -4.23040500 2.05553600

C 6.93216200 -2.97702800 2.57995200

C 6.01476300 -1.93453600 2.59292400

C 4.75190300 -2.17288400 2.05853400

N 3.65494600 -1.32227200 1.96165000

C 3.61309000 0.02163100 2.38706600

C 4.58897200 0.92510600 1.96431300

C 4.54524700 2.24727000 2.38693800

C 3.52060700 2.68446800 3.21816500

C 2.54475100 1.78606100 3.63483900

C 2.59260500 0.45858600 3.23174800

C 2.59416000 -2.02531600 1.38967300

C 1.32887100 -1.55186100 1.06563200

C 0.46978500 -2.46487700 0.46160500

N -0.84574100 0.02167900 0.08657900

C -1.61281100 1.11298800 0.03933500

N -0.86531300 2.34592300 0.03813100

C -1.30418100 3.55665100 0.50998000

C -2.52275700 3.90718300 1.10667700

C -2.67319200 5.21276700 1.51759100

C -1.65798300 6.18482400 1.35086600

C -0.46000700 5.85124800 0.76835500

C -0.26693400 4.52777000 0.34330300

C 0.82470400 3.86038500 -0.26271000

C 2.10171100 4.30718000 -0.65419600

C 2.96009200 3.40072800 -1.23376500

C 4.32129400 3.46582800 -1.72427000

C 5.26634000 4.47704600 -1.82905600

C 6.50692100 4.18025200 -2.38586300

C 6.80448600 2.89438500 -2.84402100

C 5.87499000 1.86699400 -2.75354400

C 4.64520000 2.17072400 -2.18239000

N 3.54361500 1.33958400 -1.98957800

C 3.48328000 -0.04073900 -2.32552200

C 2.50763900 -0.48822200 -3.21179200

C 2.42787100 -1.84219200 -3.50540400

C 3.31763900 -2.73699900 -2.92306700

C 4.29508200 -2.28022200 -2.04623900

C 4.38287500 -0.92921400 -1.74368600

C 2.52949300 2.05708000 -1.41907300

C 1.27098100 1.57425900 -1.02706900

C 0.43188000 2.50220000 -0.44160600

N -2.89657800 1.21419400 -0.00783700

C -5.74192600 1.20107600 -0.11933900

C -7.11650600 1.18441100 -0.12401100

C -10.05765200 1.06559600 0.48265000

C -11.44431200 1.05831300 0.47255100

H -13.22710100 -0.04499800 -0.02971200

H -11.95322400 -1.98745100 -0.91385200

H -9.50091200 -1.96128900 -0.92785800

H -7.62690800 -2.14367900 0.26974900

H -5.18797400 -2.13198100 0.27989700

H -3.35661900 -3.16027200 -1.03991600

H -3.68021200 -5.47288200 -1.86105600

H -1.86668200 -7.14459300 -1.70525700

H 0.33148200 -6.51213900 -0.74444700

H 2.43183500 -5.27674400 0.34362700

H 5.07464300 -5.45051600 1.15832000

H 7.34124100 -5.02567500 2.06202200

H 7.92258100 -2.81363800 2.99360000

H 6.27370300 -0.97301900 3.02253600

H 5.37942700 0.58780900 1.30192600

H 5.31201400 2.94002400 2.05376700

H 3.48461800 3.71904100 3.54427400

H 1.74521400 2.11539600 4.29093900

H 1.84690100 -0.24939100 3.57646600

H 1.01977100 -0.53531100 1.25382900

H -3.30866000 3.17476900 1.21301800

H -3.60836700 5.50835900 1.98190800

H -1.83581500 7.20048800 1.68771900

H 0.32276600 6.59198100 0.63928400

H 2.39402300 5.34002100 -0.49327200

H 5.04483500 5.48392000 -1.48974700

H 7.25398300 4.96209700 -2.47260200

H 7.77580000 2.69423700 -3.28324300

H 6.09589400 0.87093400 -3.11966700

H 1.82485600 0.22116000 -3.66753800

H 1.66287400 -2.19844800 -4.18666100

H 3.24003100 -3.79610700 -3.14343400

H 4.97466000 -2.97760700 -1.56893600

H 5.11764400 -0.56933400 -1.03180400

H 0.98172100 0.53930100 -1.13426700

H -5.21108000 2.13643600 -0.25582700

H -7.64956400 2.11992800 -0.27027300

H -9.53432400 1.91571500 0.90969600

H -11.98676200 1.91252700 0.86854200

PIC-TRZ_T1_LC-PBE_TDA

C 12.03248500 -0.00011800 0.00190800

C 11.32914300 -1.19372500 -0.08404900

C 9.96729600 -1.20157400 -0.08626700

C 9.21547600 0.00000700 0.00034800

C 7.81076400 0.00006300 -0.00033900

C 7.04234000 -1.21593700 -0.13094300

C 5.70097000 -1.21462600 -0.12781000

C 4.97193500 0.00008300 -0.00084200

C 3.53985800 0.00006400 -0.00076500

N 2.91991700 -1.17612000 0.01726200

C 1.60760800 -1.11618800 0.01354100

N 0.90369100 -2.29022000 0.01827200

C 1.39425500 -3.54602400 0.40211600

C 2.63190100 -3.91449300 0.88611500

C 2.82206500 -5.23337700 1.22834900

C 1.81327800 -6.17016600 1.09674300

C 0.57982600 -5.79345400 0.62602300

C 0.36940100 -4.47549600 0.28301800

C -0.79452800 -3.78155300 -0.20536600

C -2.05704100 -4.21944400 -0.52055300

C -2.96065000 -3.30018700 -1.00363200

C -4.33985900 -3.37719500 -1.40202000

C -5.25905300 -4.40692300 -1.44964300

C -6.52954200 -4.14768900 -1.89866500

C -6.88122500 -2.87062600 -2.31285100

C -5.98474900 -1.83176700 -2.27631700

C -4.71342100 -2.09619800 -1.80440100

N -3.63680900 -1.24764000 -1.66988400

C -3.60669000 0.11942300 -1.99371500

C -4.57369100 0.97653300 -1.51121700

C -4.51794700 2.31721900 -1.81351500

C -3.48901300 2.81219500 -2.58247900

C -2.52792600 1.95526200 -3.06925200

C -2.59193500 0.61024400 -2.78907800

C -2.57187200 -1.96838300 -1.17673700

C -1.31515900 -1.50322100 -0.85497700

C -0.43950400 -2.43635100 -0.35642700

N 0.90609900 0.00003800 -0.00048400

C 1.60758600 1.11627600 -0.01468600

N 0.90363900 2.29028900 -0.01926400

C 1.39406100 3.54610500 -0.40324800

C 2.63155700 3.91460400 -0.88760700

C 2.82158900 5.23349600 -1.22988500

C 1.81281900 6.17026100 -1.09797200

C 0.57951400 5.79351800 -0.62689300

C 0.36922000 4.47555200 -0.28383700

C -0.79454600 3.78157800 0.20489500

C -2.05696900 4.21943500 0.52049400

C -2.96039800 3.30015000 1.00386000

C -4.33947800 3.37712200 1.40270400

C -5.25868100 4.40682800 1.45064200

C -6.52901100 4.14756200 1.90009300

C -6.88052500 2.87048900 2.31439100

C -5.98403700 1.83165000 2.27754800

C -4.71287500 2.09611400 1.80520500

N -3.63628800 1.24758300 1.67032600

C -3.60602500 -0.11947800 1.99415300

C -2.59099400 -0.61027100 2.78918300

C -2.52685900 -1.95528600 3.06934100

C -3.48808700 -2.81224500 2.58289100

C -4.51729000 -2.31729800 1.81427000

C -4.57316700 -0.97661400 1.51198400

C -2.57153200 1.96835400 1.17682700

C -1.31491300 1.50322500 0.85465300

C -0.43944400 2.43638500 0.35583500

N 2.91989200 1.17623500 -0.01870900

C 5.70094100 1.21480100 0.12618600

C 7.04230900 1.21610000 0.12975100

C 9.96730700 1.20152700 0.08773500

C 11.32915400 1.19355500 0.08704500

H 13.11705500 -0.00016700 0.00252200

H 11.86802600 -2.13362100 -0.14955000

H 9.46024800 -2.15550500 -0.15008800

H 7.55291400 -2.16297900 -0.24774400

H 5.14184800 -2.13582700 -0.24471900

H 3.42405300 -3.18779400 0.98989300

H 3.79056400 -5.54090200 1.61016100

H 1.99752000 -7.20380400 1.37108500

H -0.22103700 -6.52022500 0.52845800

H -2.33099100 -5.26294900 -0.39488400

H -4.97815000 -5.40946700 -1.14003900

H -7.26304500 -4.94611400 -1.94151300

H -7.88643600 -2.68790700 -2.67982500

H -6.26211800 -0.84106900 -2.61964100

H -5.36955200 0.59629500 -0.88100500

H -5.27670600 2.98388500 -1.41634800

H -3.43388400 3.87329900 -2.80214200

H -1.71824800 2.33707700 -3.68285600

H -1.85201300 -0.07471500 -3.19014000

H -1.04263200 -0.46686200 -0.97769600

H 3.42369800 3.18792600 -0.99161900

H 3.78996900 5.54104800 -1.61197700

H 1.99695700 7.20390600 -1.37236100

H -0.22133600 6.52027100 -0.52908500

H -2.33098500 5.26293500 0.39492500

H -4.97790500 5.40938000 1.14095000

H -7.26251800 4.94596900 1.94319400

H -7.88560700 2.68774300 2.68170300

H -6.26126600 0.84094400 2.62096000

H -1.85095700 0.07470800 3.18999800

H -1.71696700 -2.33707800 3.68267800

H -3.43285800 -3.87334700 2.80254100

H -5.27616600 -2.98398300 1.41736000

H -5.36925200 -0.59639800 0.88204000

H -1.04231700 0.46687100 0.97727200

H 5.14179000 2.13601100 0.24289200

H 7.55285300 2.16314100 0.24668900

H 9.46027400 2.15550500 0.15096600

H 11.86804900 2.13340400 0.15313300

PIC-TRZ_T1_LC-PBE0*_TDA

C -12.06418500 0.03321100 -0.00411600

C -11.37144500 -1.00306900 -0.60813100

C -9.98819200 -1.00867000 -0.60547400

C -9.27515000 0.02252900 -0.00135100

C -7.80441100 0.01692500 0.00015400

C -7.09780900 -1.17056000 0.17690900

C -5.71793800 -1.17887500 0.17482000

C -5.01267900 0.00636400 0.00330100

C -3.54570500 0.00117800 0.00462100

N -2.94012300 -1.17990800 -0.04254100

C -1.61513300 -1.12309400 -0.02568400

N -0.91794600 -2.30226800 -0.02711600

C -1.42060200 -3.56462200 -0.38787400

C -2.67382100 -3.93275900 -0.85054700

C -2.87431000 -5.26484200 -1.17243600

C -1.86221300 -6.20852300 -1.03982400

C -0.61239700 -5.83133800 -0.58656300

C -0.38858000 -4.50284300 -0.26308400

C 0.78312900 -3.80566000 0.20803400

C 2.05206200 -4.24387700 0.53780100

C 2.96094100 -3.31323700 1.01358700

C 4.34289800 -3.38222400 1.40866300

C 5.26990300 -4.41397200 1.46533600

C 6.55236900 -4.14093000 1.90028300

C 6.90702800 -2.85070800 2.29290300

C 6.00253900 -1.80636000 2.24790000

C 4.72217100 -2.08253000 1.78890100

N 3.63950100 -1.23547300 1.64647400

C 3.61406300 0.14698600 1.90701500

C 4.62588200 0.97477700 1.43747900

C 4.56466900 2.33831600 1.66209200

C 3.48655000 2.88805400 2.33614900

C 2.48541600 2.06085500 2.81619800

C 2.55475600 0.69289000 2.61991000

C 2.57090400 -1.96760900 1.16521600

C 1.31026000 -1.50236300 0.83079100

C 0.43115000 -2.44814100 0.34130300

N -0.90176300 -0.00746500 -0.00137700

C -1.60830300 1.11152500 0.03242900

N -0.90416300 2.28806500 0.04111800

C -1.40473000 3.54627200 0.38952100

C -2.64157300 3.93764300 0.83746000

C -2.82154300 5.29047700 1.14649500

C -1.78631200 6.22543600 1.00509700

C -0.54598900 5.84364700 0.56547100

C -0.32865700 4.47972900 0.25400600

C 0.79875800 3.79706400 -0.18264400

C 2.13520700 4.26605700 -0.50043400

C 3.02222000 3.31642600 -1.00760100

C 4.39919900 3.36124600 -1.40617900

C 5.34858100 4.37615700 -1.47031200

C 6.62105400 4.07299800 -1.91358700

C 6.95679400 2.77699500 -2.31105200

C 6.03154600 1.75011900 -2.25936400

C 4.76563100 2.05466900 -1.78736700

N 3.65972000 1.23572100 -1.63232700

C 3.60678000 -0.14444700 -1.90285900

C 2.53528500 -0.66398200 -2.61712600

C 2.44268100 -2.02944800 -2.82050100

C 3.43176800 -2.87467200 -2.34757600

C 4.52076200 -2.34934500 -1.67054200

C 4.60631100 -0.98923700 -1.43743500

C 2.62191100 1.98758000 -1.15739000

C 1.32437100 1.48851400 -0.79729000

C 0.45069000 2.42911900 -0.31982000

N -2.93224200 1.17806900 0.05381000

C -5.70854300 1.19693700 -0.16969100

C -7.08841600 1.19901500 -0.17495200

C -9.98146200 1.05916900 0.60136800

C -11.36471800 1.06415700 0.60127500

H -13.15034800 0.03738500 -0.00520100

H -11.91298200 -1.81056100 -1.09268100

H -9.44803500 -1.80973500 -1.10400400

H -7.64347300 -2.09475600 0.34808000

H -5.16404100 -2.10008900 0.33021800

H -3.46399600 -3.20078500 -0.94949600

H -3.85037500 -5.57431500 -1.53491600

H -2.05546400 -7.24592400 -1.29631200

H 0.18543200 -6.56260900 -0.48678100

H 2.32430700 -5.29111300 0.43078200

H 4.98838800 -5.42332800 1.17570900

H 7.28886800 -4.93735300 1.94889800

H 7.91567900 -2.65985900 2.64822500

H 6.28044700 -0.80946000 2.57565800

H 5.45514800 0.55533000 0.87641600

H 5.35215500 2.97700700 1.27193900

H 3.42409900 3.96204100 2.48136600

H 1.64263700 2.48223100 3.35645200

H 1.78788800 0.03697900 3.02119300

H 1.03502500 -0.46422400 0.94209300

H -3.44724100 3.22370400 0.94172600

H -3.79252500 5.62080500 1.50209700

H -1.97619200 7.26662100 1.25012000

H 0.26012900 6.56265900 0.45532300

H 2.40755600 5.30519100 -0.35730100

H 5.08999200 5.39190300 -1.18329000

H 7.37183700 4.85620100 -1.96743900

H 7.95949000 2.57105700 -2.67335300

H 6.28522400 0.74744800 -2.58959600

H 1.78268900 0.01004800 -3.01546200

H 1.59302900 -2.43379300 -3.36267000

H 3.35296000 -3.94598800 -2.50520700

H 5.29753600 -3.00414400 -1.28607800

H 5.43896800 -0.58601500 -0.87033200

H 1.06664900 0.44649900 -0.89993500

H -5.14710700 2.11388900 -0.32326600

H -7.62661500 2.12739600 -0.34705800

H -9.43618500 1.85608900 1.10095400

H -11.90099200 1.87579800 1.08473500

PPZ-3TPT_T1_PBE0_TDA

C -9.63906300 -0.28397000 -0.10499400

C -8.75852000 0.74352500 0.20824000

C -7.39159300 0.59619800 0.00970300

C -6.86790200 -0.59962600 -0.50829300

C -5.46104800 -0.83212900 -0.78923400

N -5.05072300 -1.69169300 -1.70016500

N -3.72343200 -1.65029400 -1.78942300

C -3.25502600 -0.75694300 -0.90556900

C -1.86260100 -0.54746300 -0.69278400

C -0.96220000 -1.15450300 -1.62715500

C 0.39669700 -1.04866900 -1.50051700

C 0.94924700 -0.32993300 -0.41863700

N 2.38127800 -0.23052100 -0.27440900

C 3.05881500 0.81110400 -0.86489200

C 2.37347600 1.78140100 -1.61201700

C 3.05937800 2.82118500 -2.19807900

C 4.44737900 2.92662900 -2.05647600

C 5.14506600 1.98599100 -1.32808500

C 4.46758500 0.91905600 -0.72559900

N 5.14260600 -0.03947800 0.00789300

C 6.56727700 0.06059500 0.15562600

C 7.09410700 0.76848400 1.22853100

C 8.47338100 0.86255100 1.36746900

C 9.31087800 0.25291300 0.44005700

C 8.77270900 -0.45319800 -0.62997800

C 7.39466200 -0.55242000 -0.77690400

C 4.47209800 -1.09634200 0.59594800

C 5.15420500 -2.07169200 1.33330000

C 4.46100000 -3.11735400 1.90608700

C 3.07313700 -3.21071000 1.75413700

C 2.38250500 -2.26126200 1.03541000

C 3.06317900 -1.18604300 0.44341700

C 0.10023000 0.25611200 0.52926100

C -1.26715200 0.15509800 0.40169300

N -4.34890900 -0.19320600 -0.24336400

C -4.34442300 0.81648800 0.73837100

C -4.89798700 0.57496900 1.99763400

C -4.91066200 1.57938600 2.95446800

C -4.36498600 2.82882300 2.67294500

C -3.81018200 3.06778500 1.41839600

C -3.80298000 2.07226500 0.45229000

C -7.77259400 -1.62721400 -0.83367800

C -9.13204100 -1.46966000 -0.63628700

H -10.70613000 -0.16359700 0.05590800

H -9.13679300 1.68113600 0.60637200

H -6.73714000 1.42816300 0.23810900

H -1.39344600 -1.71311300 -2.44933300

H 1.04904900 -1.51720400 -2.23272100

H 1.30115700 1.68340700 -1.71088800

H 2.51434400 3.56091700 -2.77333200

H 4.98297800 3.74792100 -2.51929500

H 6.21908900 2.06507100 -1.21789700

H 6.42580300 1.23791000 1.94301200

H 8.89295800 1.41345300 2.20253300

H 10.38749200 0.32833200 0.55170700

H 9.42582100 -0.92907800 -1.35374800

H 6.95767100 -1.09959200 -1.60583900

H 6.22808400 -1.99816200 1.44854900

H 4.99997000 -3.86726100 2.47419000

H 2.53151200 -4.03535700 2.20326600

H 1.31024000 -2.31861700 0.90796700

H 0.52081300 0.78593600 1.38028400

H -1.88279500 0.59762800 1.17263600

H -5.32011600 -0.40144100 2.20981400

H -5.34641700 1.38280300 3.92943800

H -4.37531100 3.61191300 3.42461100

H -3.38989300 4.04180600 1.18587800

H -3.38069500 2.25087500 -0.53100100

H -7.37408600 -2.54422800 -1.25273200

H -9.80618300 -2.28182800 -0.89404000

PPZ-3TPT_T1_LC-PBE_TDA

C -9.43780700 0.04759100 0.05042300

C -8.49778200 1.05054300 -0.01649700

C -7.17376200 0.74732800 -0.23869600

C -6.78181900 -0.56893800 -0.39150200

C -5.40319900 -0.96451600 -0.66402900

N -5.07754400 -1.99922300 -1.36749200

N -3.74015500 -2.07257400 -1.41355800

C -3.25656200 -1.08527900 -0.73462500

C -1.82622500 -0.84595400 -0.56572100

C -0.98790800 -1.27747100 -1.57793700

C 0.37121600 -1.11395700 -1.47061700

C 0.89697400 -0.51986300 -0.34661200

N 2.29933600 -0.34339100 -0.23369500

C 2.85035500 0.81923500 -0.69307200

C 2.08983400 1.83453100 -1.27745300

C 2.66931900 2.97860800 -1.72257500

C 4.07307900 3.16058700 -1.59843600

C 4.83769600 2.19004400 -1.03662300

C 4.26993700 1.00164300 -0.57062400

N 5.01016900 0.00376100 -0.00329600

C 6.41302200 0.18202600 0.11398100

C 6.93038600 0.76159000 1.24852200

C 8.29166100 0.93339800 1.36007700

C 9.12360600 0.52618700 0.34031400

C 8.59668500 -0.05357500 -0.79289200

C 7.23603800 -0.22736700 -0.90866800

C 4.45857500 -1.15940200 0.45399700

C 5.22056700 -2.17717900 1.03319900

C 4.64060900 -3.32081900 1.47818800

C 3.23744100 -3.50046700 1.35950500

C 2.47182600 -2.52873600 0.80075800

C 3.04047000 -1.34127400 0.33418400

C 0.07659900 -0.10087700 0.67277800

C -1.28492000 -0.26526400 0.56585400

N -4.27562400 -0.33809600 -0.22991600

C -4.18228600 0.82659800 0.56629200

C -4.65265100 0.80345700 1.85916600

C -4.55934600 1.93893700 2.63036200

C -3.99077300 3.08191600 2.11242000

C -3.51961800 3.09366900 0.81789800

C -3.61921200 1.96527100 0.03720000

C -7.73185100 -1.57225100 -0.33811900

C -9.05228900 -1.26475400 -0.11651900

H -10.48097700 0.29076400 0.22650200

H -8.79809000 2.08714300 0.09836700

H -6.44764800 1.55014900 -0.30779000

H -1.42748200 -1.75556900 -2.44656800

H 1.04032100 -1.44477300 -2.25824800

H 1.01848400 1.69652400 -1.37499600

H 2.05904500 3.75200400 -2.17535800

H 4.53656500 4.07404000 -1.95375300

H 5.90918600 2.33048100 -0.94448700

H 6.25374900 1.07348800 2.03769500

H 8.70697200 1.38983200 2.25274500

H 10.19669900 0.66223300 0.42995000

H 9.25218400 -0.37451400 -1.59598900

H 6.79623800 -0.68153200 -1.79083900

H 6.29287300 -2.04264000 1.12433600

H 5.25231100 -4.09732200 1.92359500

H 2.77406800 -4.41466900 1.71296300

H 1.40032700 -2.67010000 0.70963000

H 0.51762600 0.35039300 1.55551800

H -1.92263600 0.05182800 1.38345800

H -5.09523600 -0.10716700 2.24988900

H -4.93244300 1.92887200 3.64914200

H -3.91375200 3.97435300 2.72497800

H -3.07071800 3.99281600 0.40897900

H -3.25736000 1.95585200 -0.98599400

H -7.41062700 -2.59767700 -0.48686900

H -9.79026800 -2.05943000 -0.07456600

PPZ-3TPT_T1_LC-PBE0*_TDA

C -9.49895700 0.02203400 0.02491800

C -8.54809800 1.02674700 0.10658800

C -7.20984400 0.74561700 -0.11671200

C -6.81127300 -0.55504000 -0.42013500

C -5.43066600 -0.93090400 -0.70141800

N -5.09452300 -1.94381200 -1.45918300

N -3.75118000 -2.01070200 -1.50578600

C -3.26522600 -1.04028700 -0.77551600

C -1.83705600 -0.80822500 -0.59411800

C -0.98684800 -1.28670600 -1.59205800

C 0.38280600 -1.13375400 -1.47774800

C 0.90691500 -0.50054800 -0.36245200

N 2.31412600 -0.33284600 -0.24427000

C 2.88125700 0.81186200 -0.74504200

C 2.12990300 1.81435000 -1.37465300

C 2.72981600 2.94517100 -1.86217900

C 4.13886400 3.11871000 -1.73431100

C 4.89743700 2.15455600 -1.12561900

C 4.31021500 0.98626100 -0.61821200

N 5.03887200 -0.00168900 -0.00563300

C 6.44691200 0.16774000 0.11456500

C 6.96331700 0.78921800 1.23880100

C 8.33614700 0.95270300 1.35290700

C 9.17513800 0.49579100 0.34710800

C 8.64708500 -0.12546500 -0.77525000

C 7.27516500 -0.29191600 -0.89514800

C 4.47153600 -1.14650200 0.49344700

C 5.22412700 -2.15191600 1.11840800

C 4.62356200 -3.28201100 1.60572200

C 3.21467400 -3.45313400 1.48288200

C 2.45521900 -2.48771800 0.87698300

C 3.04305300 -1.32021800 0.36911500

C 0.07932500 -0.03274000 0.64241300

C -1.29333000 -0.18833200 0.53040700

N -4.29696100 -0.31687100 -0.23684100

C -4.20952200 0.81395500 0.61171900

C -4.64889300 0.71898700 1.92320700

C -4.56073100 1.82752100 2.74934200

C -4.02908900 3.01508300 2.26785700

C -3.58915300 3.09857600 0.95474900

C -3.68259100 1.99790700 0.11878100

C -7.77459700 -1.55959100 -0.51435400

C -9.10818400 -1.27120700 -0.29259700

H -10.54621100 0.24771200 0.20177600

H -8.84886300 2.04383900 0.33855900

H -6.48182800 1.54889800 -0.07000900

H -1.42612700 -1.78710100 -2.44843400

H 1.05541200 -1.49771600 -2.24787600

H 1.05730500 1.67869000 -1.47269300

H 2.12901200 3.70620900 -2.34800800

H 4.61509300 4.01342000 -2.12002200

H 5.97086000 2.28392600 -1.02884500

H 6.28375800 1.13685400 2.01061600

H 8.75102800 1.43841000 2.23047600

H 10.24927600 0.62474100 0.43870200

H 9.30497700 -0.48258500 -1.56123100

H 6.83510200 -0.77448600 -1.76221600

H 6.29763200 -2.02008100 1.21042300

H 5.22548500 -4.04659700 2.08448000

H 2.73867900 -4.34875200 1.86662800

H 1.38183200 -2.61782800 0.78069400

H 0.51883800 0.44514200 1.51208800

H -1.93305000 0.16189400 1.33319500

H -5.06059800 -0.21971600 2.28054700

H -4.90664000 1.76129600 3.77585800

H -3.95695800 3.88015400 2.91943300

H -3.17202400 4.02652200 0.57705200

H -3.34613100 2.04275800 -0.91238500

H -7.45012500 -2.56185800 -0.77440900

H -9.84856600 -2.06178000 -0.36679000

PPZ-DPO_T1_PBE0_TDA

C -9.68207600 -0.00015400 1.76820400

C -8.44470300 -0.00028700 2.40308600

C -7.27120600 -0.00022700 1.66332200

C -7.31724800 -0.00002800 0.26109100

C -6.11533000 0.00005100 -0.52864700

N -5.99977800 0.00026800 -1.82767800

N -4.68644800 0.00019900 -2.13016000

C -4.02424900 0.00009700 -0.97984400

C -2.63256000 0.00004700 -0.76754500

C -2.05767500 -0.00007200 0.53870700

C -0.69443700 -0.00010000 0.71260300

C 0.15869100 -0.00001300 -0.40252500

N 1.59059400 -0.00001300 -0.21763400

C 2.26878200 1.19406700 -0.13312100

C 1.58658000 2.41703300 -0.22495500

C 2.27474300 3.60624100 -0.13830400

C 3.66214400 3.61368700 0.04284800

C 4.35684400 2.42599800 0.13561100

C 3.67697600 1.20506700 0.04906400

N 4.34944400 -0.00000800 0.13740600

C 5.77372100 -0.00000400 0.32034200

C 6.29639500 -0.00009400 1.60745100

C 7.67513200 -0.00008800 1.77947900

C 8.51608200 0.00000800 0.67224700

C 7.98194700 0.00009700 -0.61145800

C 6.60446900 0.00009200 -0.79311300

C 3.67700400 -1.20508500 0.04888300

C 4.35690100 -2.42601300 0.13525000

C 3.66222700 -3.61370300 0.04231400

C 2.27482500 -3.60626300 -0.13883500

C 1.58663600 -2.41705900 -0.22531200

C 2.26880900 -1.19409000 -0.13330100

C -0.38164400 0.00010000 -1.70383700

C -1.74030000 0.00012800 -1.88319800

O -4.89305600 -0.00001100 0.08535000

C -8.57038800 0.00010900 -0.37429000

C -9.73331700 0.00004300 0.37366700

H -10.59813800 -0.00020200 2.35085300

H -8.39207400 -0.00044000 3.48834400

H -6.30855100 -0.00033100 2.16313800

H -2.71041400 -0.00013600 1.40433500

H -0.27524600 -0.00018700 1.71538200

H 0.51475700 2.39481100 -0.36525800

H 1.73133200 4.54133900 -0.21212100

H 4.19926700 4.55305600 0.11084900

H 5.43033000 2.42893100 0.27526400

H 5.62554200 -0.00016700 2.46035100

H 8.09155700 -0.00015700 2.78119600

H 9.59225800 0.00001300 0.81034600

H 8.63777800 0.00017200 -1.47559400

H 6.17078600 0.00016100 -1.78780500

H 5.43038600 -2.42894100 0.27490200

H 4.19937200 -4.55307100 0.11017900

H 1.73143700 -4.54136300 -0.21278600

H 0.51481200 -2.39484000 -0.36560900

H 0.28064100 0.00016800 -2.56545300

H -2.16508600 0.00021600 -2.88059600

H -8.60137600 0.00026700 -1.45848400

H -10.69427000 0.00015100 -0.13306100

PPZ-DPO_T1_LC-PBE_TDA

C 9.52302500 -0.00003900 1.79061300

C 8.29408200 -0.00011900 2.41108800

C 7.14228200 -0.00011300 1.65902200

C 7.22495200 -0.00002400 0.28193000

C 6.02970000 -0.00000800 -0.53757500

N 5.94152700 0.00013300 -1.81086900

N 4.61673500 0.00001500 -2.11181600

C 3.98827300 0.00003600 -1.00202300

C 2.55546600 0.00002000 -0.78184200

C 2.03361800 -0.00000100 0.49543500

C 0.67049900 -0.00001300 0.67574000

C -0.15910500 0.00000000 -0.42021900

N -1.56380400 -0.00000200 -0.23206200

C -2.21033800 -1.20098400 -0.14450500

C -1.54426700 -2.42433900 -0.23683300

C -2.21892000 -3.59911500 -0.14512700

C -3.62564800 -3.59850300 0.04781200

C -4.29967100 -2.42411700 0.14029600

C -3.63304600 -1.19979400 0.04945700

N -4.27943800 -0.00000200 0.13734700

C -5.68645900 -0.00000200 0.32585800

C -6.19950300 -0.00004000 1.60152300

C -7.56428000 -0.00003800 1.78157100

C -8.40365100 0.00000300 0.68918100

C -7.88074800 0.00004200 -0.58538000

C -6.51665000 0.00003900 -0.77020900

C -3.63303800 1.19979000 0.04951600

C -4.29965400 2.42411300 0.14041400

C -3.62562400 3.59849800 0.04798300

C -2.21889500 3.59910900 -0.14495500

C -1.54424900 2.42433400 -0.23671100

C -2.21033100 1.20097900 -0.14444900

C 0.35611100 0.00002000 -1.69714800

C 1.71635600 0.00002600 -1.87975200

O 4.82332800 -0.00001100 0.04330900

C 8.45719500 0.00006000 -0.34268000

C 9.60290200 0.00004900 0.41396500

H 10.43049500 -0.00004500 2.38614600

H 8.23080500 -0.00018800 3.49435400

H 6.17062800 -0.00017500 2.14172200

H 2.69907100 -0.00000700 1.35197700

H 0.23448400 -0.00002700 1.66921600

H -0.46983900 -2.42973100 -0.38559000

H -1.68079200 -4.53722600 -0.22077500

H -4.16431300 -4.53655500 0.12081200

H -5.37427800 -2.42699500 0.28691400

H -5.51715400 -0.00007100 2.44551400

H -7.97642600 -0.00006800 2.78545000

H -9.47945200 0.00000600 0.83301400

H -8.54210100 0.00007600 -1.44574700

H -6.08000600 0.00007000 -1.76382400

H -5.37426100 2.42699100 0.28703500

H -4.16428200 4.53655000 0.12102900

H -1.68076000 4.53722100 -0.22055400

H -0.46982100 2.42972500 -0.38546600

H -0.32440900 0.00003000 -2.54218100

H 2.15160700 0.00004200 -2.87356100

H 8.49515700 0.00013300 -1.42712700

H 10.57158300 0.00011400 -0.07500800

PPZ-DPO_T1_LC-PBE0*_TDA

C 9.61725400 -0.87206700 1.53917100

C 8.38218400 -1.19876200 2.07832400

C 7.21521400 -0.82187800 1.43429200

C 7.27669100 -0.10991400 0.23701500

C 6.07780800 0.29436500 -0.45292300

N 5.97615300 0.95407400 -1.56054600

N 4.65745700 1.10591100 -1.82954200

C 4.00138600 0.51689500 -0.84744000

C 2.61392100 0.39978500 -0.66405500

C 2.04972700 -0.27409200 0.45722400

C 0.69551500 -0.37233600 0.60627700

C -0.16313700 0.20157700 -0.35548800

N -1.57421500 0.10493200 -0.19654600

C -2.20005500 -1.10919300 -0.35873300

C -1.46756700 -2.24888300 -0.71072900

C -2.10036000 -3.46134800 -0.85787700

C -3.47621000 -3.56767400 -0.66113500

C -4.22148700 -2.45579800 -0.33165100

C -3.59838500 -1.21693600 -0.18192700

N -4.32291800 -0.08343100 0.11519200

C -5.73776400 -0.18032900 0.27579100

C -6.26415600 -0.42381300 1.53416500

C -7.63888500 -0.51683400 1.68590700

C -8.47109600 -0.36688300 0.58606400

C -7.93308000 -0.12343500 -0.66932700

C -6.55943100 -0.02888700 -0.82959700

C -3.71010400 1.14233200 0.25771400

C -4.44557600 2.28834500 0.55989200

C -3.80294100 3.49631500 0.72715000

C -2.41672800 3.58014900 0.60721700

C -1.67476100 2.46137800 0.30713000

C -2.30635500 1.22708300 0.11542300

C 0.37667400 0.87601900 -1.47508300

C 1.72815000 0.97057300 -1.62602100

O 4.86397100 -0.02163700 0.06459500

C 8.52178000 0.21793700 -0.30401400

C 9.68001900 -0.16210200 0.34524100

H 10.53023200 -1.16886900 2.04636800

H 8.32581700 -1.75302800 3.01064500

H 6.24626700 -1.07499100 1.85239900

H 2.71050300 -0.70561900 1.20183300

H 0.27358300 -0.87698800 1.47163100

H -0.40098100 -2.14312300 -0.86438400

H -1.52024100 -4.33493000 -1.13325600

H -3.97155900 -4.52572100 -0.77570200

H -5.29413200 -2.53024400 -0.19428300

H -5.59440500 -0.53750800 2.38093800

H -8.06066100 -0.70713900 2.66728000

H -9.54688500 -0.44023000 0.70817800

H -8.58477200 -0.00625000 -1.52883900

H -6.11693200 0.16096900 -1.80257000

H -5.52165900 2.21516200 0.66785700

H -4.38496600 4.38095400 0.96169200

H -1.91348400 4.52925200 0.75366600

H -0.59686500 2.50429500 0.21368200

H -0.29048600 1.30307700 -2.21938000

H 2.16069900 1.47496500 -2.48366900

H 8.55006200 0.77237400 -1.23662600

H 10.64427800 0.09675200 -0.08209000

PXZ-OXD_T1_PBE0_TDA

C -8.38147700 -0.00092700 -1.53969100

C -7.17021200 -0.00098300 -2.22251500

C -5.96900300 -0.00044000 -1.52816600

C -5.96211200 0.00016800 -0.12624000

C -4.72935400 0.00065000 0.61799600

N -4.56501300 0.00126500 1.91005900

N -3.23911000 0.00143600 2.16218000

C -2.62343900 0.00092900 0.98715900

C -1.24133800 0.00076800 0.72028100

C -0.71741200 0.00025100 -0.60799900

C 0.63662200 0.00019800 -0.83571500

C 1.53099000 0.00083600 0.24925700

N 2.95357600 0.00016400 0.00949200

C 3.64099500 1.18483700 -0.10385100

C 2.99999200 2.43002700 0.00861200

C 3.73104700 3.58955500 -0.11252000

C 5.11321500 3.55073800 -0.34838500

C 5.76502400 2.33858200 -0.46241300

C 5.03360400 1.16605500 -0.34027500

O 5.70663500 -0.00104900 -0.45682900

C 5.03266000 -1.16756500 -0.33972600

C 5.76314500 -2.34073000 -0.46130800

C 5.11035300 -3.55230700 -0.34672600

C 3.72815900 -3.58989800 -0.11082400

C 2.99803500 -2.42972400 0.00979600

C 3.64004300 -1.18511800 -0.10329100

C 1.04299400 0.00114700 1.57289100

C -0.30708900 0.00118700 1.80216800

O -3.53276800 0.00041500 -0.04256200

C -7.18828000 0.00023000 0.55775100

C -8.37966600 -0.00031000 -0.14470700

H -9.31934800 -0.00136700 -2.08653800

H -7.15983300 -0.00146400 -3.30887400

H -5.02627300 -0.00050500 -2.06459300

H -1.40300400 -0.00002800 -1.44780400

H 1.01662500 -0.00019100 -1.85387400

H 1.93391500 2.44329600 0.19094100

H 3.22744800 4.54519800 -0.02328400

H 5.67462800 4.47330700 -0.44152700

H 6.83129700 2.27018500 -0.64395100

H 6.82947500 -2.27326900 -0.64286000

H 5.67102300 -4.47537300 -0.43940400

H 3.22378000 -4.54509100 -0.02117800

H 1.93196600 -2.44208700 0.19222600

H 1.73738300 0.00148700 2.40873100

H -0.69379800 0.00155400 2.81489200

H -7.17698900 0.00069200 1.64230700

H -9.32038100 -0.00026600 0.39844100

PXZ-OXD_T1_LC-PBE_TDA

C 8.25088600 0.00027500 -1.55439800

C 7.04866800 0.00047000 -2.22524800

C 5.86680500 0.00034400 -1.52146200

C 5.89265300 0.00002100 -0.14210900

C 4.66482900 -0.00011800 0.62751400

N 4.52433000 -0.00047400 1.89630200

N 3.18853800 -0.00033500 2.14257800

C 2.60644400 -0.00024500 1.00806600

C 1.18341100 -0.00018700 0.73095800

C 0.71271200 -0.00003700 -0.56595000

C -0.64215600 0.00002300 -0.80032600

C -1.51293800 -0.00006700 0.26306400

N -2.90956600 0.00000200 0.01949200

C -3.56430500 -1.19054600 -0.09637600

C -2.94149300 -2.43904500 0.01620700

C -3.65902500 -3.58337800 -0.11098800

C -5.05911900 -3.53937700 -0.36141600

C -5.68790900 -2.33601900 -0.47439900

C -4.97307900 -1.16122200 -0.34742600

O -5.62228700 0.00013900 -0.46375400

C -4.97298200 1.16143400 -0.34731700

C -5.68770400 2.33630300 -0.47422800

C -5.05881200 3.53959700 -0.36113700

C -3.65872100 3.58345700 -0.11066800

C -2.94129100 2.43905100 0.01645500

C -3.56420700 1.19061600 -0.09624800

C -1.05003500 -0.00021800 1.55976800

C 0.30202900 -0.00027700 1.79520100

O 3.48302300 -0.00003200 -0.00230000

C 7.09796700 -0.00018100 0.53302900

C 8.27393200 -0.00004900 -0.17565100

H 9.18216800 0.00037500 -2.11193200

H 7.03031600 0.00072400 -3.31018600

H 4.91605600 0.00049800 -2.04414200

H 1.41146800 0.00003300 -1.39548500

H -1.03801400 0.00014200 -1.81052100

H -1.87467900 -2.48288400 0.20839600

H -3.15915900 -4.54114500 -0.01954500

H -5.62226000 -4.45995700 -0.46084300

H -6.75374100 -2.26308300 -0.66434700

H -6.75353800 2.26347500 -0.66420900

H -5.62186800 4.46023400 -0.46051700

H -3.15877400 4.54117400 -0.01913700

H -1.87447700 2.48278300 0.20866700

H -1.76274800 -0.00028800 2.37789400

H 0.69800600 -0.00039200 2.80528200

H 7.09129800 -0.00043800 1.61811700

H 9.22159100 -0.00020500 0.35286700

PXZ-OXD_T1_LC-PBE0*_TDA

C -8.35857800 0.92492000 -1.11856400

C -7.16537800 1.42227100 -1.62343700

C -5.95541300 0.97320600 -1.12499600

C -5.93325400 0.01502400 -0.10930600

C -4.69317000 -0.46817100 0.42509400

N -4.51856900 -1.35531000 1.36742300

N -3.20404000 -1.50550800 1.55222800

C -2.59636200 -0.68394600 0.69254400

C -1.23247200 -0.48937400 0.51074100

C -0.71624000 0.43412700 -0.45644800

C 0.62434100 0.60615200 -0.59968800

C 1.53619800 -0.14464500 0.19054400

N 2.91471200 0.02462800 0.02331600

C 3.49947700 1.27890600 0.15860200

C 2.76519600 2.40104800 0.54989400

C 3.37693100 3.63495700 0.65703000

C 4.73453300 3.77330700 0.39981000

C 5.48613500 2.66209700 0.05932300

C 4.87694500 1.42767400 -0.05024100

O 5.67894200 0.37100200 -0.32824700

C 5.11539600 -0.85834500 -0.42436500

C 5.96129100 -1.91103800 -0.71331300

C 5.44383500 -3.18296900 -0.88535000

C 4.07338400 -3.38773900 -0.79371400

C 3.22475500 -2.33626500 -0.50667200

C 3.73578100 -1.05595500 -0.28187100

C 1.03569800 -1.07148600 1.14606700

C -0.30151500 -1.23314400 1.31258500

O -3.51643100 0.00280300 -0.05044800

C -7.13697300 -0.48477800 0.39804300

C -8.33805300 -0.02938700 -0.10684400

H -9.30586300 1.27987900 -1.51242100

H -7.17831300 2.16712400 -2.41325000

H -5.01896900 1.35790500 -1.51550100

H -1.40992500 0.98106300 -1.08574200

H 1.01376500 1.28379500 -1.35431500

H 1.71341600 2.28326700 0.78231700

H 2.78828400 4.49364900 0.96080400

H 5.21469400 4.74187900 0.48647800

H 6.55526300 2.72115200 -0.11321600

H 7.02096200 -1.70229800 -0.81239500

H 6.10926800 -4.00873500 -1.11227300

H 3.65595500 -4.37446600 -0.96170000

H 2.15265100 -2.48767500 -0.46515200

H 1.73680500 -1.61361600 1.77432500

H -0.69845900 -1.91011700 2.06107600

H -7.09913200 -1.22868000 1.18719500

H -9.27014000 -0.41993100 0.28976300

PXZ-TAZ_T1_PBE0_TDA

C 8.34830600 -0.46424100 -0.13239900

C 7.81386100 -1.71219600 -0.44895500

C 6.44815900 -1.87549600 -0.58824900

C 5.56940300 -0.79019200 -0.41369000

C 4.15346400 -1.04582300 -0.60952000

N 3.70736800 -2.10208100 -1.27324100

N 2.38677300 -2.06538400 -1.33241800

C 1.94754200 -0.96956900 -0.67561500

C 0.57413000 -0.69261200 -0.49945200

C 0.01271400 0.33752300 0.33354300

C -1.34001200 0.50643900 0.43734700

C -2.23606900 -0.34318200 -0.25849700

N -3.63513000 -0.17192800 -0.12587500

C -4.24810200 1.00820600 -0.53020100

C -3.53385100 2.05082400 -1.13774100

C -4.17909100 3.21467700 -1.51313700

C -5.55008000 3.36533400 -1.31016200

C -6.28008700 2.33125900 -0.74545100

C -5.63697700 1.16452200 -0.36693500

O -6.41329700 0.16933300 0.13521700

C -5.81975800 -0.99480600 0.50827400

C -6.64393300 -1.98082400 1.02472900

C -6.09292500 -3.17242700 1.46877600

C -4.71250700 -3.36017300 1.41320400

C -3.88659600 -2.37729100 0.90000200

C -4.42850900 -1.18039300 0.41036200

C -1.70725900 -1.38761200 -1.06526400

C -0.36108000 -1.55271700 -1.18585300

N 3.06921000 -0.28088600 -0.20841500

C 3.09968200 0.92770000 0.52795400

C 2.75589900 2.12724300 -0.09264300

C 2.79234300 3.31236700 0.62956300

C 3.17977500 3.30640100 1.96598000

C 3.52730300 2.10784600 2.58029400

C 3.48429200 0.91756100 1.86646600

C 6.11956500 0.46482900 -0.10798300

C 7.49248100 0.61785200 0.03045000

H 9.42068500 -0.33803700 -0.01958300

H 8.47044900 -2.56646800 -0.58537700

H 6.02638900 -2.84128200 -0.84135400

H 0.65512000 0.97530300 0.92502300

H -1.73672600 1.26955100 1.10116300

H -2.47424400 1.92178500 -1.31504100

H -3.60828600 4.00882700 -1.98170400

H -6.05245900 4.27890700 -1.60832900

H -7.35225200 2.39777500 -0.59864900

H -7.70889500 -1.78454900 1.07809100

H -6.73655700 -3.94576800 1.87320600

H -4.27290100 -4.27935600 1.78438900

H -2.81345200 -2.51373300 0.87521500

H -2.38457300 -2.03192800 -1.61855800

H 0.04407300 -2.33328900 -1.81771800

H 2.46250800 2.11625100 -1.13720200

H 2.52475800 4.24579400 0.14414800

H 3.21263700 4.23496200 2.52716300

H 3.83048400 2.09784200 3.62253200

H 3.75077800 -0.02461400 2.33349900

H 5.48347600 1.33365100 0.00336300

H 7.89530200 1.59949700 0.26245700

PXZ-TAZ_T1_LC-PBE_TDA

C -8.24744700 0.33963900 0.17748600

C -7.75474500 1.08361400 1.22890700

C -6.40355100 1.28013800 1.36103700

C -5.51957500 0.73090700 0.44429700

C -4.10699900 1.01017300 0.64920700

N -3.68793500 2.01302200 1.37437500

N -2.37514900 2.01935700 1.38666700

C -1.93303700 0.98583800 0.66083600

C -0.57794700 0.69466100 0.52349600

C -0.01981500 -0.47524600 -0.10841800

C 1.30878000 -0.63852100 -0.19352300

C 2.21370100 0.34481700 0.31994200

N 3.57810100 0.16687900 0.18342800

C 4.38490300 1.13786300 -0.41645400

C 3.87886000 2.32057700 -0.91595800

C 4.70654100 3.25427100 -1.49999200

C 6.05327500 3.01185500 -1.61376100

C 6.56307500 1.81587000 -1.16024800

C 5.73895400 0.88799700 -0.58137000

O 6.30548300 -0.29059700 -0.22303700

C 5.54064000 -1.20350600 0.42174000

C 6.16591600 -2.33932700 0.86157900

C 5.46183900 -3.29206100 1.56460600

C 4.13467900 -3.07828200 1.84537600

C 3.50845100 -1.93242400 1.40621500

C 4.19026300 -0.99547900 0.65600400

C 1.67761400 1.47999200 0.98839400

C 0.35230700 1.64483900 1.10642700

N -3.03785700 0.32300600 0.16775600

C -3.05790000 -0.81728800 -0.66455500

C -2.74481400 -0.68651300 -1.99943400

C -2.76628000 -1.79685500 -2.81069800

C -3.10705300 -3.02644200 -2.28881500

C -3.42322500 -3.14704900 -0.95370500

C -3.39618600 -2.04082500 -0.13512300

C -6.02042600 -0.00515300 -0.61572800

C -7.37747900 -0.19700700 -0.74321200

H -9.31650500 0.18320100 0.07304800

H -8.43408100 1.51694100 1.95597900

H -5.99912000 1.87250900 2.17445300

H -0.66878500 -1.23496600 -0.52375400

H 1.72242200 -1.51067800 -0.69008200

H 2.81306600 2.50739300 -0.86193700

H 4.27998200 4.17718000 -1.87803300

H 6.71125800 3.74236800 -2.07168600

H 7.61377500 1.56543000 -1.25879200

H 7.22315200 -2.44992600 0.64611700

H 5.96280400 -4.19038200 1.90870600

H 3.57255500 -3.80063600 2.42749500

H 2.47126000 -1.74998200 1.66328400

H 2.36223500 2.18708600 1.44633800

H -0.06795800 2.48185900 1.65041300

H -2.48004200 0.29146500 -2.38879700

H -2.51606900 -1.70103900 -3.86216800

H -3.12583200 -3.90096700 -2.93116800

H -3.69195800 -4.11459900 -0.54265100

H -3.63983300 -2.11416300 0.91972600

H -5.35792400 -0.42354000 -1.36385200

H -7.75829400 -0.77206300 -1.58124200

PXZ-TAZ_T1_LC-PBE0*_TDA

C 8.24687600 -0.16323000 -0.08386300

C 7.81096200 -1.42469600 -0.46395700

C 6.46569200 -1.65994600 -0.67730500

C 5.53607100 -0.63325400 -0.51153000

C 4.14012400 -0.95167900 -0.78767900

N 3.76027300 -1.91330100 -1.59057200

N 2.41538400 -1.93745900 -1.61589800

C 1.97191800 -0.99263700 -0.82754900

C 0.55416300 -0.73159800 -0.60825900

C 0.04721600 -0.14535200 0.55085200

C -1.31855300 0.03878200 0.69642300

C -2.17460000 -0.36804100 -0.31083900

N -3.57512200 -0.17272500 -0.15729500

C -4.13606800 0.99962300 -0.58706800

C -3.40936400 2.03772200 -1.19241600

C -4.03762600 3.18382800 -1.59744600

C -5.44624100 3.34650900 -1.41223900

C -6.17799100 2.34457000 -0.82477600

C -5.55605500 1.17501600 -0.40875500

O -6.30434500 0.21639700 0.15900800

C -5.74171200 -0.92939100 0.57279100

C -6.55287700 -1.89500700 1.15400500

C -6.01357600 -3.07918500 1.59127400

C -4.61232800 -3.31862900 1.44785600

C -3.79888600 -2.37771000 0.87771500

C -4.32727800 -1.15847700 0.42238400

C -1.68895100 -0.96703300 -1.46172200

C -0.32605500 -1.14760500 -1.60827600

N 3.03391700 -0.32942200 -0.27122100

C 2.99553700 0.75760600 0.63641000

C 2.50588400 1.98341300 0.21233400

C 2.46010600 3.04106000 1.10579000

C 2.91006700 2.87300700 2.40732200

C 3.40431100 1.64399100 2.81954300

C 3.44493200 0.57821800 1.93551100

C 5.97966600 0.63613800 -0.14509000

C 7.32943000 0.86389600 0.06914300

H 9.30338900 0.02060400 0.08624400

H 8.52503500 -2.23205600 -0.59406900

H 6.10601600 -2.63625200 -0.98545500

H 0.71000300 0.15622000 1.35459000

H -1.72933300 0.49083800 1.59354800

H -2.34013900 1.91400800 -1.33573200

H -3.46082300 3.97388100 -2.06564100

H -5.93578500 4.25768600 -1.73674600

H -7.24904500 2.43330300 -0.67051100

H -7.61297700 -1.67977600 1.24678800

H -6.65008100 -3.83127600 2.04346700

H -4.18715000 -4.25500900 1.79189000

H -2.73455100 -2.56245800 0.76800600

H -2.38417400 -1.28293200 -2.23301200

H 0.08489200 -1.62278900 -2.49261200

H 2.16168800 2.09443900 -0.81130900

H 2.07304400 4.00192000 0.78220300

H 2.87547100 3.70452200 3.10408900

H 3.75835300 1.51189000 3.83688100

H 3.82728900 -0.39167000 2.23802900

H 5.27808200 1.45748400 -0.04185000

H 7.66562500 1.85714600 0.35051200

PXZ-TRZ_T1_PBE0_TDA

C 5.92176500 -4.82184200 -0.00153700

C 6.60189300 -3.60964600 -0.00110500

C 5.89763100 -2.41254400 -0.00073300

C 4.50172900 -2.41181700 -0.00078900

C 3.75191900 -1.13073900 -0.00040200

N 4.48443300 0.00000000 0.00001500

C 3.75191900 1.13074000 0.00043500

C 4.50172900 2.41181800 0.00079800

C 5.89763100 2.41254400 0.00074700

C 6.60189300 3.60964600 0.00110600

C 5.92176600 4.82184200 0.00152000

C 4.52965800 4.82892800 0.00157000

C 3.82580500 3.63388200 0.00120900

N 2.43710900 1.19549600 0.00043400

C 1.78544600 0.00000100 -0.00001100

C 0.35485800 0.00000100 -0.00000800

C -0.38445400 1.21444600 0.00049200

C -1.75968000 1.21707900 0.00050600

C -2.45602900 0.00000000 -0.00000200

N -3.90047800 0.00000000 0.00000000

C -4.59427100 -0.00086500 1.18593300

C -3.94263800 -0.00173600 2.43064300

C -4.68358900 -0.00259200 3.59020400

C -6.08576900 -0.00261300 3.55153400

C -6.74788100 -0.00174600 2.33971900

C -6.00668100 -0.00086900 1.16700900

O -6.68964800 0.00001400 0.00000300

C -6.00668300 0.00088000 -1.16700300

C -6.74788600 0.00175400 -2.33971300

C -6.08577800 0.00260700 -3.55152800

C -4.68359800 0.00257500 -3.59020200

C -3.94264400 0.00172200 -2.43064300

C -4.59427400 0.00086400 -1.18593100

C -1.75967900 -1.21707900 -0.00051200

C -0.38445300 -1.21444500 -0.00050600

N 2.43710900 -1.19549500 -0.00043700

C 3.82580400 -3.63388100 -0.00122000

C 4.52965700 -4.82892700 -0.00159400

H 6.47291700 -5.75802500 -0.00183000

H 7.68818100 -3.59612400 -0.00105800

H 6.41178200 -1.45862800 -0.00039800

H 6.41178200 1.45862800 0.00042700

H 7.68818100 3.59612300 0.00106500

H 6.47291900 5.75802500 0.00180400

H 3.99166100 5.77284800 0.00189400

H 2.74180900 3.62161700 0.00124600

H 0.16589700 2.14745900 0.00088200

H -2.30561900 2.15685000 0.00092400

H -2.86109100 -0.00171200 2.44617900

H -4.17194600 -0.00325500 4.54565000

H -6.65447600 -0.00330400 4.47425800

H -7.82946500 -0.00171000 2.27163900

H -7.82947000 0.00172700 -2.27163000

H -6.65448700 0.00329400 -4.47425100

H -4.17195700 0.00322800 -4.54565000

H -2.86109700 0.00168900 -2.44618200

H -2.30561800 -2.15685000 -0.00092900

H 0.16589800 -2.14745800 -0.00090200

H 2.74180800 -3.62161500 -0.00125900

H 3.99166000 -5.77284700 -0.00193100

PXZ-TRZ_T1_LC-PBE_TDA

C -5.83598400 -4.77645300 0.00743600

C -6.52452100 -3.58294300 0.00563700

C -5.83689600 -2.39295000 0.00378200

C -4.45602300 -2.39176000 0.00372200

C -3.72053000 -1.11923700 0.00173800

N -4.41725800 0.00000500 -0.00000400

C -3.72052300 1.11924300 -0.00174700

C -4.45601000 2.39177000 -0.00372500

C -5.83688300 2.39296700 -0.00378200

C -6.52450200 3.58296400 -0.00563100

C -5.83595900 4.77647000 -0.00742800

C -4.45811300 4.77885800 -0.00737300

C -3.76909800 3.58964600 -0.00552800

N -2.40133000 1.16472000 -0.00182700

C -1.78671700 -0.00000300 -0.00000500

C -0.31340600 -0.00000700 -0.00000300

C 0.37752200 1.19517600 -0.00212100

C 1.75150900 1.19746600 -0.00214100

C 2.42823900 -0.00001400 -0.00000100

N 3.84686800 -0.00001700 0.00000000

C 4.51158600 -0.00410100 -1.19007000

C 3.87827000 -0.00812100 -2.43870100

C 4.60668300 -0.01212300 -3.58303600

C 6.02900000 -0.01228500 -3.53949100

C 6.66818800 -0.00825000 -2.33601000

C 5.94263500 -0.00412000 -1.16128700

O 6.60263900 0.00002300 0.00000300

C 5.94263300 0.00413600 1.16129200

C 6.66818300 0.00827700 2.33601500

C 6.02899300 0.01228100 3.53949600

C 4.60667600 0.01209400 3.58303800

C 3.87826500 0.00809300 2.43870100

C 4.51158400 0.00408800 1.19007200

C 1.75150200 -1.19749100 0.00213800

C 0.37751500 -1.19519300 0.00211500

N -2.40133600 -1.16472200 0.00181800

C -3.76911800 -3.58964000 0.00552800

C -4.45813900 -4.77884900 0.00737900

H -6.37977200 -5.71616700 0.00889900

H -7.60977500 -3.58072300 0.00568100

H -6.35915300 -1.44295900 0.00234900

H -6.35914500 1.44297900 -0.00235200

H -7.60975500 3.58075000 -0.00567400

H -6.37974100 5.71618700 -0.00888700

H -3.91542700 5.71867700 -0.00878600

H -2.68522500 3.57001100 -0.00545000

H -0.18345500 2.12258400 -0.00377600

H 2.31420900 2.12527600 -0.00380300

H 2.79423900 -0.00796200 -2.48133600

H 4.09822200 -0.01519100 -4.54067800

H 6.60075700 -0.01553300 -4.46015100

H 7.75085600 -0.00812400 -2.26336100

H 7.75085100 0.00817300 2.26336800

H 6.60074900 0.01552700 4.46015600

H 4.09821300 0.01514600 4.54067900

H 2.79423400 0.00791600 2.48133500

H 2.31419700 -2.12530300 0.00380100

H -0.18346700 -2.12259800 0.00377000

H -2.68524400 -3.57001100 0.00544800

H -3.91545800 -5.71867000 0.00879500

PXZ-TRZ_T1_LC-PBE0*_TDA

C 5.88536300 4.66546700 -1.11156600

C 6.56813500 3.49347000 -0.82242300

C 5.86752600 2.33168900 -0.54625300

C 4.47705500 2.33377200 -0.55686600

C 3.73044400 1.09472600 -0.26189200

N 4.45346100 -0.00000400 -0.00002100

C 3.73044500 -1.09473400 0.26184900

C 4.47705700 -2.33377200 0.55685400

C 5.86752800 -2.33169000 0.54623600

C 6.56813800 -3.49346400 0.82243000

C 5.88536700 -4.66545400 1.11160400

C 4.49751100 -4.67163900 1.12347200

C 3.79631600 -3.51149400 0.84728300

N 2.41570800 -1.15674500 0.27926400

C 1.77155700 -0.00000300 -0.00001200

C 0.35157600 -0.00000200 -0.00000600

C -0.38154900 -1.18985200 0.28596700

C -1.74210900 -1.19559900 0.27548400

C -2.45365300 -0.00000100 -0.00000200

N -3.85674800 0.00000100 0.00000100

C -4.56418200 -0.83870600 -0.84267700

C -3.92184300 -1.66341600 -1.77268500

C -4.66119400 -2.49790700 -2.58191500

C -6.05014000 -2.51693900 -2.49935200

C -6.70318100 -1.67693600 -1.61763000

C -5.96622400 -0.83981200 -0.80301900

O -6.65527600 0.00000900 -0.00001000

C -5.96622600 0.83981900 0.80301100

C -6.70318700 1.67694000 1.61762200

C -6.05015100 2.51693300 2.49935700

C -4.66120600 2.49789300 2.58193400

C -3.92185100 1.66340400 1.77270600

C -4.56418400 0.83870700 0.84268200

C -1.74210800 1.19559700 -0.27549100

C -0.38154900 1.18984900 -0.28597500

N 2.41570700 1.15673900 -0.27928900

C 3.79631300 3.51150100 -0.84726100

C 4.49750700 4.67165300 -1.12342600

H 6.43560800 5.57677400 -1.32852100

H 7.65427500 3.48544400 -0.81241900

H 6.37781100 1.40199900 -0.31729400

H 6.37781200 -1.40200500 0.31725400

H 7.65427800 -3.48543900 0.81242000

H 6.43561300 -5.57675600 1.32857700

H 3.96031600 -5.58820000 1.35010600

H 2.71138300 -3.48971700 0.85032700

H 0.18050400 -2.08579400 0.52503700

H -2.29348000 -2.09828200 0.52630200

H -2.84175900 -1.62116200 -1.84820700

H -4.15007100 -3.13213300 -3.29779600

H -6.62687100 -3.17426700 -3.14105700

H -7.78494600 -1.63874700 -1.55005800

H -7.78495200 1.63875600 1.55004000

H -6.62688500 3.17425600 3.14106300

H -4.15008800 3.13210800 3.29782700

H -2.84176800 1.62114100 1.84824300

H -2.29347900 2.09828100 -0.52630600

H 0.18050500 2.08579200 -0.52504300

H 2.71138000 3.48972400 -0.85030100

H 3.96031100 5.58821900 -1.35003500
